# Review of the research programme on the *Mortella III* wreck (2010-2020, Corsica, France): A contribution to the knowledge of the Mediterranean naval architecture and material culture of the Renaissance.

**DOI:** 10.12688/openreseurope.13942.1

**Published:** 2022-01-11

**Authors:** Arnaud Cazenave de la Roche, Fabrizio Ciacchella, Fabien Langenegger, Max Guérout, Marco Milanese, Ana Crespo Solana

**Affiliations:** 1 Centro de Ciencias Humanas y Sociales (CCHS), Instituto de Historia (IH), Spanish National Research Council (CSIC), Madrid, 28037, Spain; 2 NavLab Laboratorio di Storia Marittima e Navale, University of Genoa, Genoa, 16126, Italy; 3 Office du Patrimoine et de l’Archéologie du Canton de Neuchâtel (OPAN), Neuchâtel, 2068, Switzerland; 4 Groupe de Recherche en Archéologie Navale (GRAN), Toulon, 83800, France; 5 Department of History, Humanities and Education, University of Sassari, Sassari, 07100, Italy

**Keywords:** Mortella, Mortella wrecks, Ferrara, Boscaina, Corsica wrecks, Mediterranean shipbuilding, Mediterranean naval architecture, Renaissance shipbuilding, Mediterranean 16th century, Renaissance Mediterranean shipbuilding. Early modern shipbuilding, Early modern naval architecture

## Abstract

The Mortella wrecks are the remains of two *navi*, Genoese seagoing merchant ships, sunk in 1527 in the Bay of Saint-Florent (Upper-Corsica, France) during the Seventh Italian War. A programme of archaeological excavations and historical research has been held on one of them, *Mortella III*, between 2010 and 2020. It has involved a multidisciplinary team around a European research project called *ModernShip* (Horizon 2020), whose objective is to shed light on Mediterranean shipbuilding during the Renaissance, a field still little known to this day.

At the end of these 10 years, the aim of the present article is to conclude this research programme with the presentation of a scientific review that complements a recently published monograph on the *Mortella III* wreck. This study presents the latest results on the ship's architecture obtained during the excavation of the wreck in 2019, including a study of the wood of the framework.

Finally, this article broadens our understanding of the *nave* presenting the results of a collaborative line of research on material culture with three studies in close connection with the ship architecture: artillery, anchors and ceramics.

## Introduction

The Mortella wrecks are witnesses to a turbulent Mediterranean history at the dawn of the modern era, characterised by the opposition of European nations in the Mediterranean. They were discovered in Corsica in the Bay of Saint-Florent during a survey programme carried out by the Centre d'Études en Archéologie Nautique - CEAN (Centre for Nautical Archaeology Studies) - as part of the national archaeological map programme. ^1^ The wrecks, named Mortella II and III, were uncovered with a side scan SONAR between 2005 and 2006 at a depth of 48 and 38 metres respectively. ^2^

At the time of their discovery, these sites were characterised by the presence of *tumuli* made up of gravel and ballast stones covering the remains of wooden hulls, and by the presence of archaeological material mainly consisting of artillery pieces, stone shot and anchors. The similarity of the artefacts on the two sites and the same nature of the ballast gravel revealed by the petrographic analyses made it possible to link them to the same historical event. On the other hand, the dendrochronological study situated them in the chronology of the first third of the 16 ^th^ century ( Langenegger, 2020a: 177). Literature research in Italian, French and Spanish archives and libraries has made it possible to identify the ships and the story of their sinking: it details the story of two Genoese *navi ^3^* ( Gatti, 1999, 139–250), the *Boscaina* and the *Ferrara*, wrecked in August 1527 during the Seventh Italian War between the Spain ruled by Charles V and the France of François I ( Cazenave de la Roche, 2020a: 143–151). 

The scientific interest and archaeological potential of the Mortella wrecks, in particular for the knowledge of Mediterranean naval architecture of the early modern period, led to the implementation of a research programme, the main component of which was the study of the *Mortella III* wreck between 2010 and 2020. It led to six excavation campaigns being carried out with the financial support of the European Union, the French Ministry of Culture and the Territorial Communities of Corsica. This research gave rise in 2018 to a project currently underway called ‘ *ModernShip*, the structures of the Early Modern Mediterranean Shipbuilding’ founded by the European programme *Marie-Sklodowska Curie Actions* and hosted and supported by the *Spanish National Research Council* ( *Agencia Estatal Consejo Superior de Investigación Científica*, CSIC).

The Mortella project brought together several universities, institutions and research centres such as the University of Paris-Sorbonne and the *ForSEAdiscovery* consortium ^4^ ( Crespo Solana, 2016 and Crespo Solana *et al*., 2014). It led to the formation of a multidisciplinary and international team whose work has resulted in several publications, including a doctoral thesis and a monograph published in French and English ( Cazenave de la Roche, 2020b and Cazenave de la Roche, 2021), which this article completes with new information from the last excavation campaign.

The study of the *Mortella III* wreck was held around three main lines of research: the first devoted to naval architecture, the second to material culture (artillery, anchors, ceramics and other artefacts) and the third to literature research. From an archaeological point of view, given the nature of the site, it was naturally the first line on naval architecture that was favoured.

This research was underpinned by a spatial issue for which the major challenge is to contribute to documenting a model of Mediterranean naval shipbuilding of Italian influence at the beginning of the 16th century. It seeks to highlight knowledge in the form of ‘technical fingerprints’ ^5^ and comparing them with those identified in the Atlantic area, and more particularly in the ‘Ibero-Atlantic’ area, from the end of the 1980s ( Oertling, 1989b, Oertling, 2001, Castro, 2008). It should be pointed out that our study also sought to take into account the presence of ‘architectural markers’, i.e. the geometric specificities of the construction in terms of proportions and shapes, which are essential and still largely absent from the studies carried out so far.

At the end of the 15 year period since the discovery of the Mortella wrecks, this article aims to conclude the research programme with a double objective. The first is to present a synthesis of the architecture of the Mortella ship, incorporating the new information provided by the latest excavation of the wreck carried out in 2019. The aim is to highlight important technical and architectural aspects that may shed light on Mediterranean shipbuilding as proposed by the *ModernShip* project. This contribution to the knowledge of Mediterranean technical culture is an essential milestone in understanding the structures of European shipbuilding in the early modern period. The architectural approach includes a dendrochronological analysis of the wood carried out by co-author, Fabien Langenegger. Furthermore, the second objective of this article is to review the contribution of the wreck to the material culture of the 16th century, a rich and as yet unpublished line of research. It has been the object of a collaboration stimulated by the *ModernShip* project with several specialists: Fabrizio Ciacchella for the anchors, Max Guérout for the artillery and Marco Milanese for the ceramics. These three topics are closely connected to the ship and linked to her architectural characteristics. In this sense, they contribute to the completion of this project by broadening its perspectives as much as possible.

## 1. Overview of the *Mortella III* wreck: arrangement of the site, dating and history of the ship

### 1.1 General arrangement of the site

**Figure 1.**
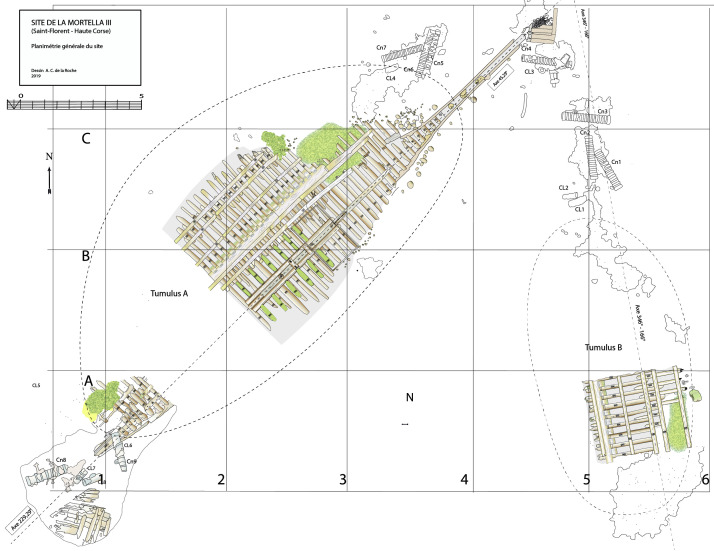
General planimetry of the *Mortella III* wreck.

The *Mortella III* site has a particular arrangement with the presence of two *tumuli* that form two distinct archaeological sets. These two areas, named A and B, were more than 30 metres apart in the south-western sector of the site and meet in the north-eastern part ( Figure 1). This dual layout initially raised the question of whether we were dealing with a wreck whose structures had become separated, or on the contrary, of two vessels that had sunk side by side. The excavation of a portion of *tumulus* B ascertained the existence of a single wreck fractured in a longitudinal direction along the port end of the floor-timbers. The two edges separated before reaching the seabed, perhaps as a result of the shock wave caused by the ship's stern hitting the bottom, thus explaining the presence of two *tumuli*.

*Tumuli* A and B were 35 × 12 metres and 20 × 10 metres respectively, covering an area of just over 600 m ^2^. They were rising a little more than one metre above the general level of the seabed and were covered by a thick layer of ballast gravel overlaid by river pebbles that masked the remains of the hull. Their nature (sandstone with calcite veins) is typical of the pebbles that form the beaches of the Genoa area. The seabed around the site is composed of thick, deep mud with a northward sloping gradient of about 2%.

The first remnants of the hull which are visible on the northern side of tumulus A correspond to the burnt ends of frames which leave no doubt as to the violent fire that consumed the ship before she sank. It should also be noted that at the time of the discovery of the site, in the north-east zone, the aft part of the keel emerged from the *tumulus* A over approximately five metres up to her heel.

Finally, the scarcity of the archaeological artefacts present on the site testifies to the fact that it is likely that the crew abandoned the ship, taking with them what they could before setting fire to her. We will come back to these circumstances in the section dealing with the historical episode. The most representative part of the artefacts was the wrought iron artillery, nine tubes and eight breech chambers scattered around the site, together with numerous stone shot. The ropes found in large quantities and partly burnt in the fore area of the ship, and finally the on-board ceramics found very fragmentary and also altered by the heat in the stern area.

### 1.2 The historical context and the shipwreck episode

The excavation of the *Mortella III* wreck was accompanied by a programme of literature research in various archives and libraries which brought to light the story of the shipwrecks, two Genoese *navi* sunk in 1527. This historical event, reported by two Italian authors, Paolo Giovio ( Giovio, 1555) and Agostino Giustiniani ( Giustiniani, 1537), proved to be in perfect coincidence with the archaeological evidence and dendrochronology. It was therefore retained for the identification of the wrecks.

***1.2.1 The historical context of the year 1527 and the origin of the shipwrecks*.** This year was the scene of the confrontation between France, whose admiral of the fleet in the Mediterranean was the Genoese Andrea Doria, and Spain which, paradoxically, was allied to Genoa. After the fall of Rome in May and its pillaging by the Spanish, the French fleet allied itself with that of Venice and the Pope under the name of the ‘League of Cognac’. It then organised a blockade of the city of Genoa, which quickly found itself in a situation of famine. In this context the Genoese sent several ships to try to supply the starving city with grain, and two of them, chased by French galleys, scuttled in the Bay of Saint-Florent.

Giustiniani, who witnessed the event at first hand, reports it as follows: the two Genoese *navi* had been sent to Sicily to load up with wheat and attempt to supply Genoa, hoping that they would succeed in overcoming the blockade of the city. When they stopped over in Corsica, in the bay of Saint-Florent, they were surprised by a fleet of French galleys. Trapped by the lack of wind and faced with the danger of being captured by the enemy fleet, the Genoese decided to abandon their ships after setting fire to them to prevent them from falling into French hands. It is understandable in these circumstances that the *navi* were unloaded in a hurry and - in these circumstances - it is also understandable that due to the lack of time to disembark them, heavy artefacts such as anchors and cannons were left on board.

***1.2.2 Identification of the ships*.** The texts provide an unusual piece of information: the names of the ships and their port of origin. One was called the *Ferrara* and the other the *Boscaina*. They were attached to the port of Rapallo, located about twenty kilometres south-east of Genoa. The two names *Ferrara* and *Boscaina* can be associated, as was the custom in Genoa in the 16 ^th^ century, with a patronymic which, in addition to the official name of the ship (often associated with a saint), usually referred to her owner, turning his family name to the feminine ( Calegari, 1970, 21). *Ferrara*, associated with the name *Ferraro*/ *Ferrari*, is common in Italy and its etymology evokes the profession of a blacksmith.

As for *Boscaina*, the situation is similar: Her name seems to be linked to the patronymic of a noble family from the North of Italy, whose name was *Boscaino*/ *Boscaini*. Etymologically it was associated with the term *bosco* which evokes a small forest. Another hypothesis was also put forward which suggested a possible link between the word *Boscaina* and the Spanish province of Biscay. The word could be linked to the terms *Viscaya* or *Biscaya* in Spanish: the Basque Country. In this case, the *Boscaina* could also owe her name to the origin of her owner, or that of the ship, sometimes used to call her. However, it should be noted that in his text, P. Giovio calls ‘ *la Rapallina’*, the *nave* that accompanied the *Ferrara*, thus linking her to the port of Rapallo ( Giovio, 1555, vol. II, 94).

## 2. Stakes of the excavation and working methodology

### 2.1 Stake of the excavation

The knowledge of modern naval architecture reached an important stage in the late 80’s with the first characterization of 16 ^th^ century ‘Ibero-Atlantic’ shipbuilding drafted by Thomas Oertling ( Oertling, 1989b and Oertling, 2001). Together with the contribution made by this work to define an architectural model for the ‘Atlantic’ nautical space, it has paved the way for a better understanding of the spatial lay out of European shipbuilding, where two different technical cultures coexisted, the first called ‘Atlantic’ and the second called ‘Mediterranean’. Over the last 30 years however, little progress has been made in describing these models, particularly the Mediterranean one, due to a lack of wrecks.

The discoveries of the Mortella wrecks together with recent discoveries of other wrecks of Mediterranean constructive tradition dated from the 16th century have changed this situation ^6^. The prospect of the analysis of this archaeological data in conjunction with that of the two already documented wrecks of Mediterranean origin: the *Lomellina* wreck ( Guérout *et al*., 1989), and the Calvi I wreck ( Villié, 1989; Villié, 1990 and Villié, 1991) opens the way to set the milestones of a Mediterranean technical model, of Italian influence in particular. The study of the architectural remains was carried out with a major stake in mind, which is to contribute to documenting a constructive and architectural model for the Italian-influenced Mediterranean maritime space in the 16 ^th^ century. In other words, all the issues addressed during the excavation of the *Mortella III* wreck involved a spatial questioning: this is an essential aspect of our approach. Thus, the portrait of the *nave* that the excavation of the wreck has sketched out contributes to documenting Genoese naval construction, which can be provisionally included in an ‘Italo-Mediterranean’ set.

Beyond the knowledge of Mediterranean shipbuilding, it is a matter of understanding these two ‘nautical spaces’ in continuous interaction since the Middle Ages and of trying to set them out in all the complexity of their exchanges and technological transfers.

### 2.2 Methodology of the work

***2.2.1 Research method*.** The programme of excavation and study of the *Mortella III* wreck was implemented with three lines of research. The first, the line on naval architectures, was defined as a priority with regard to the good condition of the remains of the ship’s hull. From a methodological point of view, our research focused on highlighting technical ‘fingerprints’ and ‘architectural traits’ characterizing the construction of the hull. It eventually aims to lay the foundations for a model of shipbuilding that belongs to the Mediterranean technical culture. It is useful to specify that particular importance was attached to conducting this study by clearly separating the observations made on the construction methods, i.e. all the carpentry techniques implemented, namely: the modes of connection of the timbers of the framework (types of assembly, fastening methods), as well as all the carpentry, caulking and sealing techniques more generally. The other concerns the architecture of the ship itself in the true sense of the term: the relationship between her main dimensions, i.e. her proportions, on the one hand, and her shapes, on the other, which together determine her geometry. It was the combined knowledge of these two technical areas that allowed a complete understanding of the construction of the ship.

The second, the material culture line, initially strongly dominated by the artillery and anchors, visible at the time of the discovery of the site, as well as the ship’s ballast, was naturally completed by the artefacts uncovered during the excavation campaigns, i.e. the ceramics and ropes, but also other categories of minority objects that appeared during the excavation (glass, rigging components, etc.). 

Finally, the third line of literature research was set up to document the history of the Mortella ships and their sinking. In our working methodology, the historical information gathered in the texts studied was put in parallel with the archaeological evidence. This interrelationship between archaeology and history is an important aspect of our project in line with the methodological and theoretical approach as developed by Ana Crespo Solana ( Crespo Solana, 2019). It has allowed us to figure out with a high degree of probability the identity of the wrecks.

***2.2.2 Excavation method*.** *Topographical survey*: It was carried out on the basis of two reference systems. A first geodetic network of points was set up by trilateration. It was used as a reference to set a 30m × 30m square containing the entire site. This square was further subdivided into 36 5m × 5m squares set by seven vertical lines running north/south and six horizontal lines running east/west, each 5m apart. In a second step, a line was drawn along the keel along a diagonal axis of 45°-225°. Once this grid was completed a secondary referencing system was put in place. This consisted of a removable grid set up during each campaign in the excavation area and referenced to the network of primary points. This grid, fixed on sliding feet, was composed of aluminium templates forming two 350 × 350 cm squares. Once levelled with spirit levels, it was used as a support for multiple tasks:

- to precise off-set positioning of objects/structures in the framework within the frames;

- to produce longitudinal/cross-sectional drawings with a vertical ruler placed on the carriage;

- to create photo mosaics inside the frames with a plate fixed to the carriage.

The topographic survey was completed by a photomosaic composed of thousands of photos completed each year between 2010 and 2020. In order to obtain a document to scale, it was post-processed to correct lens distortions and to align the images with our topographic survey. The photomosaic considerably improved the quality of our observations ( Figure 3). Then a 3D imaging coverage ( Agisoft, Metashape) of the site was carried out by the Maritime Archaeology Trust (MAT, UK) ( Figure 8).

*Methods of intervention*: Diving was carried out with air and decompression stops using pure oxygen. The sediment was cleared with an air dredge during the removal of the sterile layer of ballast gravel, then by water dredging in the layers close to the hull.

## 3. The line on naval architecture

### 3.1 Arrangement and construction methods of the framework ( Figure 4)

Beneath tumulus A, the remains of the oak hull of a ship carvel built were uncovered oriented north east/south west. The heel of the keel was located in the northern part, the fore end of the keel in the southern. The longitudinal axis formed by the keel and the keelson was preserved along its entire length. On the starboard side of the wreck, the half frames formed by the sequence ‘floor-timbers/first-futtock/second futtock’ were preserved, although their ends were heavily charred. 

***3.1.1 The transverse framework***

**Arrangement**

In a classical way, starting from stern to bow, the first-futtocks were attached to the aft side of the floor-timbers and the second-futtocks up to the master-frame, which was identified as the 27 ^th^ from the stern (M27). Then the sequence was reversed with attachment to the fore side.

The frames were broken at two levels along the length of the wreck: 

- on the starboard side, at the first-futtock/floor-timbers union, which resulted in a 20–30° collapse of the frame;

- on the port side, where only the ends of the floor-timbers, with some broken, had been preserved.

The architectural set found under tumulus B was symmetrical to that observed under tumulus A. The first remains were a series of floor-timbers with the ends of their first-futtocks preserved over 20 to 40 cm. On the other side, the ‘floor-timbers/first-futtock/second-futtock’ sequence was preserved, although a significant portion of the second-futtock had been burned. As the first-futtocks were attached to the aft sides of the floor-timbers, the set excavated (AF/12B) clearly appeared to belong to the fore part of the wreck ( Figure 1).

**Figure 2.**
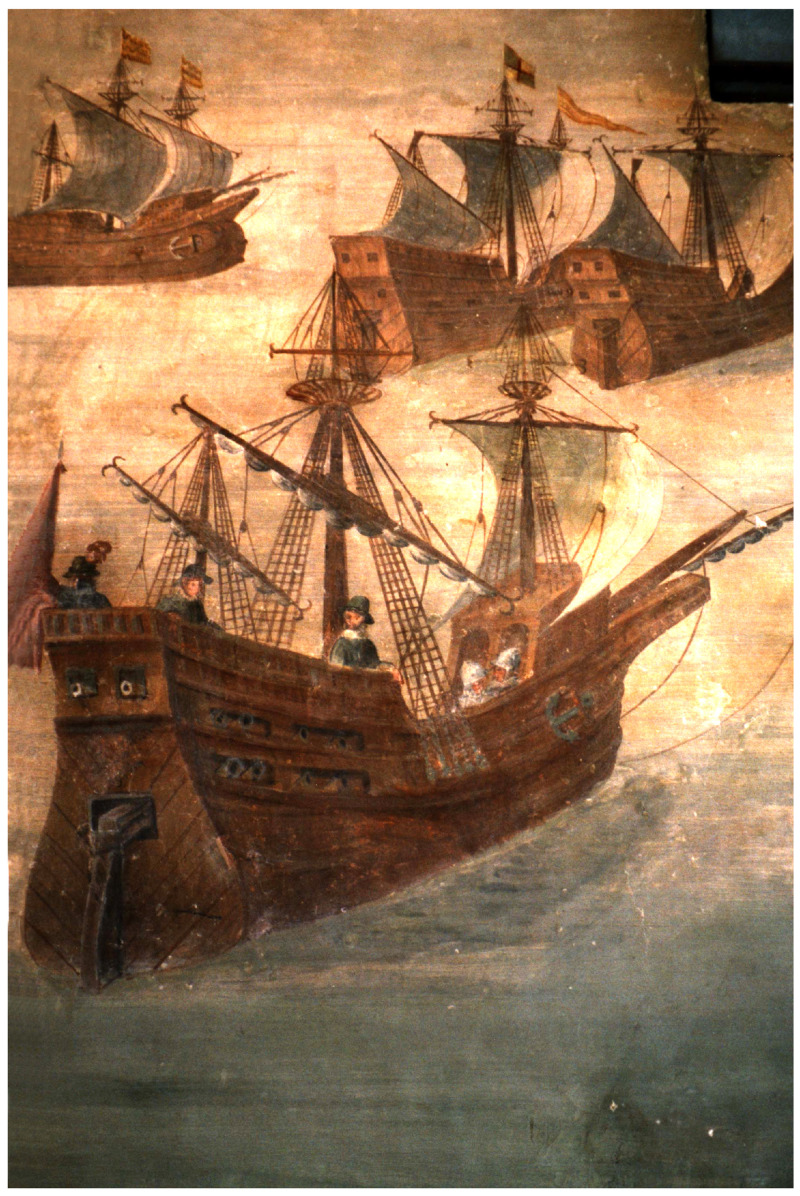
A Genoese navi in the 16 ^th^ century Fresco by Giovanni Baptista ‘The Genoese’, castle of Alvaro de Bazán, Santa-Cruz, Spain.

**Figure 3.** Photomosaic of the site of the Mortella IIII site – © All rights reserved, Christoph Gerigk.

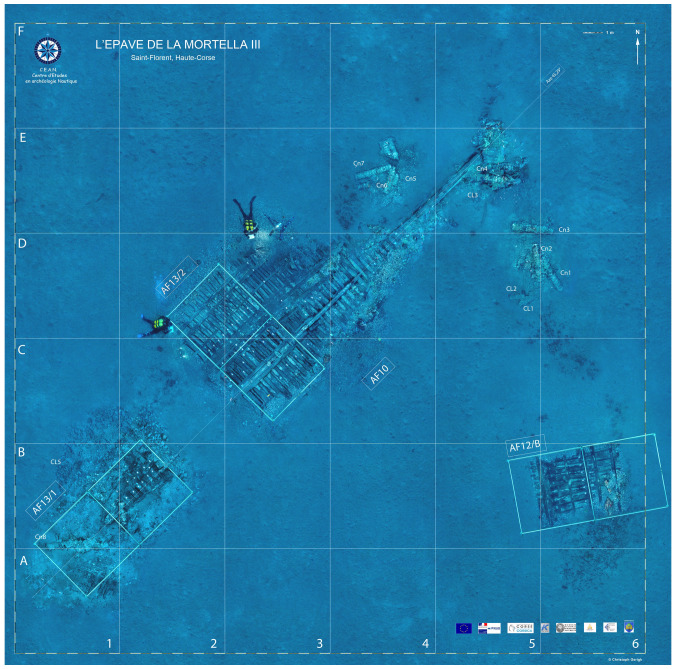

**The wood**

The wood samples taken from the pieces of the transverse framework were mainly identified as sessile oak ( *Quercus petraea*) although some samples coming from second-futtocks and planking were pedunculate oak ( *Quercus robur*).

**Dimensions and intervals**

The main dimensions of the transverse framework were taken from the 41 frames uncovered in *tumulus* A. In order to calculate meaningful averages, they were divided into three groups:

- The fore crutches

- The framing at midship: 19 from M16 to M34. M27 was identified as the master-frame. This group therefore had seven frames upstream of the master (which marks the limit of the excavated area) and 11 frames after the master-frame.

- The frames to the aft of M16 - this boundary had been placed in the area which, in view of the proportions of the ship, would have corresponded more or less to the tail-frame location.

The main averages of the measurements taken according to this scheme are summarised in Table 1. They can be commented on as follows:

*Intervals*: The ‘room and space’, i.e. the distance between the centre of each floor-timber, was on average 33 cm in the central area, an interval that tended to increase towards the stern (36 cm on average) and towards the bow (34.3 cm on average). These values are quite similar to those found on the Red Bay wreck ( Loewen, 2007, III, 62–64). As for the single interval, i.e. interval between each floor-timber, it was on average 17.7 cm for the midship group. In this part, the crossing between floor-timbers and first-futtocks formed an almost continuous whole.

*The floor-timbers*: They were generally square in cross-section in the midship area where their average height was 17.3 cm and their average moulded dimension was 16.7 cm, measured at the level of the starboard side of the floor-timber. The average width of the floor-timbers of the forward and after groups was 20 cm.

The master floor-timber had the largest span, at 4.04 metres. The length of the floor-timbers gradually decreased to about 3 metres in the tail-frame area. For the aft and fore groups, the timbers were in too poor condition to take measurements.

*First-futtocks*: Their length varied between 3.20 and 3.70 metres in the midship area. Aft and fore pieces could not be measured accurately. The average moulded dimension of the midship area group was 15.3 cm and sided was 13.7 cm. The moulded dimensions of the pieces tended to decrease towards the stem (12.7 cm), towards the stern there were too few pieces to draw any conclusions.

*Second-futtocks*: Their sided/moulded dimensions were 15.5 cm. Since part of the timbers had been burnt, it was not possible to measure their length.

**Figure 5.**
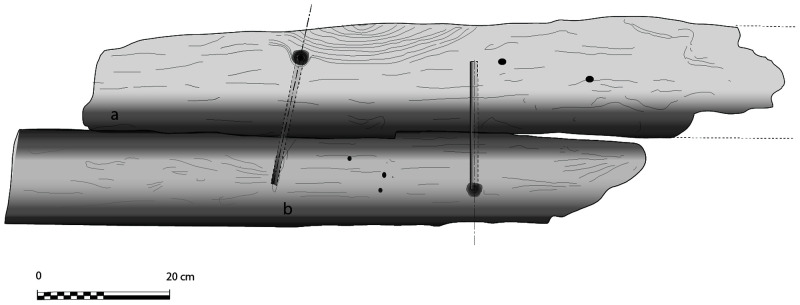
Hook scarf and circular iron nails attaching floor V18 to first-futtock G18.

**Connection of the frame-timbers** ( Figure 5)

*Assembly*: Between the tail frames, the scarfs of the floor-timbers to the first-futtocks were characterized by a single hook of about 15 mm. The contact surfaces were carefully levelled and overlapped over a length of 1 metre, on average. This typology of scarfs is known as ‘hook scarf’, a method of attaching timbers typical of the Mediterranean shipbuilding tradition ( Cazenave de la Roche, 2020c, 66 and 154).

*Fastening pattern:* The timbers were fastened together by two circular iron nails about 12 mm in diameter. They were driven through the first piece and ended up into the wood of the second one. The nailing was alternated: the first nail was driven from the floor-timber to the first-futtock, the second from the first-futtock to the floor-timber. This fastening pattern is similar to that observed at Villefranche ( Guérout *et al.,* 1989, 43). A peculiarity of the nailing of the floor-timbers to the first-futtocks was that where the first nail had been driven horizontally, conversely the second nail, closest to the end of the piece, had been driven obliquely from the top of the floor to the bottom of the futtock side. This nailing pattern suggests that the first-futtocks were pre-assembled to the floors by a single nail, the second being driven afterward.

The connection pattern (scarfs and fastening) observed between the first and second-futtocks were identical to that of the floor-timbers to the first-futtocks.

*Beyond the tail-frames*: In the aft area of the ship, the connections between floor-timbers/crutches and first-futtocks were no longer made with a hook but by a simple juxtaposition of the pieces. In the fore part, observation couldn’t be made, as the futtocks didn’t survive.

**Table 1.** Summary of framework main measurements.

Table 1.A – Summary of the framing measurements										
Location / Timbers (measurement in metres)	Frames		Floor-timbers			First-futtocks			Second-futtocks	
	Room & Space ^1^	Interval ^2^	Length	Moulded	Sided	Length	Moulded	Sided	Moulded	Sided
**FORE AREA: Averages M1P** ** to M8P**	0.34	0.12	X	0.21	0.32	X	0.13	X	X	X
**MID-SHIP AREA: Averages ** **M9 to M34**	0.33	0.18	3.47	0.17	0.17	3.46	0.15	0.14	0.15	X
**AFT AREA: Averages M1** ** to M8**	0.35	0.16	X	0.20	X	X	0.13	X	X	X
Note (1): Room & space: measure of the distance from the center of a floor-timber to the other. Note (2): Interval: measure of the distance between each floor-timber.										

Table 1.B - Summary of master-frame M27 measurements										
Measures (m) / timbers of M27 (metres)			Floor V27		First-futtock G27 A		First-futtock G27 B		Second-futtock A27	
**Lenght of the timbers**			4.05		3.34		3.36		1.80	
**Moulded / sided**	Sided average		0.18		0.132		0.148		0.155	
	Moulded average		0.15		0.15		0.15		0.148	

Table 1.C - Summary of longitudinal timbers measurements at midship										
*Measures in metres*	Lenght		Moulded / Width		Sided / Thickness					
Upper keel (MA.01)	25		0.24		0.26	0.46				
Lower keel (MA.02)					0.20					
Keelson			0.20 to 0.22		Average. 0.14					
Clamps			Average 0.16		Average. 0.14					
Planking			0.16 to 0.20		0.08 to 0.09					

***3.1.2 The longitudinal frame***

**The keel**

**Figure 6.**
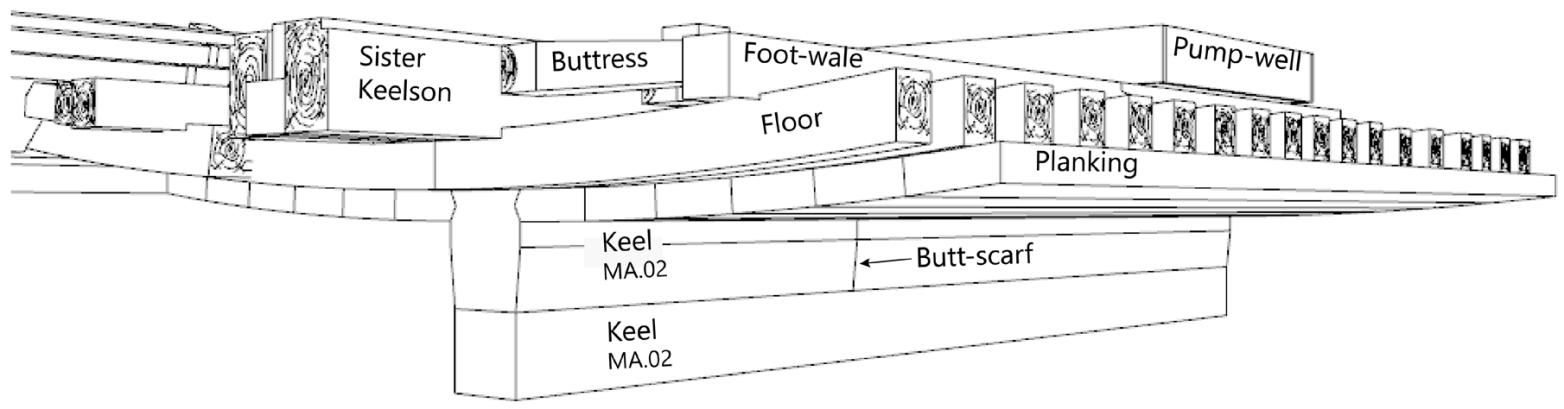
Dual morphology of the keel and butt scarf. Drawing credit: Jesús Guevara.

Emerging from the sediment, the heel of the keel was spotted when the site was discovered. However, it was only much later, with the excavation of the stern area that its fore end was located. Its length was measured at 25 metres, giving a total estimated length of 26 metres ^7^. However, no trace of the stem-post could be found. The discovery of a butt-scarf used to attach two pieces of the keel at midship highlighted its dual morphology. These two characteristics are demonstrative of the originality of this architectural device. With regards to the scarf of the keel ( Figure 6), the type of assembly used to join the two pieces of keel, called butt-scarf, consists of a simple positioning of the timbers end to end. At first sight, the weakness of this assembly used for a structural timber as mechanically important as the keel may be surprising. However, a study of the texts shows that this assembly technique was not an exception in the history of shipbuilding and that it was even a rule, perhaps originating from the Mediterranean tradition, which spread in the 16th century: from the beginning of the 17 ^th^ century the Spanish Ordinances generalised its use for all ships built in Spain ( Hormaechea *et al.*, 2018, vol.II, 71). Cayetano Hormaechea cites three other Spanish authors of the 17 ^th^ century who advocate this system: Juan de Amassa in 1635, Diaz Pimienta in 1645 and Francisco Garrote, who in 1691 gives the better sealing that this type of scarf makes possible as an explanation for this choice ( Garrote, 1691). As the keel is in contact with the water, it is understandable that a butt-scarf would generate the same principle of sealing through the swelling of the wood as it is used for the planking in the carvel technique. From the French perspective, we can also quote a manuscript from 1691 which describes this system of union for the construction of galleys ( Anonymous, 1691).

**Figure 4.** *Mortella III* framework at midship – © All rights reserved, Christoph Gerigk.

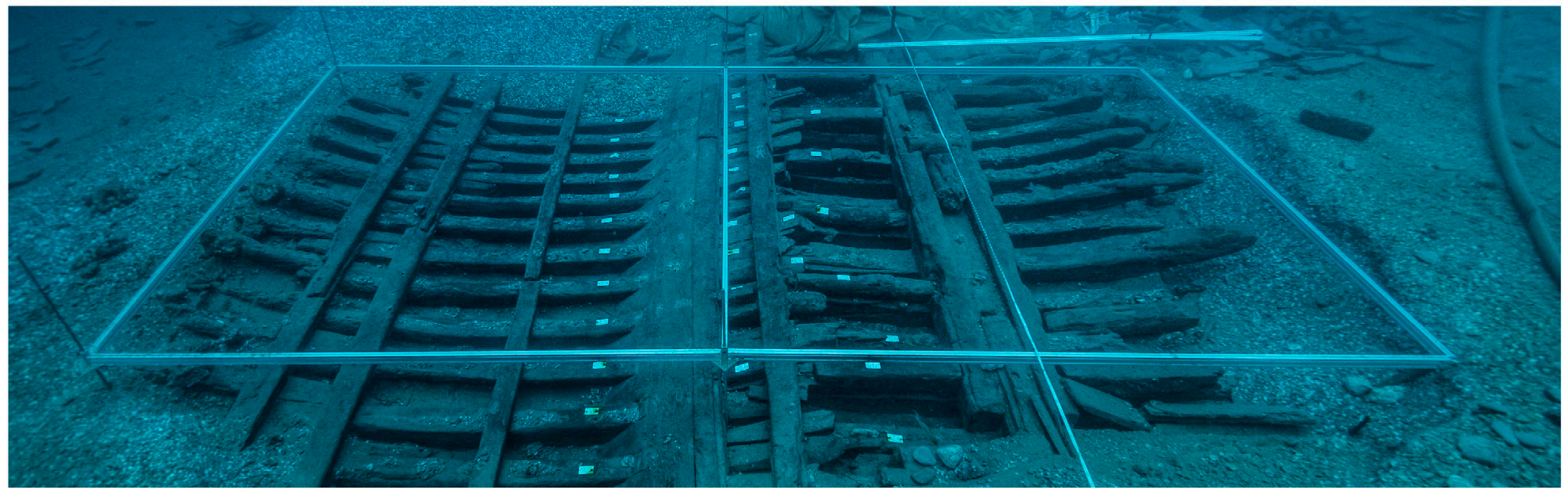

Archaeologically, the system of joining keel pieces end to end was observed on the *Cais do Sodré* wreck ( Rodrigues *et al.,* 1998). It is also found on the *Lomellina* wreck at the level of the union of the keel with the heel. However, in this case, its particularity is to be associated with the hook-scarf located in the upper part of the piece ( Guérout *et al*., 1989, 26–27).

The dual morphology of the keel ( Figure 6) provides an answer to the problem of the mechanical weakness of the assembly that we have just described: the keel is not made up of one timber, but of two strong timbers of similar size. In this technical system, the upper piece played a predominant role for two reasons. The first is that it was provided with a rabbet running along its sides from stern to bow in which the strakes of the garboards were fitted. The second is that its heel was shaped to receive and accommodate the lower part of the sternpost. Therefore, in a strict sense, one could consider that the upper piece (named MA.01 in our inventory) is the keel of the ship and the lower part (MA.02), a protective/reinforcing piece that could be assimilated to a false-keel. Nevertheless, the dimensions of this timber are very close to the upper piece, in particular with regard to the scarfs that it covers, and its function plays a mechanical role that goes well beyond the simple protection for which a false-keel is intended in a ‘sacrificial’ manner. For this reason, in our opinion, the MA.02 piece should be considered as a component of the keel in its own right.

The technical solution adopted on the *Mortella III* ship, which combines butt-scarf with a double keel, seems to have become permanent over time. In any case, there are texts that still recommend it in the 18 ^th^ century. This is the case of the French author Duhamel du Monceau who writes that perhaps there would be no disadvantage to match the keel pieces end to end, by doubling the scarfs with the false-keel ^8^ and keelson pieces ( Duhamel du Monceau, 1752, cap. II, art.9).

At midship the upper piece (MA.01) was 24 cm moulded and 26 cm sided. The lower piece (MA.02) was 24 cm moulded and 20 cm sided. The total sided dimension was MA.01 + 02: 44 cm. In the stern area, at 5 metres from the sternpost, both keel pieces were moulded 30 cm and sided 25 cm.

Total sided was MA.01 + 02: 50 cm. At the stern end, while the lower piece preserved the same dimensions of 30 × 25 cm, the upper piece gained height with 38 cm sided. At this point, the keel sided dimension MA.01 + 02 was 25 cm + 38 cm = 63 cm and moulded 33 cm. The rise of the sided dimension of the keel toward the stern is an important characteristic of its morphology.

**Figure 7.**
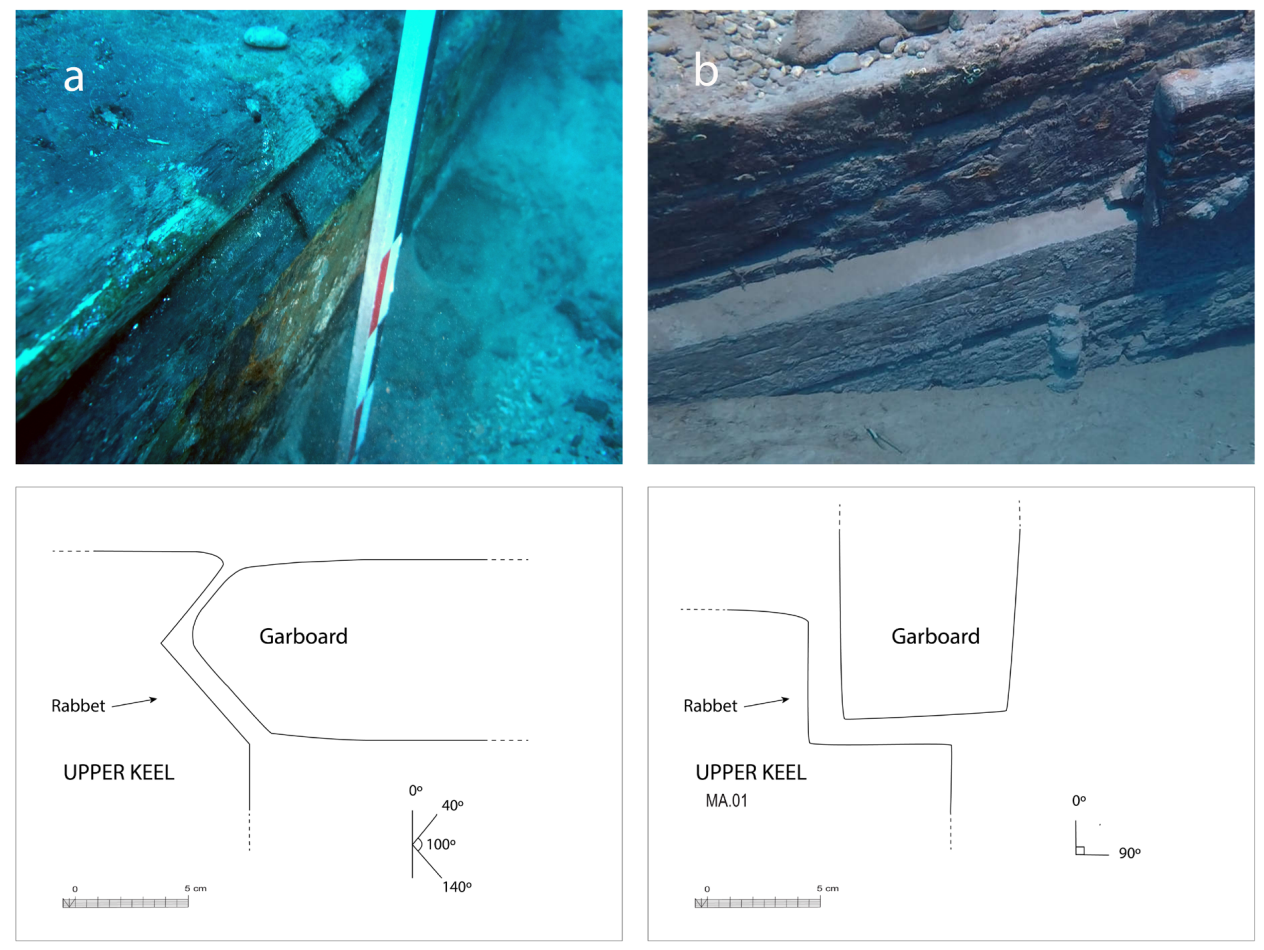
The rabbet. Left ( **a**): Midship area. Right ( **b**): Stern area Photos/Drawings Arnaud Cazenave de la Roche.

The rabbets ( Figure 7), notches whose function is to receive the lower edge of the first planking strake, or garboard, were located on the port and starboard sides of the MA.01 keel piece, all along its upper part. About 60 cm before their aft ends, they continued along the sternpost knee and finally reached the outer faces of the sternpost. However, the degradation of the upper pieces of the stern added to the disappearance of the sternpost and the impossibility to visualize its knee did not allow us to fully pursue our observations.

**Figure 8.**
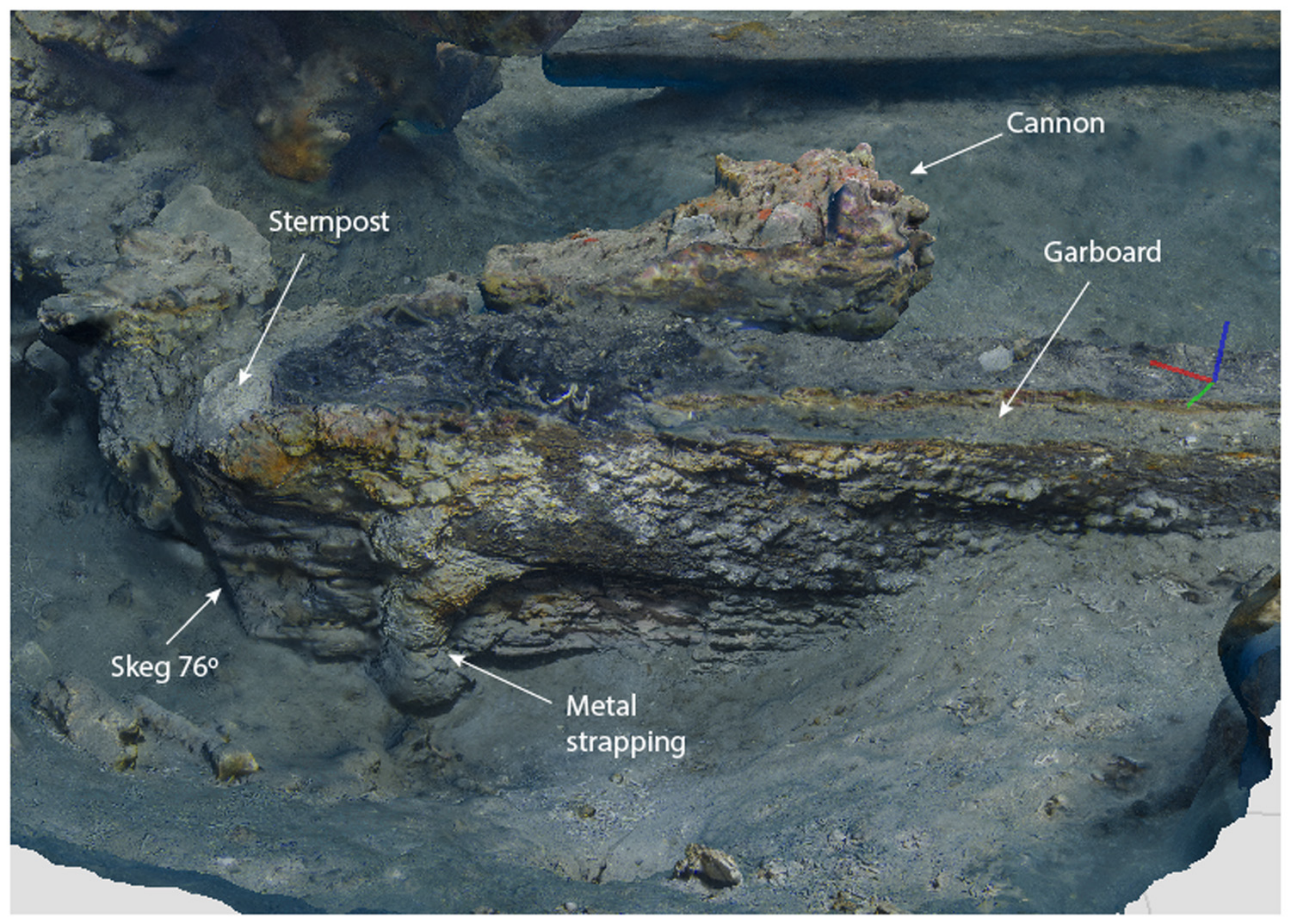
Heel of the keel starboard side. Image excerpted from the 3D photographic coverage of the site.- Brandon Mason, MAT.

The size of the notch varied little. Its height and width were between 5 and 6 cm both at midship and at the stern. Its shape, i.e. its opening, remained the same, with an angle between 90° and 100° between its two edges. However, the position of the notch, i.e. its orientation, changed significantly depending on the position of the garboard: Figure 4a shows how at midship, the notch edges had a position of 40°/140° to the vertical, thus allowing the garboard to connect with the keel in a position close to the horizontal. Figure 4b shows how the rabbet progressively straightened in its course towards the stern to a 0° / 90° position allowing the garboard to be oriented vertically at the stern.

This method of varying the angle of the rabbet from midship to the ends of the keel is different from that observed on the Villefranche-sur-mer, where the angle of the notch was 90° at midship and the degree of inclination of the garboard obtained by its shape ( Guérout *et al*., 1989: 61).

Apart from its dual morphology, a second particularity of the keel lies in the nature of the wood used, since if the upper keel element (MA.01) was made of oak, the lower part (MA.02) was made of alder, a homogeneous cell wood of the genus of *Aulnus*, belonging to the birch family *Betulaceae*.

The heel of the keel was composed of the two keel timbers, the ends of which were cut at an angle of 76°, identical to that of the sternpost. At the level of the upper keel piece, the skeg was shaped from a large branch which provided a base for the sternpost foot. This classic morphology of the heel of the keel is now documented on several wrecks, both in the Atlantic and Mediterranean waters. In fact, it is close to that observed on the *Lomellina* wreck ( Guérout *et al*., 1989, 23–32). 

The notable characteristics of the heel of the keel of the *Mortella III* ship were:

- Firstly, the progressive increase in the height of MA.01 which as mentioned went from 25 cm to 38 cm, i.e. an additional 13 cm at the sternpost departure. Secondly, it was not an assembly piece as is the case for the Red-Bay wreck or *Aveiro 1*, for example, but an integral part of the keel at the end of which it was directly cut, like for the *Lomellina* wreck.
- Secondly, the sternpost attachment system was the same as at Villefranche-sur-mer by means of a notch in the fore part of the heel. This differs from the system observed at Red-Bay, where the beginning of the sternpost was an integral part of the heel, and where the sternpost timber that extended it was assembled by a vertical flat scarf.

**The keelson, the clamps, the ceiling and the planking**

 The keelson is one of the main longitudinal reinforcement pieces of the hull. It was superimposed on the keel and notched over the back of the floor-timbers. It was made of oak and its dimensions were 20 cm moulded and 14 to 15 cm sided.

The clamps were oak pieces with dimensions close to those of the keelson (moulded: 20 cm, sided: 15 cm) notched on the framing ( Table 1C). The visible remains of the hull show five on each side: the first two (S1 and S2) were clamps for reinforcing the attachment of the floor-timbers at the first-futtock (foot-wale and bilge-clamp), the next (S3), were located on the middle of the first-futtocks and the next two (S4 and S5) were the clamps for reinforcing the attachment of the first at the second-futtocks.

On the keelson ran a beech joist. Its upper edges were chamfered and the ceiling, also made of beech ( *fagus sylvatica*), was leaning on it. It was made of planks 30 mm thick and was located in the most central part of the hull between the port and starboard S2 clamps. Beyond that, the inner part of the hull did not appear to be covered with ceiling.

As it was not possible to remove the planking during our excavation, its observation was partial. Samples taken from three strakes in the bottom were found to be oak. However, the weakness of the sample does not allow the presence of other species to be excluded, as is the case for the planking of the Villefranche *nave* ( Guérout *et al*., 1989: 63 and 65) or that of the Red Bay wreck ( Loewen, 2007, III: 111).

The width of the planking varied between 16 and 20 cm. The thickness of the planks fluctuated between 8 and 9 cm at midship and can be compared to that of the Villefranche wreck, whose first 14 strakes were 12 cm thick, and the following ones were 10 cm thick up to the 30 ^th^ strake.

With regards to the fastening pattern, the planking was attached to the frames with round iron nails 10 mm in diameter passing through the planking and the frame from side to side. Two nails, each about 27 cm long, were driven into each frame and the tips of the nails were clenched on it over 3 to 4 cm. This way of fastening the planking to the framing has been observed in the Portugueses shipwreck Nossa senhora dos Martires (1606) ( Castro, 2005). For each nail, a circular pilot hole of 2 to 3 cm in diameter was made on the outside of the planking to allow the nail heads to be driven into the wood.

A study on the caulking and coating materials of the hull was carried out by Carole Mathe ( Mathe, 2020, 197–202). It concludes that the pitch used to coat the planking was mainly composed of pine resin and fat. Unlike the Villefranche-sur-mer wreck, no lead coating was observed.

**Figure 9.**
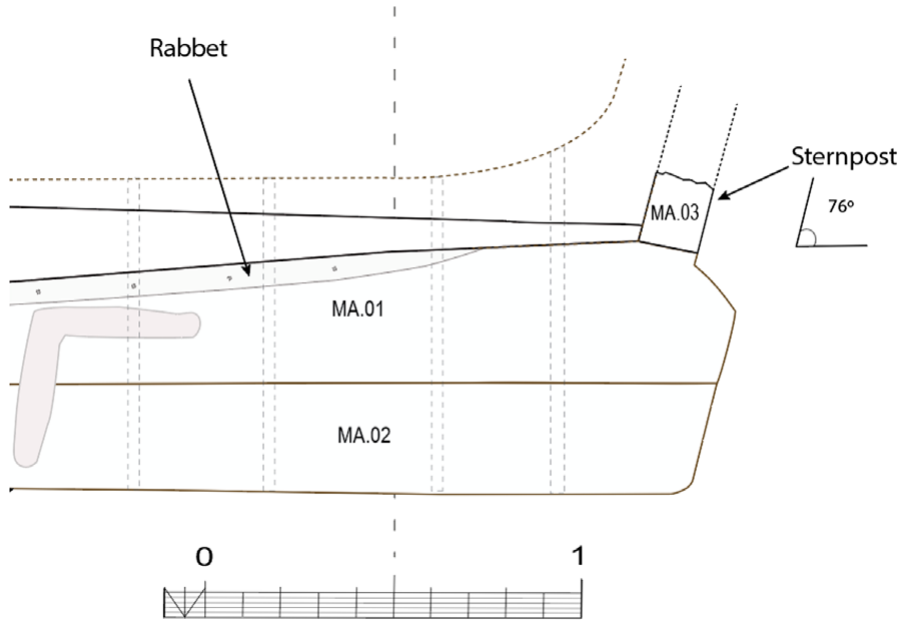
Heel of the keel arrangement. Drawing Arnaud Cazenave de la Roche.

**Figure 10.**
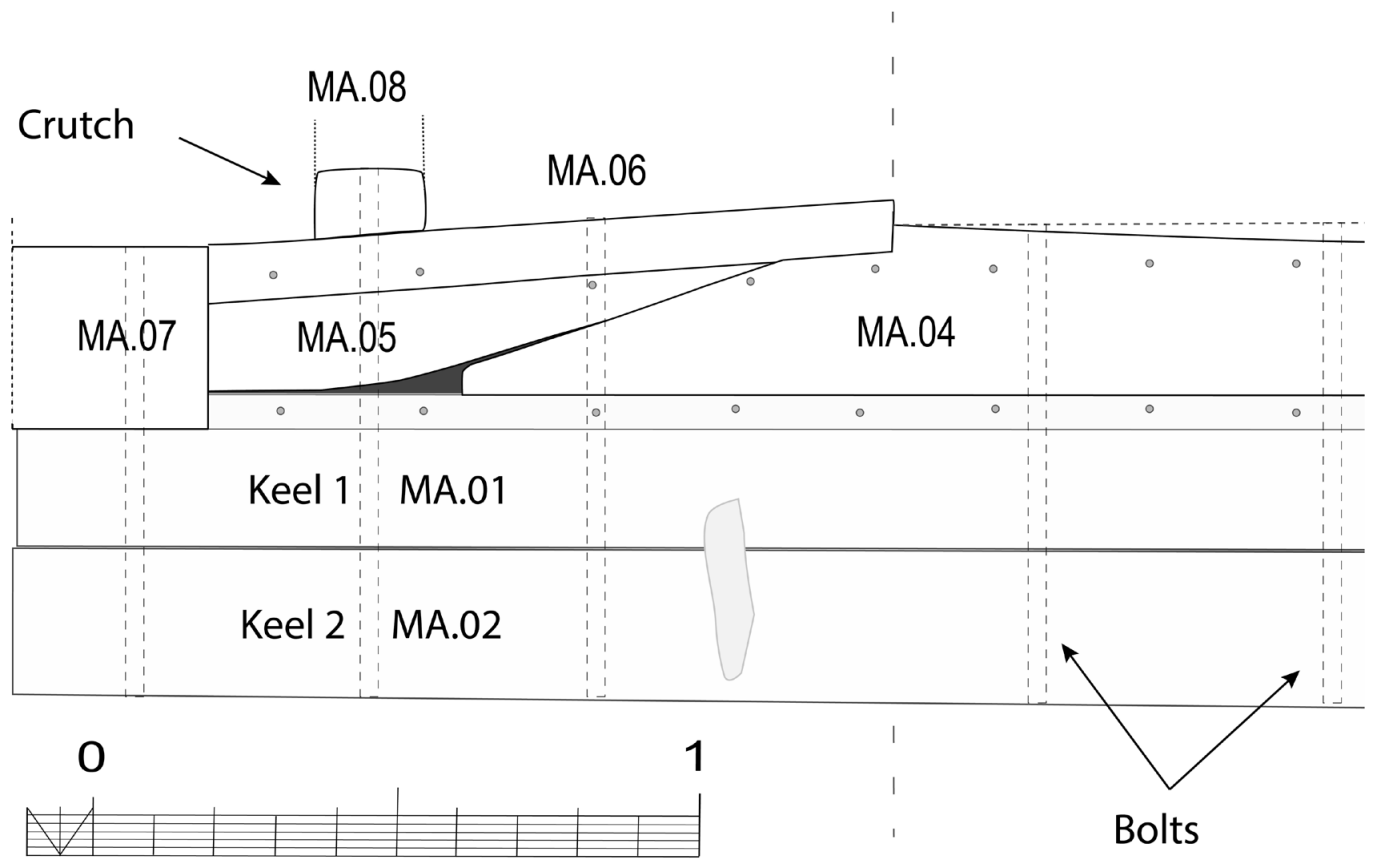
Arrangement of the stern timbers under garboard MA.07.

**Figure 11.**
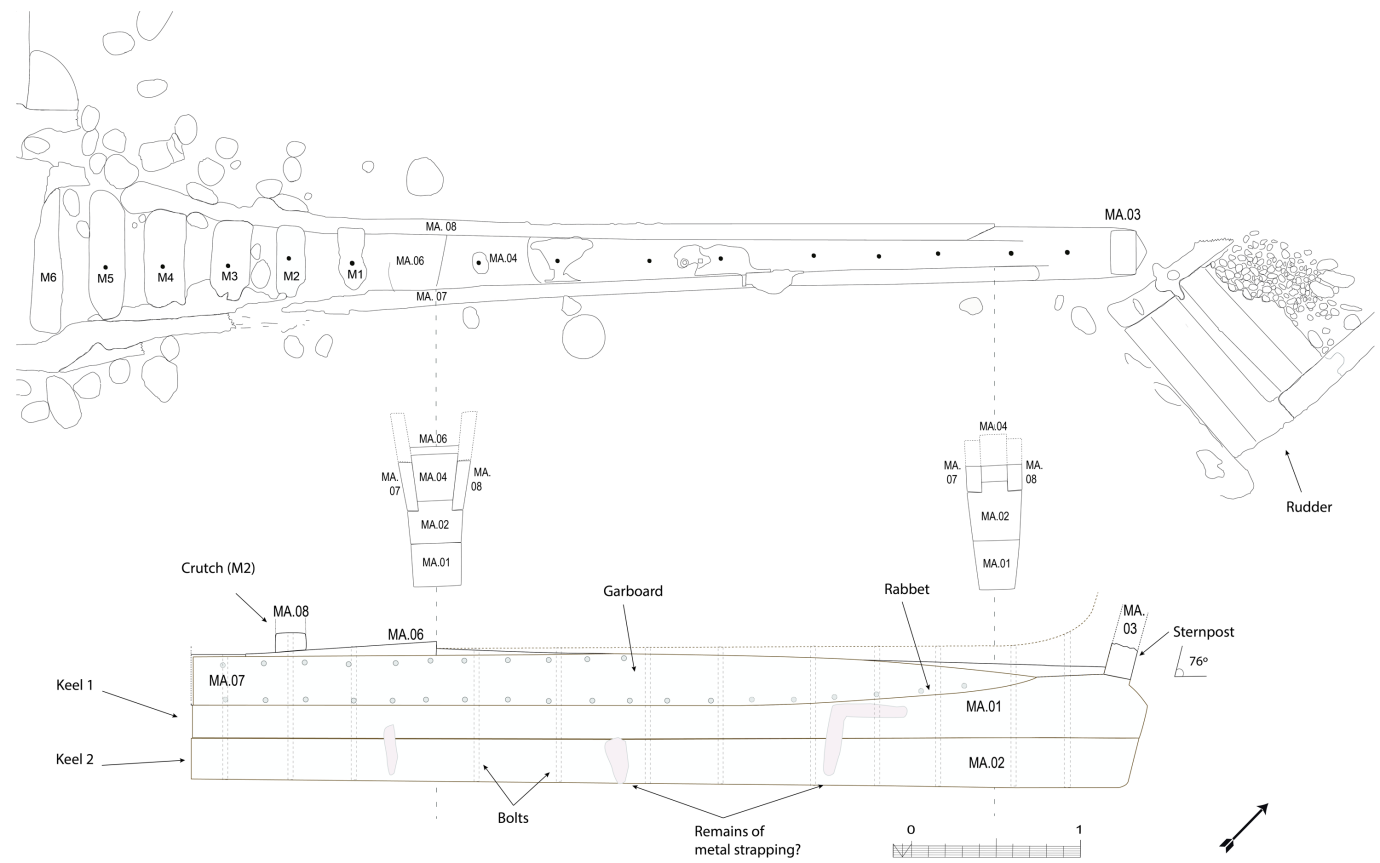
General stern timbers arrangement – Drawing Arnaud Cazenave de la Roche.

**Figure 12.**
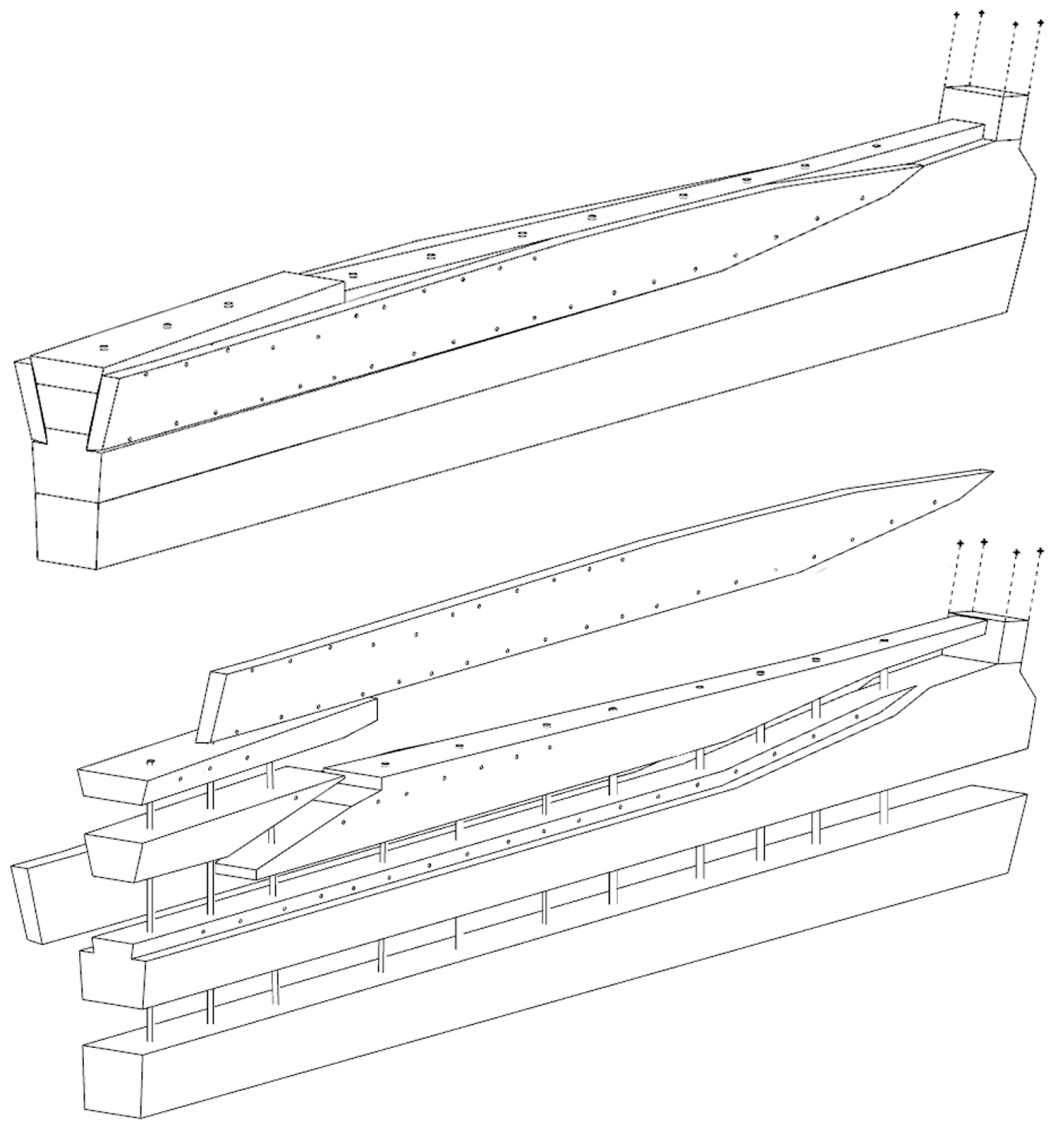
Assembly reconstruction of the stern pieces – Diagram Jesús Guevara.

***3.1.3 The arrangement of the stern ( Figure 9, Figure 10, Figure 11 and Figure 12)*.** The still visible remains of the ship's stern were formed by five massive timbers, arranged longitudinally, including the two superimposed keel pieces (MA.01 and MA.02), followed by three upper pieces (MA04, MA05 and MA06) whose sides were covered by two garboards (MA.07 and MA.08). Then came the remains of the sternpost (MA.03) and the frames, two wooden pieces of which only a few traces remain today. All the pieces were bolted together at regular intervals, indicating the original location of the crutches ( Figure 10). 

The upper timbers could be seen laterally on the port side after the removal of the garboard (MA.07) ( Figure 10). Three pieces of oak were covering the upper face of the keel. MA.04, whose fore part was highly degraded, might be the remains of the sternpost knee. Towards the bow, the last visible remain of the framing was the crutch MA.08 (M1 on the general planimetry of the site). At this stage of the stern, situated 5 metres from the skeg of the keel, the timbers MA.05 (15 cm high) and MA.06 (height: 10 cm, width: 30 cm) were superimposed. At this point, the lower piece MA.05 was joined to MA.04 by a flat horizontal scarf. At its highest point, this piece was 25 cm high (15 cm + 10 cm), but the evolution of its sided dimension, degraded towards the stern, could not be measured. However, a clear morphological characteristic of this piece was the progressive decrease in its moulded dimension from 30 cm at the crutch MA.06 (M1) to 18 cm at the skeg the keel.

For the fastening method all the pieces of the keel (MA.01/MA.02), the upper pieces (MA.04 - 05 - 06) and the frame (MA.06) were held together by iron bolts 3 cm in diameter. Their remains made it possible to locate the original position of the crutches. 

The garboards were made of two oak planks of 28 cm x 8 cm embedded in the rabbets carved in the upper port and starboard sides of the keel. The port garboard (MA.07) that we removed was over 5 metres in length. Its lower edge was quadrangular with a slight bevel towards the outside, leaving room for the insertion of the caulking material ( Figure 7). At the stern, the garboard was almost vertical. It had a slight outward slope that gradually increased towards the bow. As seen in the description of the rabbets, the position of the garboards tilted progressively outwards towards the bow, and their lower edge evolved into a triangular shape allowing an almost horizontal position amidship. The thickness of the garboards (8 cm on average) was slightly greater than the width of the rabbet (6.5 cm).

The garboards were fastened with circular iron nails slightly more than 1 cm in diameter. Their circular heads, with a diameter of 3 cm, were inserted into pilot holes. This fastening method is similar to that of the Villefranche-sur-mer wreck, which constitutes a typically Mediterranean 'technical fingerprint'. The nails were placed at regular intervals along the upper and lower edges of the planks, spaced 20–30 cm apart.

For wood protection and sealing products, pitch was used in large quantities to cover scarfs and connections. In particular, a thick layer of pitch was observed not only on the outside of the garboards, but also poured inside the rabbets. In the course of the excavations, the layers of pitch, which were often more than a centimetre thick, frequently bore witness to their imprint on the pieces of wood that they originally covered. Although the wood that had been exposed to open water had disappeared, the layers of pitch that covered it were still partially visible ( Figure 13).

**Figure 13.** This photo of the fore part of the keel taken at the time of the discovery of the site, shows the remains of pitch in the form of ‘leaves’ that frame the rabbets – © All rights reserved, Christoph Gerigk.

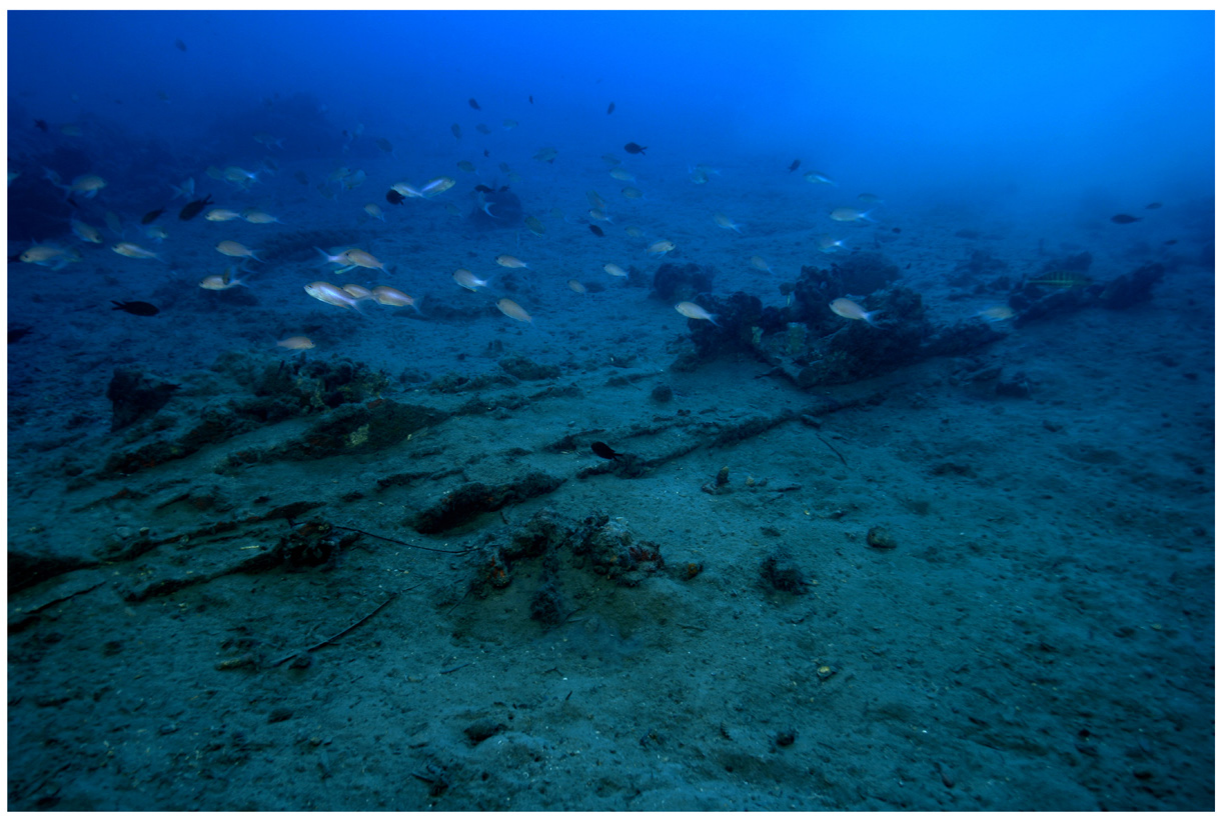

**Figure 14.** The maststep arrangement – © All rights reserved, Christoph Gerigk.

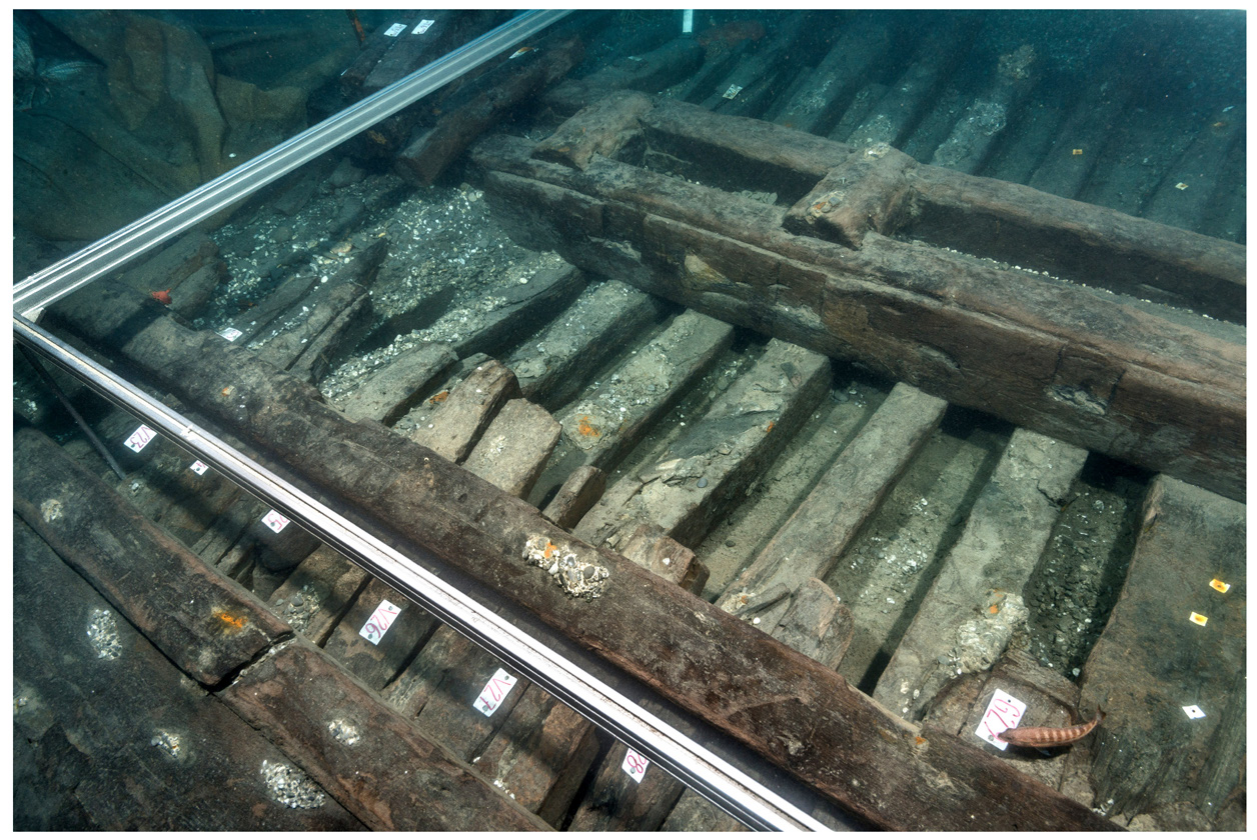

**Figure 15.**
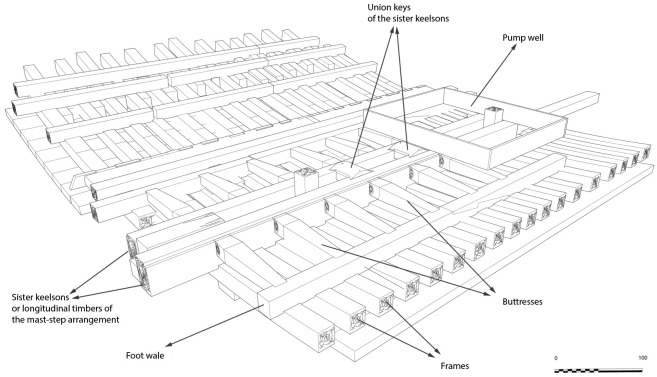
Reconstruction of the maststep arrangement. Diagram J. Guevara.

The maststep is one of the most remarkable architectural works of the *Mortella III* wreck. It was made up of two strong longitudinal timbers, or sister-keelsons, 5 metres long, 30 cm sided and 20 cm moulded. They framed the keelson at midship, where they were notched on the floor-timbers. This typically Mediterranean type of maststep was rarely observed.

The sister-keelsons were attached to the keelson with circular iron nails driven in at an angle and linked together with two wooden keys inserted on their inner sides in dovetail mortises. The space between the two keys was intended to receive the mast foot. The whole structure was strongly maintained laterally by a series of six buttresses placed on each side, arched between the outer face of the sister-keelsons and the first port and starboard foot-wales. These buttresses were made of juniper. This is not a random choice, as it is a Mediterranean species of high mechanical quality and has a particularly high elasticity.

**Figure 16.**
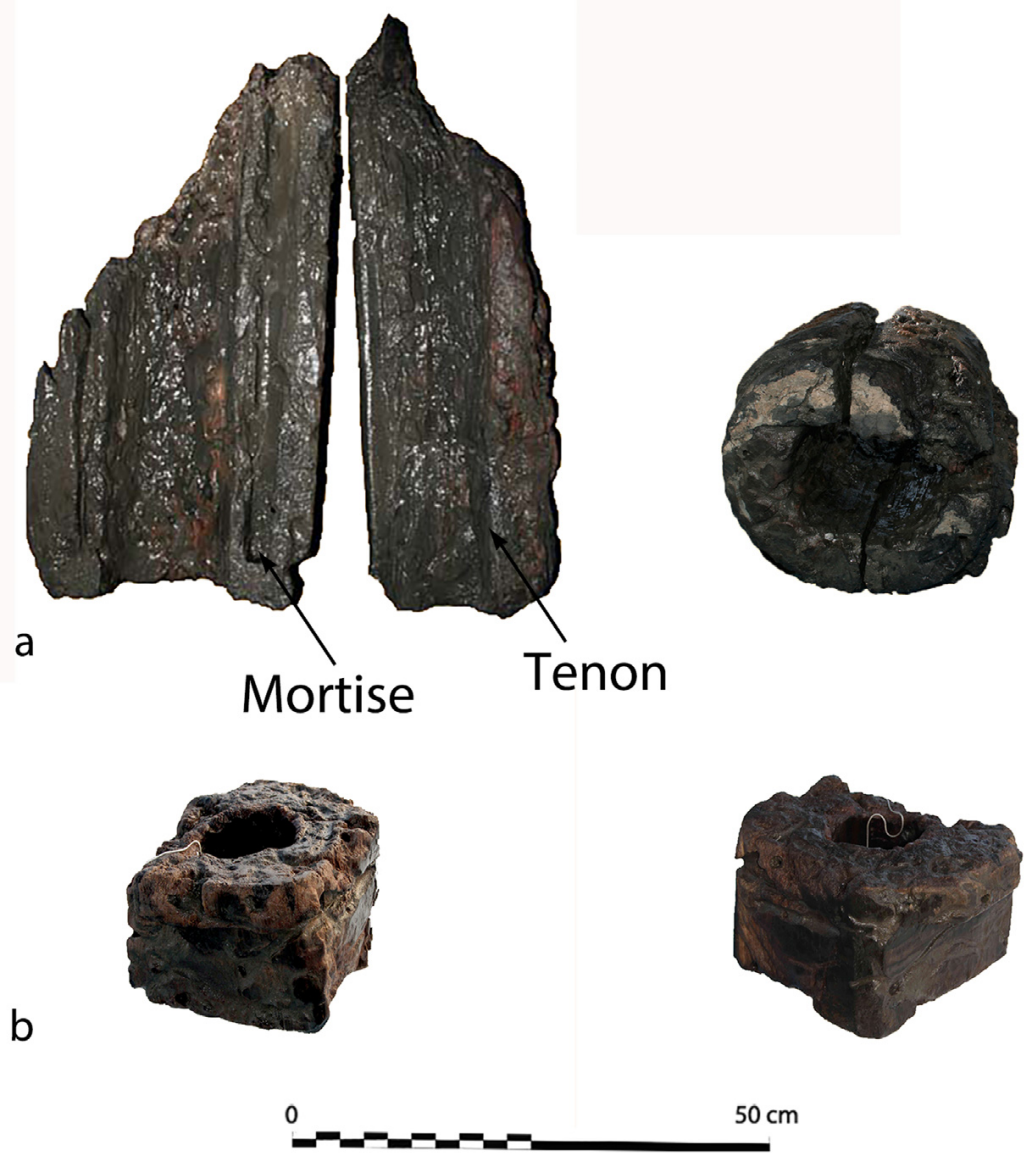
**a**. Pump-tube – **b**. Foot-valve. Photo Arnaud Cazenave de la Roche.

**The pump** ( Figure 16)

The pumping system was uncovered immediately after the mast step. It consisted of a pump-well formed by a quadrangular receptacle inside which were found the remains of a pump placed between two floor-timbers.

The pump-well was made up of four chestnut planks 3 cm thick and 30 cm wide. The pump was composed of a foot-valve and the remains of the lower part of a pump-tube. The foot-valve was a square single wood piece of wood with sides of 15 to 23 cm and a central hole of 10 cm in diameter. Its upper part was provided with a collar that rested on the floor-timbers (V21 and V22) between which it was inserted.

The pump-tube was connected to the pump-valve. It was found leaning towards the stern, and levelled in its upper part, leaving a maximum height of 62 cm. The tube was composed of three pieces of wood 7 cm thick, with a curved surface, assembled by a system of tenons and mortises located along the edges. The whole set of wood pieces was tied together with a thick rope, the marks of which could be seen on the outer side of the tube, whose corrugated surface was evidence of vigorous girdling.

The *Mortella III* pump has several characteristics that distinguish it from those used in the Atlantic nautical space. It contributes to documenting the Mediterranean traits, some of which can be traced back to the Mediterranean antiquity ( Bendig, 2020, 17). 

**Figure 17.**
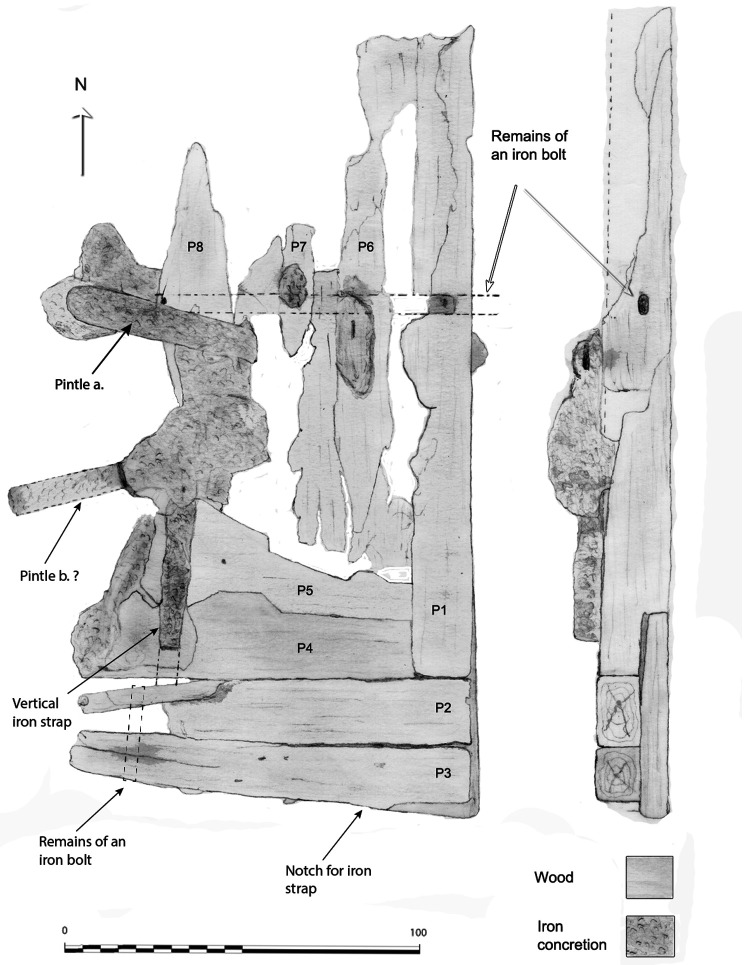
The rudder. Drawing Arnaud Cazenave de la Roche.

**The rudder** ( Figure 17)

The remains of the rudder were composed of a blade made up of nine degraded wooden pieces lying flat just after the skeg of the keel. Rudder remains have rarely been observed on wrecks of this period.

The blade consisted of four quadrangular pieces of wood with an average section of 20 cm arranged vertically. The arrangement of the ironwork remaining on the blade indicated that a fifth piece existed initially. The initial whole dimension of the blade was 1 metre wide and 20 cm thick. Its height was preserved over 2 metres. One of its specific features was the morphology of its lower part which was made of five pieces of wood arranged horizontally according to a particular assembly, with one of the pieces supporting the four others ( Figure 16). The weakness of this curious assembly raised the hypothesis that it could be a repair. 

The vertical wooden pieces were in too poor a condition to observe their fastening method. On the other hand, the horizontal pieces showed evidence of nailing with six round nails, each 11 to 13 mm in diameter. As for the reinforcement plank, it was fastened with five nails which increased the connection between the pieces P1 to P4. In addition to the nailing, a system of horizontal and vertical bolting reinforced the solidity of the whole piece.

As for the iron-work *,* two rudder pintles were observed, but they were too concretioned to observe their morphology in detail. Nevertheless, the flat irons of their port and starboard branches did not enclose the entire rudder. Their branches were interrupted before the end of the rudder's aft part, as is the case for the Villefranche-sur-mer wreck.

### 3.3 Shapes and proportions of the *Mortella III* ship

The architecture of the shapes and proportions of the *Mortella III* ship is the second aspect of our line of research on naval architecture. It raises essential questions related to its geometry. Two stages of the excavation have enabled us to make significant progress on this subject: the discovery of the fore end of the keel led to the precise measurement of its length and the reconstruction on land of the master-frame remains M27. Having discovered this information, it was possible to study the shape of the ship and assess its proportions.

It should be noted, however, that the remains of the wreck's frame burnt until the level of the first third of the second-futtock, i.e. at a height of only 2.10 metres vertical to the back of the keel. Therefore, it was necessary to make assumptions about the continuation of the transverse profile and the height of the decks, of which we have no remains. This study is presented in chapter 5 of the doctoral thesis devoted to the Mortella III wreck shipbuilding ( Cazenave de la Roche, 2018a and Cazenave de la Roche, 2018b). It is now being re-examined as part of the *Modernship* project (MSCA, Horizon 2020, GA 843337), which will allow it to be updated, clarified and corrected, particularly with regard to the assumptions about the distribution of the decks.

***3.3.1 Ship shapes*.** In the 'frame-first' carvel-building system, the shape of the main section is essential to restore the transverse profile of the ship. In the case of the *Mortella III* wreck, however, it remained difficult to restore due to the failure of the heads of the floor-timbers, which caused a collapse of the transverse framework. To overcome this difficulty, we opted to dismantle the M27 master-frame and study it on land. However, it was not certain that this would be successful, as two conditions had to be met for the restored form to be accurate: firstly, we had to ensure that the wood had not been deformed by being in the water, and secondly, that the scarfs of the frame elements, floor-timbers, first-futtock and second-futtock could be replaced with certainty in their original position. These conditions having been met, we now have a representation of the shape and dimensions of the lower part of the main section up to the second-futtock on which we can rely with confidence ( Langenegger, 2020b, 191–195). ^9^

**Figure 18.**
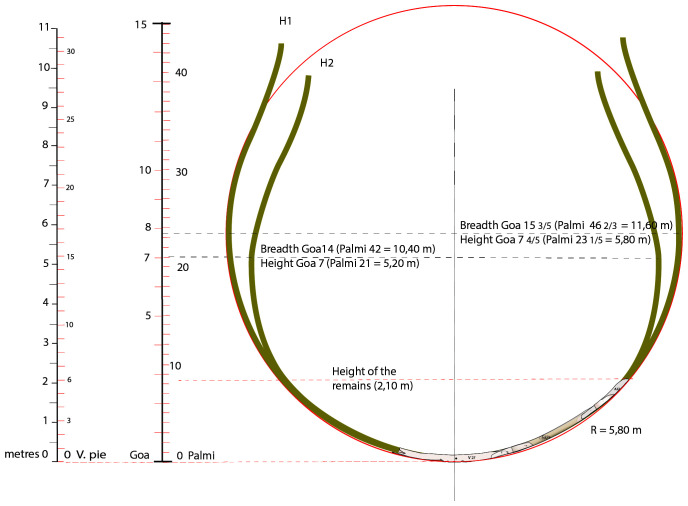
The two hypothesis of the transverse shape of the *Mortella III* nave (H1 and H2). Drawing A. Cazenave.

We can establish with certainty that we are dealing with a ship whose rising floor-timber results in a fairly high hull bottom, which contrasts with the flatness of ships of the Atlantic constructive tradition, such as that of Red Bay (1565), for example, and attests to a relatively high draught ( Figure 18).

The shape of the bottom of the *Mortella III* is very round: there is no inflection at the turn of the bilge on the floor-timbers and, as a result, the turn of the bilge is not marked. The frame follows an almost perfectly circular arc of 5.80 metres radius. In this respect, the design of the hull bottom is similar to that of the Villefranche-sur-mer wreck (1516). In the case of the *Mortella III* wreck, above the first half of the depth of hold, we do not know how this line evolves due to the lack of remains, but in the case of Villefranche-sur-mer, we observe that it follows a curve with a radius half the one below the first-half of the depth of hold ( Figure 18). This case is evidenced by archaeological remains but, to our knowledge, is not mentioned by any historical sources of the period. Therefore, in our opinion, there are two possibilities ( Figure 18). The first is that, above the first half of the depth of hold, the curve of the master-frame of the *Mortella III* wreck follows the same arc of a circle as advocated by the Iberian authors in the 16 ^th^ century ( Escalante De Mendoza, 1575; García De Palacio, 1587; Oliveira, 1580). In this case, maximum breadth would be 15 3/5 *goa*, i.e. 11.60 metres (Hypothesis 1). ^10^ The second is that above the first half of the depth of hold, the curve bends inwards on the model of the Villefranche-sur-mer wreck, in which case maximum breadth would be 14 *goa*, i.e. 10.40 metres (Hypothesis 2, H2). This hypothesis was initially favoured because of the chronological, geographical and technical-cultural proximity of the two wrecks.

**Table 2.** Linear and volume measure units for ships mentioned in the article.

ORIGIN	LINEAR UNITS		VOLUME UNITS	
**Venice**	*piè(di)* = 16 *dita*	0.3477 m	Venetian *botta*/ *botte* estimated weight occupied in hold 10 *staia*	c. 0.600 m **^3^** c. 640 kg 0.833 m **^3^**
	*passo geometrico* = 5 *piè(di)*	1.7385 m		
			Venetian *staio/ster*	0.0833 m **^3^**
**^Sicily^**			*salma generale*	0.2655 m **^3^**
**^Naples^**			*carro* = 36 *tomoli*	1.9915 m **^3^**
**^Genoa^**	*palmo di canna* = 12 *dita genovesi*	0.2478 m	*mina* = 4 *staja* 1310-1550 after 1550	0.1058 m **^3^** 0.1121 m **^3^**
	*gobito,chobitto, goa* = 3 *palmi*	0.7432 m	(foreign *botte v* **^3^**) estimated occupied in hold 2.5 *salme* corresp. weight 10 *cantari*	c.0.445 m **^3^** 0.6638 m **^3^** 476.5 kg

***3.3.2 The height of the decks*.** The arrangement of the decks is important for the organisation of the cargo and the determination of the tonnage of the ship. However, in the case of the *Mortella III* wreck because of the absence of remains, this cannot be determined with certainty and can only be the subject of hypotheses. The Italian texts of the 16 ^th^ century mention two ‘ *coverta*’ (or ‘ *coperta*’ or ‘ *choverta*’) and a ‘ *tolda*’ for the upper deck. Thus, the *nave* seems to be a ship traditionally provided with three decks. However, the Venetian and Ragusan authors of the 16 ^th^ century, such as Theodoro de Nicolò ( de Nicolò, 1550), Nicolò Sagri ( Sagri, 1571), as well as the anonym author of ‘Misure di navilii’ ^11^ mention a ‘ *tolda’* above the first two ‘ *coverte*’. Unfortunately, no Genoese treaty has survived to explain what the practice was in Genoa at that time. Curiously the Genoese and Ragusan contracts for the construction of *navi* coming from the notarial archives published by L. Gatti ( Gatti, 1975) only refer to the first and second ‘ *coverta’*. A third deck and the term ‘ *tolda’* are never mentioned. Nevertheless, it seems to us that the *navi* described was necessarily equipped with an upper deck above the ‘ *seconda coverta’*, given that the latter was generally located close to the waterline. The text of the Ragusan Nicolò Sagri, which explicitly mentions a ‘ *tolda*’ above the second deck, while the Ragusan notarial contracts make no reference to it, seems to support this interpretation.

As far as the *Mortella III* wreck is concerned, in our monograph we referred to an orlop and two decks. A more accurate description could be used, based on the coeval texts in Italian mentioning, as we said, two ‘ *coverte*’ and a ‘ *tolda*’. Nevertheless, it is likely that the largest *navi* were also equipped with an intermediate reinforcing structure between the keel and the first deck. It was composed of beams and a floor that was removable or partially removable and was called *falso ponte* in Italian, *faux-pont* or *entrepont* in French, *orlop* or *overlop* in English and *baos vacíos* (i.e. ‘empty beams’) in Spanish. Archaeology has revealed its presence on the *Lomellina* wreck, which is bigger than *Mortella III*, where the structures of an orlop were found 2.40 m above the keel, hanging on the first two clamps ( Guérout *et al*., 1989, 93 and 98).

The hypothesis proposed in our initial work followed the model given by the *Lomellina* wreck. Our first study suggested the presence of an orlop located at 2.40 m above the keel, a first deck located at 4.27 m and a second deck at 6.15 m. In the light of the analysis of the documents from the Genoese archives, and the texts of Theodoro de Nicolò and Nicolò Sagri ( Cazenave *et al*., in press), this hypothesis is currently being revised. An important fact to take into account is that in 16 ^th^ century Italian shipbuilding, the *seconda coverta* was generally located at or near the maximum breadth, where the waterline was also located. In the case of the *Mortella III nave*, this makes it possible to suppose the height of the *seconda coverta* at 7 *goa* (5.20 m) from the keel. This value was also that of the depth of hold ^12^ and, according to the rule of proportion in use at the time, the maximum breadth was twice the depth of hold, i.e. 14 *goa*.

If we apply to the *Mortella III* wreck the rules of proportion set out by Nicolò Sagri for his 30 *piè nave*, the *prima coverta* would be located at 3/5 of the second, i.e. *goa* 4 1/5 (3.15 m). As for the upper deck, it would be located above the keel at a height equivalent to 1 ½ times the depth of hold, i.e. 10 ½ *goa* (7.8 m). These values are also close to those advocated by Theodoro de Nicolò.

**Figure 19.**
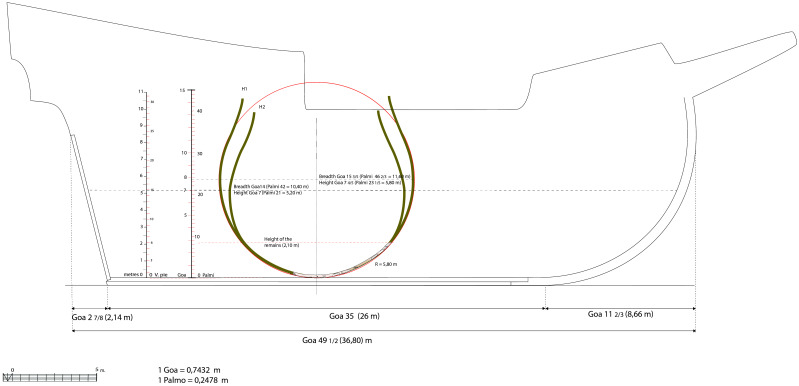
Hypothesis of the longitudinal shape of the *Mortella III* nave.

***3.3.3 The proportions of the ship ( Figure 19)*.** The proportions are determined by the relationship between the main dimensions of the ship. There are four of them:

- The maximum breadth, i.e. the greatest width of the ship.

- The length of the keel, with the notion of the keel bearing on land (which includes part of the heel of the keel and the stem-post).

- The length, with the notion of ‘ *roda a roda*’ (‘stempost to sternpost’) - length as measured at the level of the second deck.

- The depth of hold measured between keel and second deck (sometimes to the first deck).

As for the *Mortella III* ship, the length of the keel ‘bearing on land’ is estimated to be 26 metres (25 metres measured + 1 m estimated of the part of the stempost bearing on land). The length from ‘ *roda a roda*’ can be obtained by adding the keel length to the stempost and to the sternpost. In our study, the dimensions retained to restore these two pieces allow us to estimate the aft overhang at 2 7/8 *goa*, i.e. 2.14 m, and the forward overhang at 11 2/3 *goa*, i.e. 8.66 m (1/17 and 1/3 of the keel length, respectively). These two measurements added to the keel length allow us to calculate the length: 49 ½ *goa*, i.e. 36.80 m. Therefore, depending on the considered hypothesis of the width at maximum breadth (H1 or H2), we have the following two possibilities:

- *Hypothesis 1* (maximum breadth 15 3/5 *goa*, i.e. 11.60 m): the ‘maximum breadth to keel length’ ratio can be written: 15 3/5 : 35 => ratio = 1 : 2.24. We can calculate the ‘maximum breadth to *roda a roda* length’ ratio as 15 3/5 : 49 ½, i.e. 1 : 3.17. In summary, H1 ratio ‘breadth to length of keel to length from *roda a roda*’ is: 1 : 2.24 : 3.17

- *Hypothesis 2* (maximum breadth 14 *goa,* i.e. 10.40 m): the ‘maximum breadth to keel length’ ratio can be written as 14 : 35, i.e. 1 : 2.50. If we consider the length of 49 ½ *goa*, the ‘maximum breadth to head-to-head length’ can be written as 14 : 49 ½, i.e. 1 : 3.54. In summary, H2 ratio ‘breadth to length of keel to length from *roda a roda*’ is: 1 : 2.50 : 3.54

The ratio of hypothesis 1 (1 : 2.24 : 3.17) is almost identical to the one of the Calvi wreck (1 : 2.18 : 3.19). It is also close to (even if slightly higher than) the *'As-Dos-Tres*' rule ^13^ recommended by Iberian authors for their merchantmen and by Nicolò Sagri for his 30 *pie nave,* in the first two thirds and the last third of the 16 ^th^ century, respectively.

The ratio of hypothesis 2 (1 : 2.50 : 3.54) can be compared to that of the *nave quadra* of the Fabrica di galere (1 : 2.45 : 3.58) or to the *nave* of 700 *botte* of Zorzi Trombetta da Modon, although the latter has a slightly more stretched profile, mostly due to longer overhangs (1 : 2.56 : 3.80). From an archaeological point of view, these proportions are almost identical to those of the *Lomellina nave*, whose ratio is 1 : 2.56 : 3.52

## 4. Wood: Material and Dendrochronology

### 4.1 The material: species and condition of the wood

The xylology study of the timber components of the *Mortella III* hull is based on the analysis of 28 samples. The species determined for the majority of the elements is deciduous oak ( *Quercus sp*.). The elements of type keel (upper timber MA.01), floor-timber, first and second-futtocks, sister-keelsons of the mast-step and their fore key, clamp, and tumulus A planking come from the felling of sessile oaks ( *Quercus petraea*), as demonstrated by the anatomical structure of the cells and the particularly slow growth rate of these trees with an average of one millimetre per year at full maturity. This heliophilous species grows in well-drained soils, unlike the pedunculate oaks ( *Quercus robur*), which prefer more humid environments. The pedunculate oak grows faster in the summer months and therefore its wood is harder; specifically it has been used for the second-futtocks and tumulus B planking.

Other species were observed including alder ( *Alnus sp*.) used for lower keel timber (MA.2). In the open air, this wood is not very resistant because it reacts negatively to changes in temperature and humidity. As a result, it deteriorates within a few years. However, when completely immersed in water, it is rot-proof and will last for thousands of years. It was frequently used as foundation piles for prehistoric palafitte settlements. The most notable example is its use in the construction of part of the city of Venice, Italy ( Macchioni *et al.,* 2016). A third species, beech ( *Fagus sylvatica*), has been identified for certain elements of the ceiling. It is a hard, heavy and very resistant wood but it tends to split and is not very durable. Therefore it is mainly used indoors, protected from the weather.

Concerning the oak components, a study was carried out to investigate possible compression of the wood that would have changed the original dimensions of the cross-sections. This included a floor-timber, a first and second-futtock, a garboard and fore key. This diversity of timber components allows for a good representativeness to quantify possible deformations in the sections. The oaks used for these components come from two different stands. The first is made up of 120 year old oaks with a trunk diameter of between 20 and 24 cm. The second stand consists of oak trees of about 170 years of age with a trunk diameter of between 34 and 40 cm. Depending on the desired type of pieces, an oak log is selected and the intensity of shaping is adapted to obtain the desired section; 180 cm ^2^ for floor-timbers, first and second-futtocks, 111 cm ^2^ for the section of the garboard and 345 cm ^2^ for that of the fore key ( Table 3).

**Table 3.** Specifications of timbers of the framework under investigation for possible compression in the wood structure.

Timber	Species	Pith	Measured age (years)	Sapwood	Surface cm ^2^
**Garboard** ** 25/27**	*Quercus*	yes	52	no	111
**First-** **futtock** ** 27B**	*Quercus*	yes	41	no	175
**First-** **futtock** ** 27A**	*Quercus*	5	65	no	197
**Second-** **futtock ** **27**	*Quercus*	2	134	no	189
**Floor-** **timber ** **27**	*Quercus*	3	124	no	170
**Fore key**	*Quercus*	yes	116	yes	345

**Figure 20.**
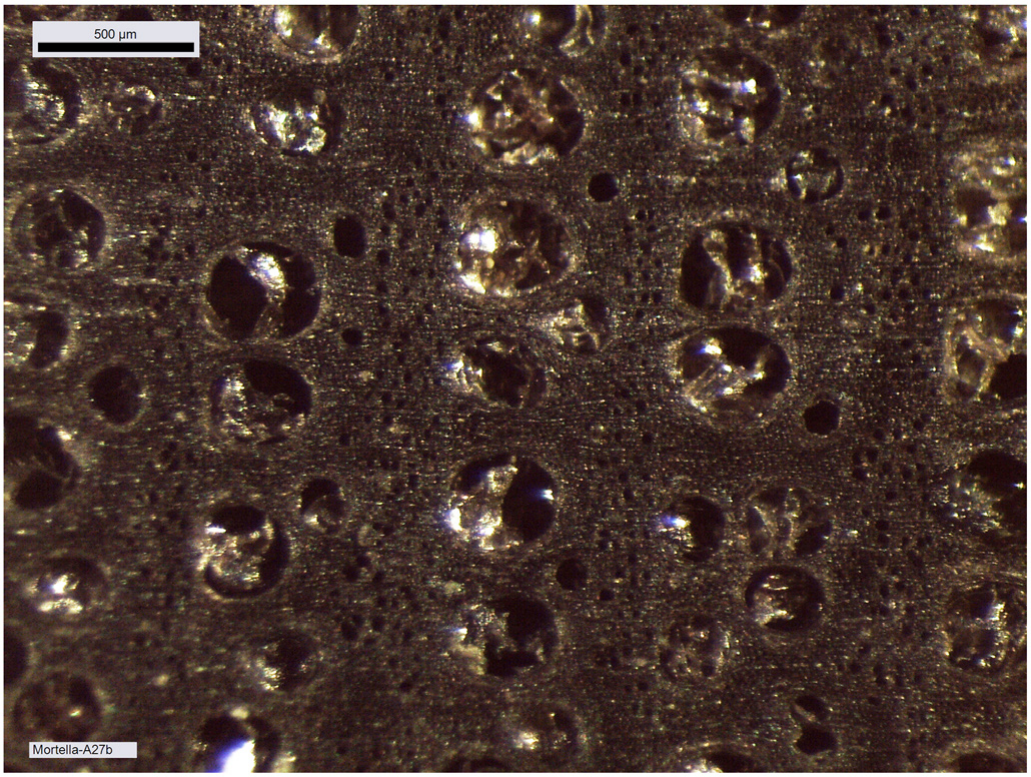
Macroscopic image of the growth rings of second-futtock A.27. The large cells of the initial wood have not undergone any transverse deformation and in the final wood the smaller pores are also clearly visible and show no compression. Photo: Fabien Langenegger.

An initial visual observation allows us to determine the state of preservation of the wood and to identify areas that have been degraded by bacteria and are susceptible to collapse. The strength of the wood in transverse compression varies according to the position of the annual layers. The observation is easier when the compression is in the direction of growth rather than laterally, in other words, when the compression is perpendicular to the grain of the wood. By observing the shape of the vessels and pores in the wood, it will be possible to determine whether transverse compression exists in the samples and to quantify it ( Figure 20).

**Figure 21.**
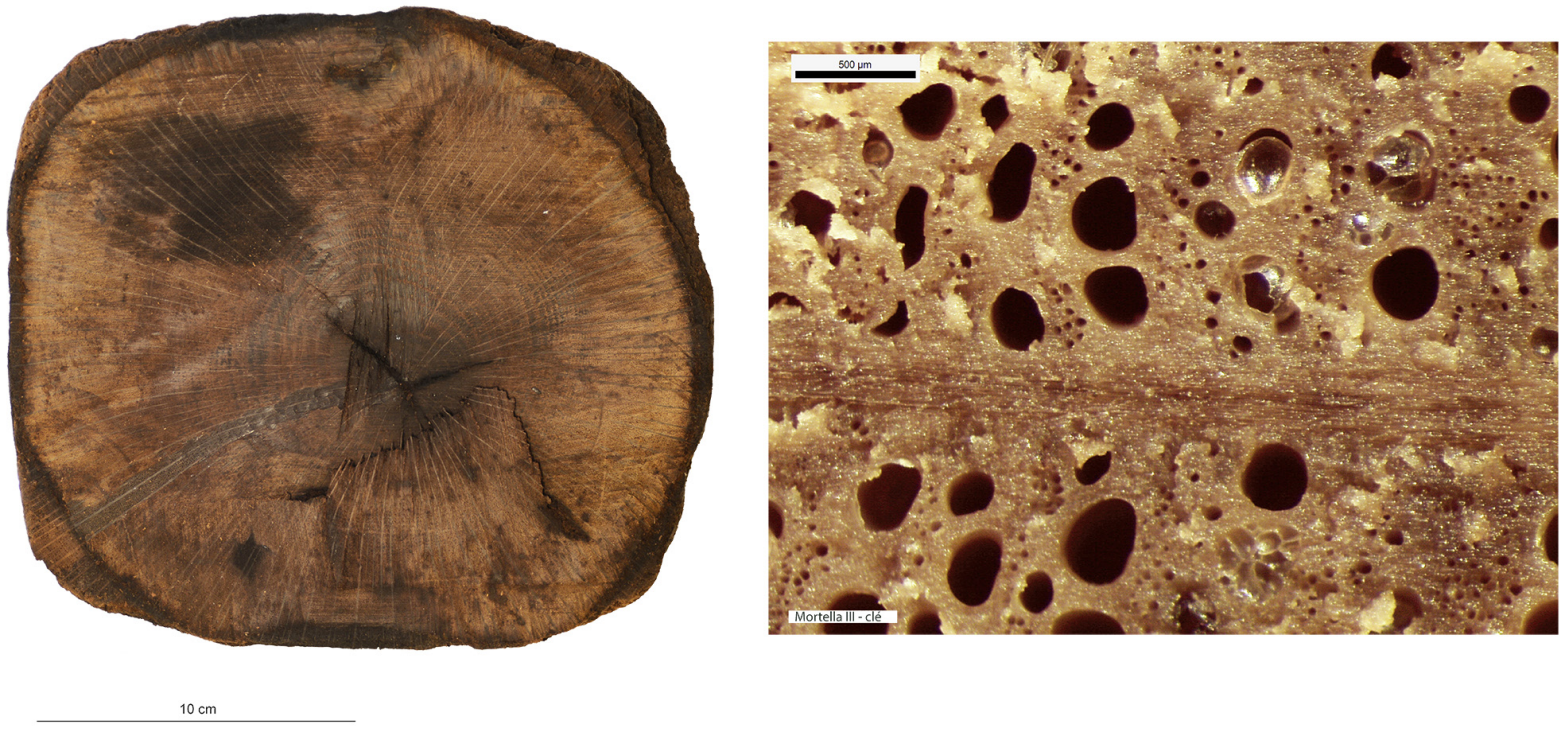
Fore key, photograph of the cross-section and macroscopic image of the sapwood cells. photos: Fabien Langenegger.

Cell compression was observed on the floor-timbers or first-futtocks, but this can be estimated to be a maximum of 1 mm and is concentrated only at the periphery of the section. The cross-section of the fore key still preserves much of the sapwood. The presence of the sapwood can certainly be explained by the need to obtain a very large cross-section for this architectural piece, measured on this sample at 345 cm ^2^. Livewood is much more fragile than heartwood and deforms more easily. However, the observed cell compression is very limited in the sapwood ( Figure 21). The reduction of the sample diameter due to weathering does not exceed 1 mm.

**Figure 22.**
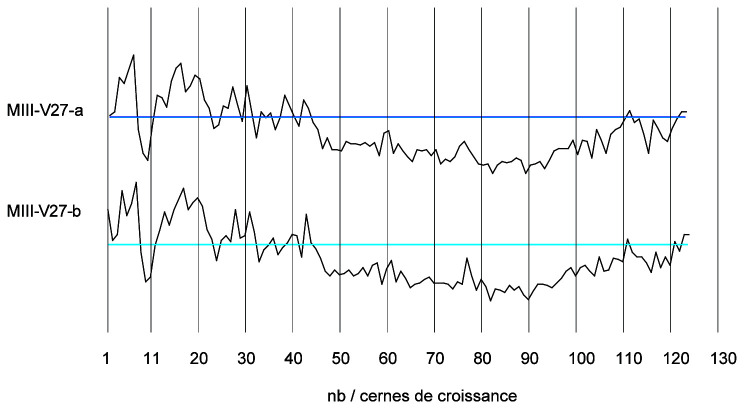
The curves measured on floor-timber V.27 are very regular with an average ring size of 0.88 mm. No compression of the wood is visible on this curve, and a resumption of growth is even observable at the periphery - CAD: Fabien Langenegger.

**Figure 23.**
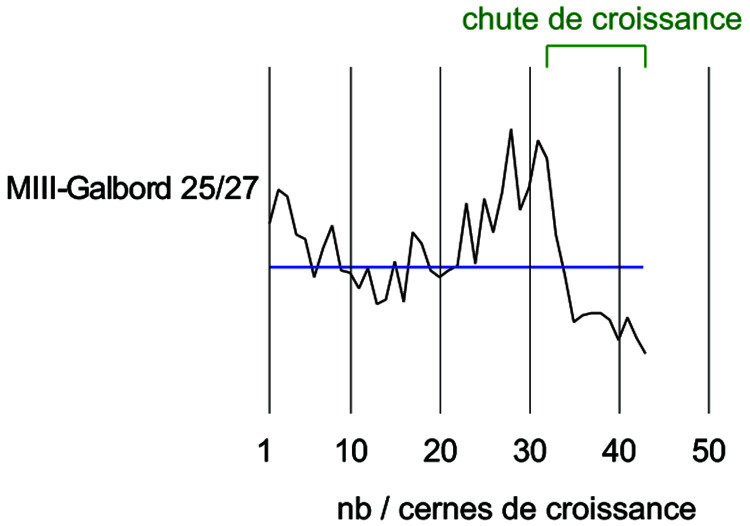
The garboard curve shows a drop in growth from the 32 ^nd^ ring onwards. This is due to a strong compression of the wood - CAD: Fabien Langenegger.

By studying the growth curves of the samples, a crushing of the wood fibres can also be identified with an abrupt decrease of the ring width in the final part of the curve. The trend of the growth curve of the second-futtock is very flat and does not show a growth drop due to compression of the section periphery ( Figure 22). The garboard shows the greatest deformation of the wood cells. It is observable over a width of 1 cm. The vessels of the original wood are completely crushed, but the difference to the original cross-section is limited and amounts to about 5 mm. The central part of the timber is not affected by this crushing and the cells are well preserved. This compression is clearly visible on the growth curve. The curve suddenly dips below 1 mm at the level of the crushing of the wood ( Figure 23).

The transverse compressions noticed on the sections are only due to the alteration of the wood's periphery. They can be observed on several samples, but the losses in relation to the original dimensions are limited to a few millimetres at most and do not modify the size of the sections sufficiently for these losses to be taken into account in the study of the architecture.

### 4.2 Dendrochronology: dating and origin of wood

Dendrochronology is an absolute dating method that analyses variations in the width of tree rings. It allows precise dating to the nearest season. In fact, by observing the composition of the cells of the last growth ring at the time of felling or natural death of the trees, the dendrochronologist can refine the dating between spring, summer or autumn/winter which corresponds to the period of vegetative halt of the tree.

This level of accuracy implies the presence of the cambium, the primary productive layer of the living plant between the last ring and the bark, on the sample. The accuracy of a dendrochronological dating can be classified into three levels. The optimal date is the one that defines the felling season of the tree. However, when the cambium is not present, but at least one sapwood ring is observed, an estimate of the felling date is made. The thickness of the sapwood (the living part of the tree) varies very little in the same stand of oaks and allows, when one or more rings of living wood are found in a sample, to estimate the felling date. On average, the number of rings in the sapwood varies between 20 and 30 for old oaks and includes about ten rings for young trees.

To be more precise, the sapwood is given the same number of rings as those counted in the last 2 cm of the heartwood. Then the felling date is placed in a chronological range between a minimum (or optimum) and a maximum estimated date. However, if the processing or the state of preservation of a sample does not allow the slightest trace of sapwood to be discerned, an estimate of the year of felling becomes impossible. The number of rings missing in the heartwood up to the departure of the sapwood cannot be determined. To get close to the date, 20 years are systematically added to the old wood, 10 to the young wood (which correspond to the years of sapwood that are missing) in order to obtain a *terminus post quem*. The tree cannot have been felled before this date. To determine the construction period of a ship, the accuracy of the dendrochronological date must be considered and only those with sapwood can have the date determined.

**Figure 24.**
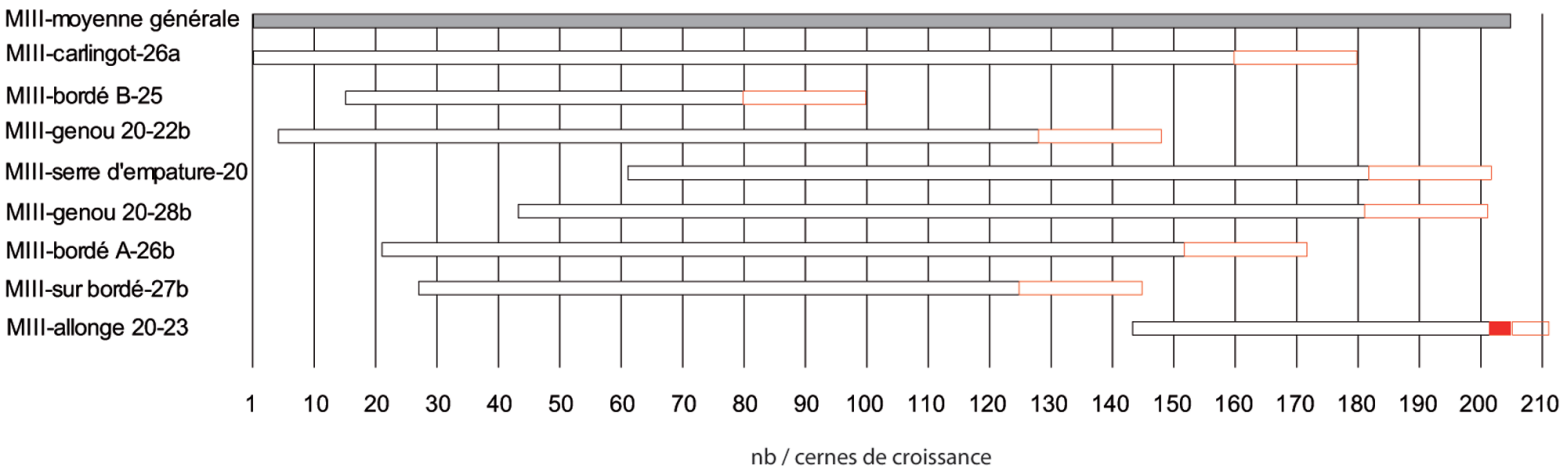
Block diagram of the eight framework components in relative dating - Fabien Langenegger.

About 40 measurements, totalling nearly 3500 rings, were carried out on the samples to obtain the most representative average curves possible for each timber component. The trend of the growth curves is very different from one sample to another and shows a very heterogeneous origin of the timber. These averages were then synchronised together to obtain a relative dating between these different timbers ( Figure 24). Intensive shaping has removed all the sapwood, except for the average of the second-futtock 20-23BD. Younger wood, and therefore a smaller trunk diameter was used for this part of the ship and the maximum amount of raw material was kept to obtain a sufficiently large section. These few rings of ‘living wood’ are essential for estimating the date of the beginning of the construction of the hull. For the other samples, the heartwood was split to obtain the desired cross-sections. However, the importance of this shaping must be put into perspective by taking into account that with an annual growth rate of 1 mm, a removal of 4 cm in thickness represents 40 years of growth for these sessile oaks. The general curve, obtained from the synchronisation of these eight averages, is 206 years long and despite the heterogeneity of the growth of the oaks, it remains very representative of the different samples.

In order to obtain an absolute dating, the different averages obtained for *Mortella III* were tested on the numerous references available at the dendrochronology laboratory of Neuchâtel for the Northern Alps. The results show good indices with the central area of France and in particular Burgundy. The sister-keelsons, the first-futtock n°20 (n°28b), the clamp n°4, as well as the general average, give concordant results on several reference curves. The coefficients are around 4 for the Eckstein test and between 5 and 7.3 for the Student T test (BP). The visual synchronisation of the curves on the screen is very good for the sample taken from the sister-keelsons and allows a good absolute dating for the general curve and thus for all the samples studied. The construction date can be defined by the second-futtock n ^o^. 20 and these four sapwood rings. The growth width of the last rings of this section is particularly high and reaches 4 to 5 mm. Thus, the preserved sapwood width is already 2.14 cm. A normal width can be 3 to 3.5 cm and thus 3 sapwood rings would be missing until the cambium. An estimate of three to six missing rings was noted for this sample. Therefore, the earliest possible start of construction was between 1517 and 1520 ( Table 4). For the positioning of the other framework components, if a timber has no sapwood, as for old oaks, 20 rings are added systematically which gives a *terminus post quem*. The further away this is from the probable date of construction of the ship, the more intensive the processing on this timber has been ( Figure 24), unless it is reclaimed wood, which is unlikely for such a construction.

**Table 4.** Absolute dating of some samples from *Mortella III*.

Samples	Origin (AD)	Term (AD)	Estimation of the felling date	Number of rings measured
Sister-keelson (n°MIII carlingot- 26a)	1309	1469	> 1489	161
Plank (n°MIII bordé A)	1330	1461	> 1481	132
Plank (n° MIII bordé B)	1324	1389	> 1409	66
First-futtock (n°MIII genou 20-22b)	1313	1437	> 1457	125
First-futtock (n°MIII genou 20-28b)	1352	1490	> 1510	139
Clamp (n°MIII serre d'empature 20)	1370	1491	> 1511	122
Second-futtock (n°MIII allonge n°20-23)	1452	1514	**1517 à ** **1520 ap. ** **J.-C.**	63
Plank (n°MIII pièce sur bordé 27b)	1336	1434	> 1454	99

## 5. The line of research on material culture

### 5.1 The artillery of the *Mortella III* wreck

The study of the artillery of the *Mortella III* wreck is not easy, as no pieces were removed and brought to the surface. Their documentation is therefore based solely on observations, measurements and photographs (albeit of excellent quality), taken *in situ*. The thick layer of concretion covering the wrought iron presents another obstacle to their study. The information gathered is therefore both incomplete and not very precise. However, the experience gained during the excavation of the *Lomellina* wreck (1516, Villefranche-sur-mer, France), which contained an equivalent number of wrought iron artillery pieces ( Guérout *et al.*, 1989), showed that a careful examination of the morphology of the concretions allowed several characteristics of the pieces to be read.

**Figure 25.**
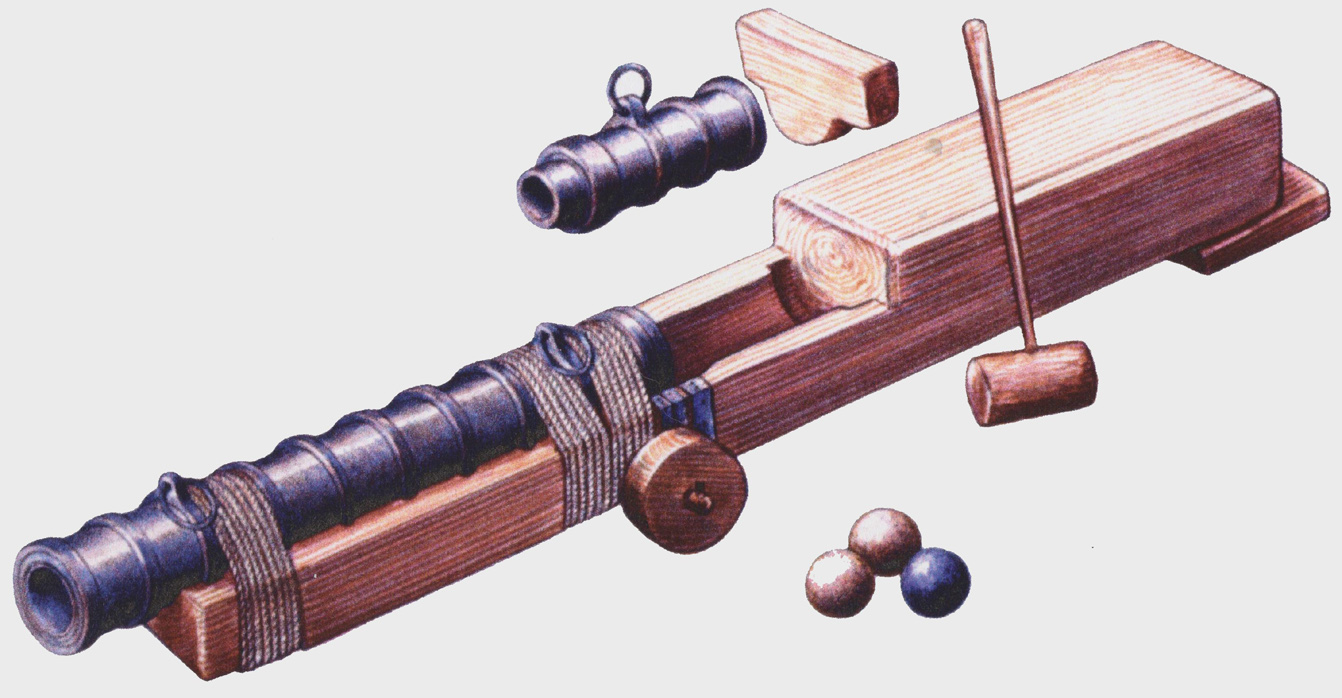
A *bombarda* of the *Lomellina* on its carriage. Noël Blotti.

In a first analysis, the artillery artefacts shown on the site plan ( Figure 1) are either barrels or removal breeches, both parts of wrought iron artillery pieces of the 16 ^th^ century called *bombarda* by the Italians, *bombarde* by the French, *lombarda* by the Spanish and ‘port piece’ by the English ( Figure 25).

***5.1.1 General characteristics of wrought iron artillery pieces***

**Principle of construction**

This wrought iron piece includes a barrel ( *tromba* in Italian, *caña* in Spanish, ‘hall’ or ‘barrel’ in English), a removable breech ( *canono* in Latin, *mascolo* in Italian, *recamara* in Spanish, ‘chamber’ in English) and a wooden carriage ( *fusto* in Italian, *affût* in French, ‘stock’ in English). The stock is sometimes fitted with wheels whose diameter is adapted to the firing site. It should be noted that the English term ‘bombard’, which is found in particular in the inventories of the Tower of London, refers to a piece of large calibre used exclusively on land.

**The barrel**

The barrel is a cylindrical tube open at both ends. It is built on the model of a cask: the internal part is formed of longitudinal iron bars (staves) of trapezoidal sections assembled by forging them around a wooden mandrel, while the external part is formed of sleeves hot-fitted on the staves. The latter are usually bent at their ends to hold the sleeves. Some narrower sleeves, called rings, are larger in diameter and protrude around the tube. These rings not only increase the strength of the barrel, but also allow it to be fixed to its stock. Some of these rings, usually two, have an extension at the top which allows one or two handling rings to be attached. Another manufacturing technique is sometimes used: the longitudinal staves are then replaced by iron sheets (two or three depending on the case) shaped into a tube on a mandrel and then covered with hoops as in the previous case.

**The removable breech**

Breeches, intended to contain powder, could be manufactured using several methods. According to F. Howard ( Howard, 1986, 442), who examined in detail the pieces found at Anholt in Denmark and exhibited in the Tojhusmuseet, they could be either forged from a block of iron shaped in the form of a cylinder closed at one end or made out of cast iron. A third possibility was to build them with longitudinal staves like the barrels, the rear end being then closed by an iron wedge, a plug introduced by force after being heated, as shown by the cutting of a removable breech from the Molasses Reef wreck (1510/1530) ( Simmons, 1988).

**The stock**

It is made of wood (oak or elm) and reinforced with ironwork. It is generally a single piece but the rear part can be arranged to form two separate longitudinal parts. The rear part is higher and serves as a stop for the removable breech. A wooden or iron wedge, inserted between the rear and upper part of the stock, holds the breech in place. The forward part is hollowed out with semi-circular grooves in which the rings of the tube are embedded. The stock can be equipped with wheels fixed on a wooden axle. The diameter of the wheels is adapted to the fire position, as the observations made on the *Mary Rose* wreck clearly show ( Rule, 1982, 104), as does the variety of wheels found on the *Lomellina* wreck ( Guérout, 2017). Aiming can be done either with a wedge inserted under the stock or with a wooden rack passing vertically through the rear part of the stock.

***5.1.2 Description of the artillery on the *Mortella III* site ( Table 5)*.** Nine barrels measuring between 1.80 metres and 2 metres in length (numbered Cn 1 to Cn 9) and five removable breeches measuring between 70 and 80 cm in length (numbered Cl 1 to Cl 8) are scattered on the site in three sets, two at the stern area on either side of the keel, and one at the bow of the wreck ( Figure 1). The concretions surrounding the pieces only allow an approximate description of their morphology.

**Figure 27.**
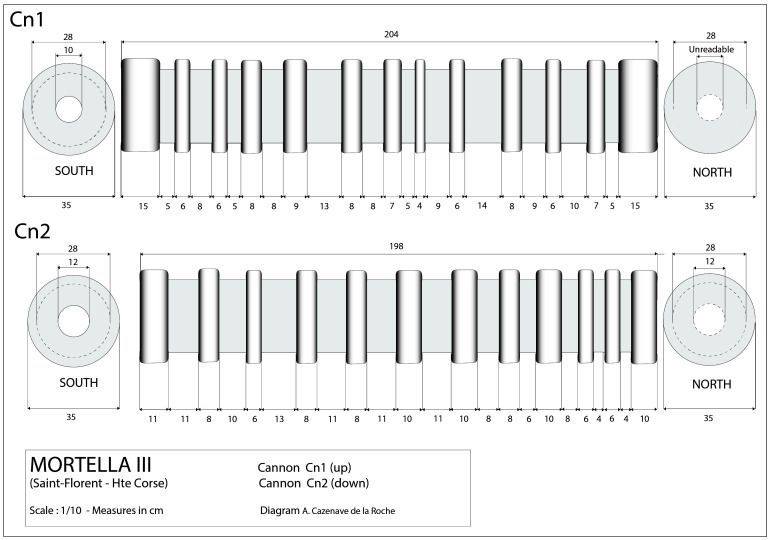
Measurements of Cannons Cn.1 and Cn.2 with concretions.

**Figure 28.**
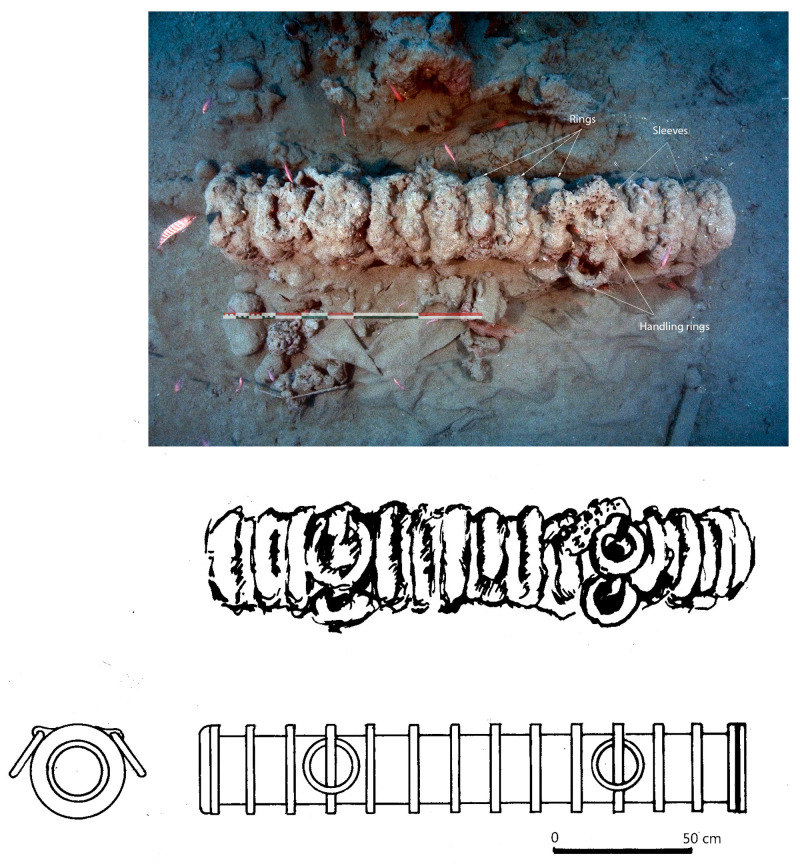
Cannon Cn3. Hypothesis of reconstruction – Photo Drawings Max Guérout. Christoph Gerigk.

**Stern port group** ( Figure 26, Figure 27 and Figure 28)

**Figure 26.** Set of cannons at stern: Cn.1-2-3 and Cl1-2 – © All rights reserved, Christoph Gerigk.

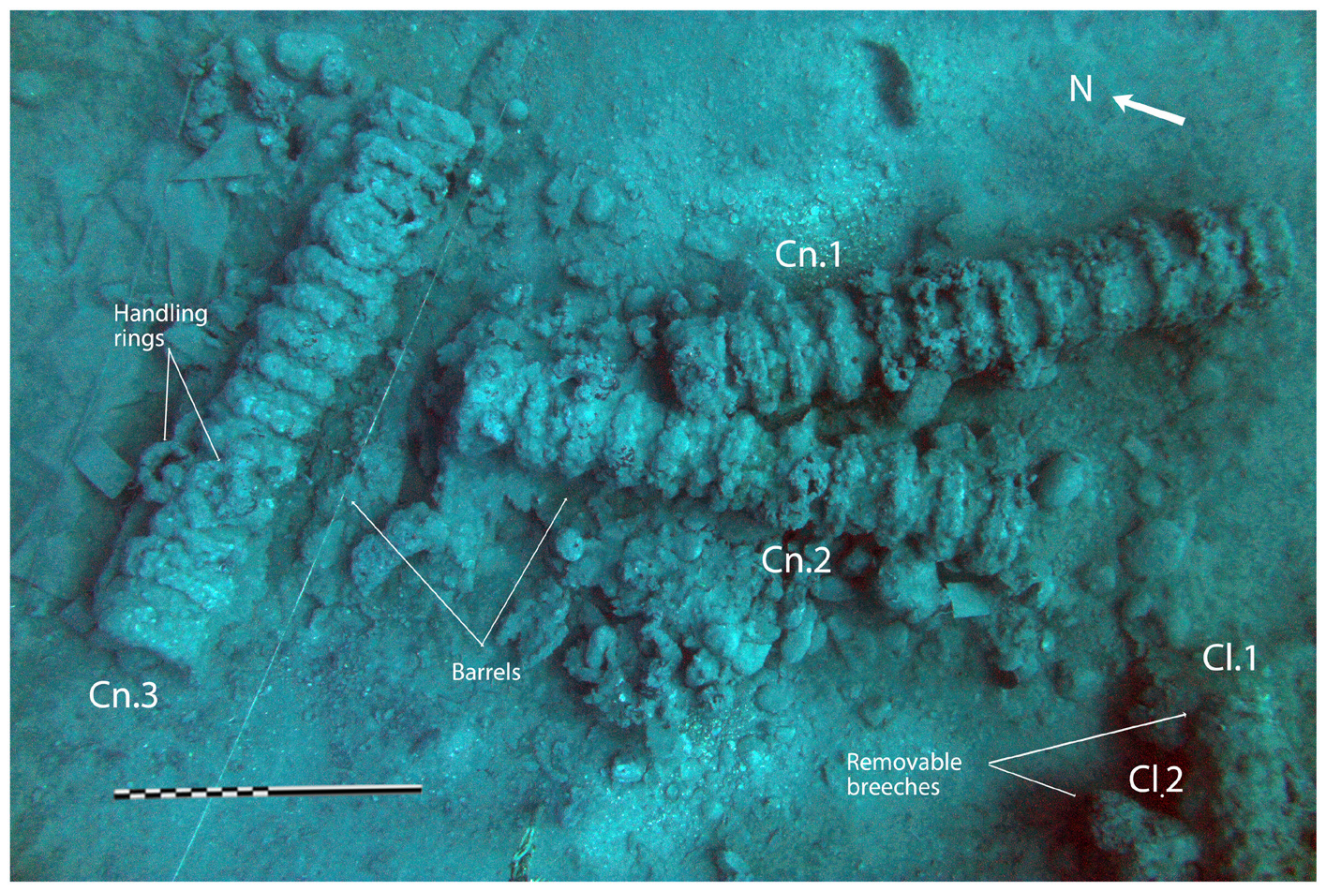

This group of components was documented on the sea bottom and is composed of four barrels and three removable breeches. The rings and handling rings on Cn 3 and Cl 3 are clearly visible.

*Barrel Cn 1*: The barrel is 204 cm long. There are 11 intermediate rings and 13 sleeves. ^14^ The sleeves have an external diameter of about 28 cm, the ribs a diameter of about 35 cm. The diameter of the muzzle opening is 10 cm; due to the presence of a calcareous concretion inside the tube, this measurement does not represent the calibre of the piece. The two ends of the barrel seem to be reinforced with two adjoining rings.

*Barrel Cn 2*: The barrel measures 198 cm, with 10 rings and 11 intermediate sleeves. The diameter of the muzzle is 12 cm. The outer diameter is 28 cm.

*Barrel Cn 3* ( Figure 28): The length is close to 200 cm, the outer diameter of the rings is 35–36 cm; the outer diameter of the sleeves is 27–28 cm. The 12 rings are about 6 cm thick and 15 cm apart. If we estimate the average thickness of the concretion at 1.5 cm, we obtain a width of the ribs of 3 cm and consequently a width of the sleeves of 12 cm, compatible with the measurements taken from the guns found on *Lomellina* wreck.

The two muzzles seem to be reinforced by two joined rings. The handling rings, with an external diameter of 24 cm, are attached to the third ring from the two muzzles.

*Barrel Cn 4*: The length of the barrel is approximately 205 cm. It is not possible to count the sleeves and rings.

*Removable breeches*: Cl 1, Cl 2 and Cl 3 are 80, 64 and 70 cm long respectively. Cl 3 is provided with a handling ring of 28 cm outer diameter. 

**Stern starboard group**

This set contains three barrels of the same type as those on port-side and a removable breech.

*Barrel Cn 5*: 190 cm long. The number of rings cannot be counted with certainty.

*Barrel Cn 6*: 200 cm long, with 13 rings,

*Barrel Cn 7*: 188 cm long, with 13 rings.

*Removable breech Cl 4*: 60 cm long.

**5.1.2.3 Stem area group** ( Figure 29)

**Figure 29.** Cannon set in the stem area – © All rights reserved, Christoph Gerigk.

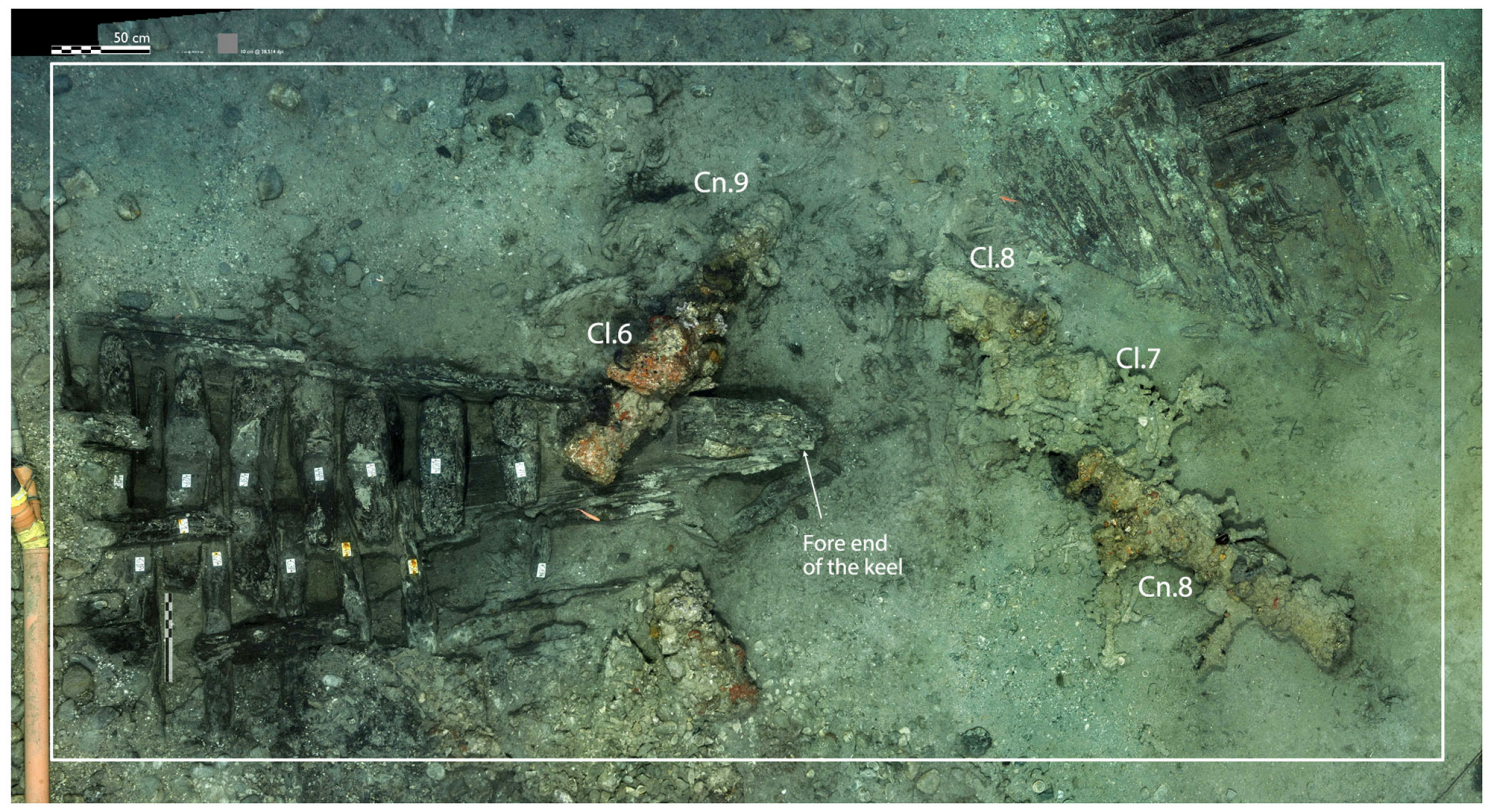

This group of components was entirely buried in the sediment and very degraded, probably due to electrolysis phenomenon.

*Barrel Cn 8*: 185 cm long. It is not possible to count the number of rings, which is more than 10.

*Barrel Cn 9*: 165 cm long, it is not possible to count the number of rings, because the removable breech Cl 6 rests on the barrel. The outside diameter is 23 cm; about 10 cm smaller than the other pieces of the stem are.

*Removable breeches*: Cl 5, Cl 6, Cl 7 and Cl 8 are 80, 60, 60 and 75 cm long respectively.

The number of removable breeches observed insofar seems low, as each port-piece was generally provided with at least two breeches, as can be found for example in the inventory of the *Louyse* (1516) ^15^ or in those mentioned by E. de Crouy-Chanel ( Crouy-Chanel, 2020, 416).

**Table 5.** Summary of artillery components.

N° Invent.	Components	Length in cm	Number of rings	Handle rings
Cn 1	Barrel	204	11	
Cn 2	Barrel	198	10	
Cn 3	Barrel	200	12	4 handle rings. Outer Ø 24 cm
Cn 4	Barrel	205	12 (?)	
Cn 5	Barrel	190	?	
Cn 6	Barrel	200	13	
Cn 7	Barrel	188	13	
Cn 8	Barrel	185	> 10	
Cn 9	Barrel	165		1 handle rings. Outer Ø 26 cm
Cl 1	Breech	80		
Cl 2	Breech	64		
Cl 3	Breech	70		1 handle rings. Outer Ø 28 cm
Cl 4	Breech	60		
Cl 5	Breech	80		
Cl 6	Breech	60		
Cl 7	Breech	75		
Cl 8	Breech	75

***5.1.3 The shot*.** The particularity of the shot discovered is that they are all made of stone. They can be divided into three categories: intact, cracked or fragmented and rough draft shot. Blocks of stone probably used to make shot have also been found. The shot provides accurate information for assessing the calibre of the pieces present at the site.

The small number of shot found is difficult to explain, even if the ship was involved in a battle before it sank. In fact, the number of shot on board is at least eight per piece, as is the case with the *Louyse*, whose inventory previously mentioned that for ten large iron cannons, possibly port-pieces, there were 80 ‘ *boulletz de pierre de grelz*’ (‘sandstone shot’) at the time of the death of Admiral de Graville. This number even reached 40 per piece during the armament of the Duke of Burgundy at l'Ecluse in May 1470, and 100 per piece on board the *Grande Maîtresse* in 1526 ( Guérout & Liou, 2001).

It is also worth mentioning the absence of iron and lead shots, which is also difficult to explain, particularly in the case of lead shots, as there was a lot of small-calibre artillery on board ships at the time.

**The complete shot**

The following were found:

- One 96 mm diameter shot

- 14 shot, diameter between 120 and 125 mm and weight of approximately 2.5 kg

- One 158 mm diameter shot with a weight of 5.7 kg

- Eight shot, diameter between 220 and 230 mm, weight between 16.4 and 17.1 kg. ^16^

The barrels Cn1, Cn2, Cn3, Cn4 and Cn6 can be compared to one of the barrels of the *Lomellina* (Cn13), which has undergone a long conservation treatment. It is almost 200 cm long. When the barrel is cleaned of concretions, the diameter of the rings is about 330 mm and the diameter of the sleeves 300 mm. The measured calibre is 230 mm.

As for the two shot with a diameter between 96 mm and 100 mm, they correspond to a barrel of a calibre of 10 to 11 cm, undoubtedly closer to the *passavolante* than to the *bombarda*. According to Luis Collado, the windage (difference between the diameter of the shot and the calibre of the piece) for a given shot is equal to its diameter multiplied by π/3, i.e. 1.047 ( Collado, 1586). Therefore, for a diameter of 220 mm the corresponding calibre is 230.3 mm.

**The fragmented shot**

The fragmentation of shot is possibly related to the fire that seems to have preceded the sinking of the ship. The presence of fragments of shot could also be interpreted as manufacturing failures. In this respect, three broken middle shots were found on the wreck of the *Lomellina*, a ship that did not burn but capsized at anchor.

**5.1.3.3 Rough draft shot**

Several rough draft shot were also found on the wreck of the *Lomellina*. The shaping of shot on board ships is attested by several documents of the time.

**A shot bearing an inscription** ( Figure 30).

**Figure 30.** Shot MIII-10-006 – © All rights reserved, Christoph Gerigk.

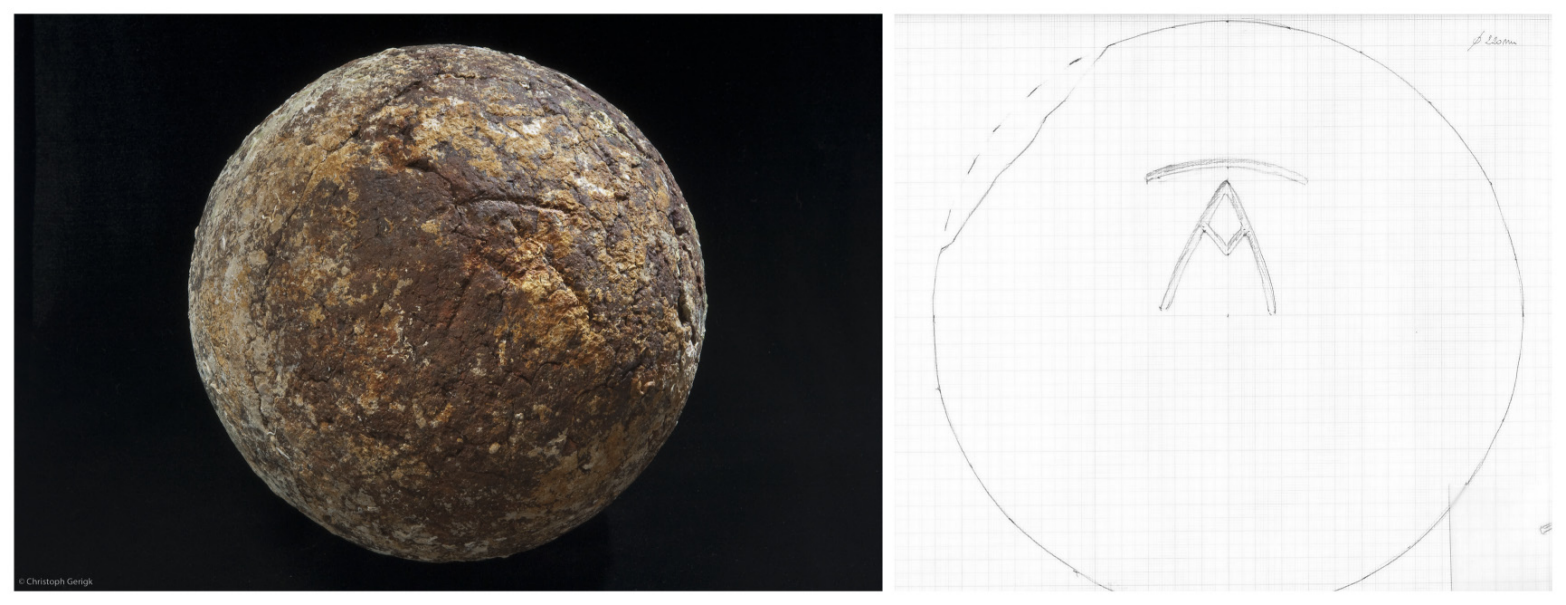

One of the shot bears an inscription, an A surmounted by a bar: Ā, a craftsman's mark frequently found in the Middle Ages and the Renaissance, but which is to our knowledge the first found on a stone shot. 

***5.1.4 Historical and archaeological references*.** In the Mediterranean, the works of Renato Gianni Ridella ( Ridella, 2011) and Furio Ciciliot ( Ciciliot, 2011) allow us to better understand this type of artillery built and used by the Genoese. On her side, the *Lomellina* wreck also provides archaeological references.

The Genoese wrought iron artillery has two main types: the *bombarda* and the *passavolante*, the latter being characterised by a long tube and a calibre of around 10 cm. The weights of the different types of Genoese *bombardas* quoted by Ridella are:

- Light *bombarda* of three *cantari* or 140 kg

- Medium *bombarda* of five *cantari* or 240 kg

- Heavy *bombarda* of seven *cantari* or 334 kg

- Heavy *bombarda* of 9.5 *cantari* or 452 kg

For the Genoese *bombarda* we have an archaeological reference with the barrels ( *trombe*) of the *Lomellina*. An attempt has been made to reconstruct the weight of the A.51 barrel, which was manufactured using the rolled and shrunken sheet metal method. It is 158 cm long with a calibre of 18 cm and an external diameter of the sleeves of 26 cm, and an external diameter of the rings of 32 cm. For an iron density of 7.32 we obtain a weight of about 266 kg close to that of the medium-size Genoese *bombarda*.

The largest barrels of the *Mortella III* are about 2 m long, so we can proportionally estimate that their weight is about 266 x 2 /1,58 or 336 kg close to the weight of the heavy Genoese *bombarda*. Those measuring 1.86 m weigh 284 kg by the same calculation.

In England the work of A. Caruana ( Caruana, 1984) and the pieces found on the wreck of the *Mary Rose* ( Rule & Hildred, 1984) complete our knowledge ( Table 6). The inventory of the Tower of London ( Blackmore, 1976) dated 1589 lists the weight of the barrels (halls) of the port pieces embarked on the *Hope*, they weigh respectively: 361.3 kg, 383.5 kg, 394.9 kg and 415 kg.

**Table 6.** Characteristics of the four port-pieces found on the *Mary Rose* wreck.

N° Inventory	Barrel length (cm)	Breech length (cm)	Calibre (cm)	Ø Shot (cm)
1979	130.8	57	20.32	18.37
MR81 3001	230	53	17	12 ( *in situ*)
MR81 2650	235	64	17	15
MR81 2604	245	63	17	15

The diameter of shot is either estimated, or reported to the shot found *in situ*, and shows a significant windage close to 1/10 of the calibre which differs from the method of Luis Collado mentioned above, which is about 1/20.

For Spain, the excavations carried out in the Caribbean in Highborne Kay ( Keith *et al*., 1984), Molasses Reef ( Oertling, 1989a) and Padre Island ( Arnold & Weddle, 1978) bring us some clarifications, although the artillery embarked on the Spanish *Flotas* seems instead to be attached to the type called in Spanish, *lombarda*, closer to the Genoese *passavolante* than to the *bombarda*.

**Chronology of wrought iron port-pieces**

In 1498 the decision was taken to supplement the armament of the Genoese merchantmen with bronze pieces; previously the ships were exclusively equipped with wrought iron artillery pieces.

However, it was from 1490 onwards that heavy port-pieces appeared on board merchant ships and even more so on-board ships armed for war. ^17^ To our knowledge, the latest Genoese inventory mentioning wrought iron port-pieces dates to 1579. It is that of *La Trinita* with a tonnage of 3500 *salme*, which in addition to 13 bronze cannons had one cast iron cannon and two wrought iron port-pieces. This is the moment when the baton is passed from wrought to cast iron. In the inventories of the Tower of London the first cannon made out of cast iron is mentioned in 1559. The last inventory to mention 15 wrought iron pieces is dated 1595.

Although covering almost the whole of the 16 ^th^ century, the period of use of wrought iron port-pieces is more likely to be found in the first half of the century, particularly for war ships which were the first to be equipped with the new weapons, while the use of port-pieces continued on board merchant ships, but sometimes also on certain ships armed for war, even though they had become obsolete.

***5.1.5 Discussion*.** Although it is difficult to draw general conclusions concerning the artillery of the ship from the pieces present on the site, we can however, with caution, put forward some hypotheses.

–Firstly, taking into account the tonnage of the *nave*, the number of pieces present on the site is below the standard of the time. In 1493, before the Genoese reform, the artillery of the *Fornara* included 30 wrought iron pieces, eight light stone guns and eight heavy stone guns in wrought iron ( Ridella, 2011, 40–41). From 1498 onwards, the Genoese merchant ships were equipped with bronze artillery; in his article, R.G. Ridella quotes the armament of several of them, as follows:

- In 1540, Ambrogio Doria's *nave* had two bronze half-cannons, 2 bronze sakers, 2 bronze falcons, 14 iron port-pieces, 12 swivels

- In 1544, the *nave Ruisecha,* named after her owner Leonardo Ruisecco, had two bronze pieces weighing 30 *cantari*, two bronze pieces weighing 22 *cantari*, two sakers weighing 12 *cantari*, eight iron port-pieces and eight iron swivels.

- In the same year, the *nave Spinola*, named after her owner Luigi Spinola, was armed with 2 bronze pieces of 35 *cantari,* 10 iron port-pieces, 3 iron *passavolanti* and 33 iron swivels

In England, at the same time, the Anthony Roll of 1546 lists the artillery provided for royal ships. If we consider a ship of medium tonnage, such as the *Pauncey*, 450 tons, built in 1543, her artillery includes: 13 bronze pieces (4 half-cannons, 2 culverins, 3 half-culverins, 4 sakers); 16 large iron pieces (12 port-pieces, 4 half-slings) and 67 pieces of small calibre ( Nelson, 2001: 45).

–Secondly, it is possible that bronze artillery pieces were recovered at the time of the sinking or later, ^18^ explaining why only wrought iron port-pieces are present on the site. It is indeed noteworthy that neither iron nor lead shot were found.

–Finally the distribution of the artillery at the two ends of the wreck, if it is not the consequence of the disorders resulting from the shipwreck, is uncommon. If it reflects the distribution of the artillery on board the ship in working order, it indicates artillery located in the fore castle and in a larger amount in the aft castle. The absence of pieces in battery in the waist is problematic and contrary to the recommendations of Antoine de Conflans ( Jal, 1842, 34–58) and Philippe de Clèves ( de Clèves, 1558).

### 5.2 The anchors of the *Mortella III* wreck ( Figure 31 to Figure 34)

**Figure 31.** Anchor F-MIII-AW of the *Mortella III* wreck – © All rights reserved, Christoph Gerigk.

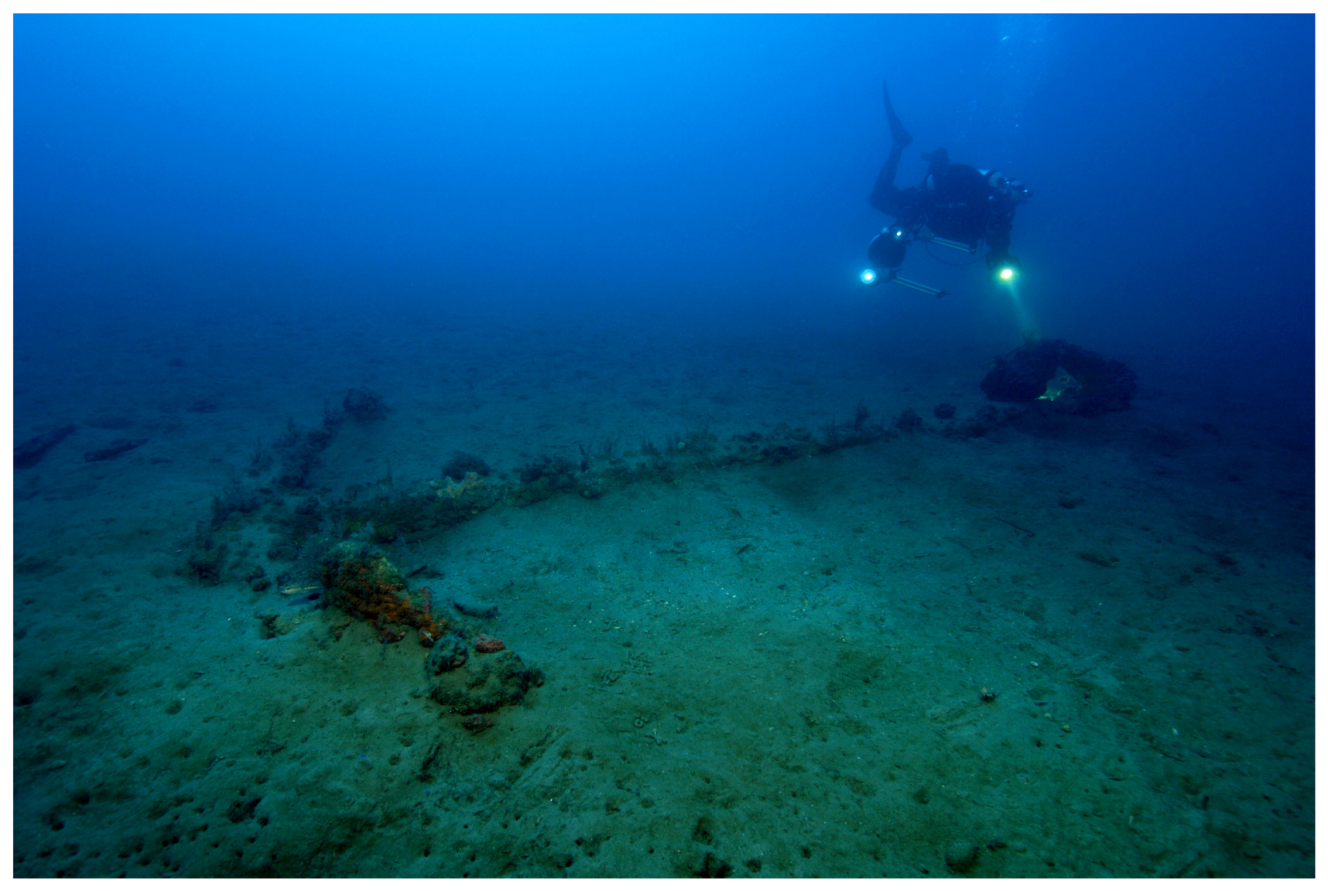

**Figure 32.**
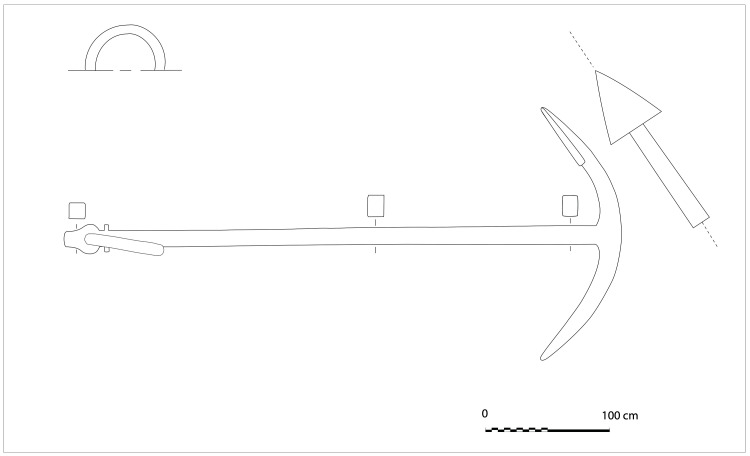
Anchor F-MIII-AW of the *Mortella III* wreck. Drawing Arnaud Cazenave de la Roche.

**Figure 33.** Anchor F-MIII-AW of the *Mortella III* wreck – © All rights reserved, Christoph Gerigk.

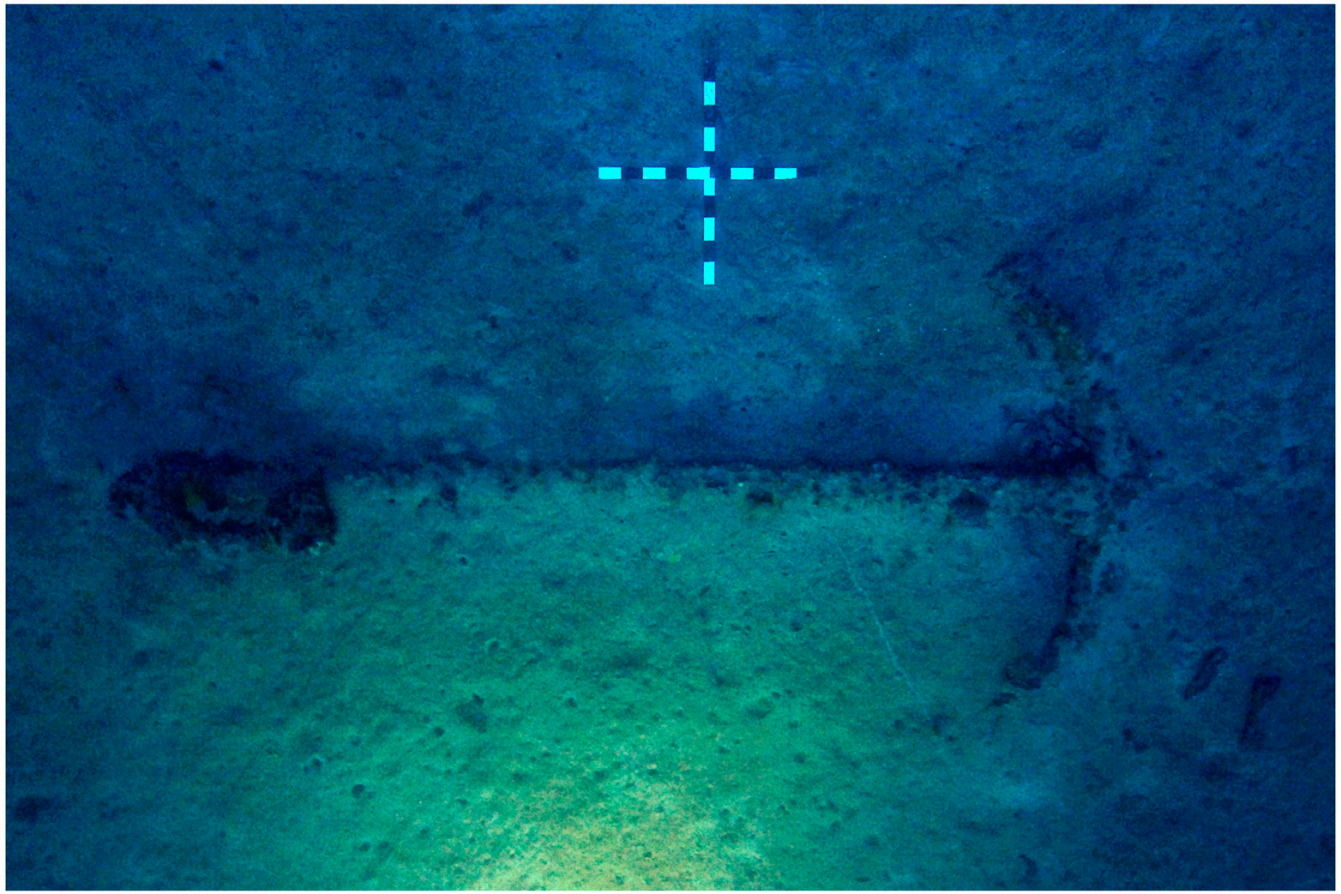

**Figure 34.**
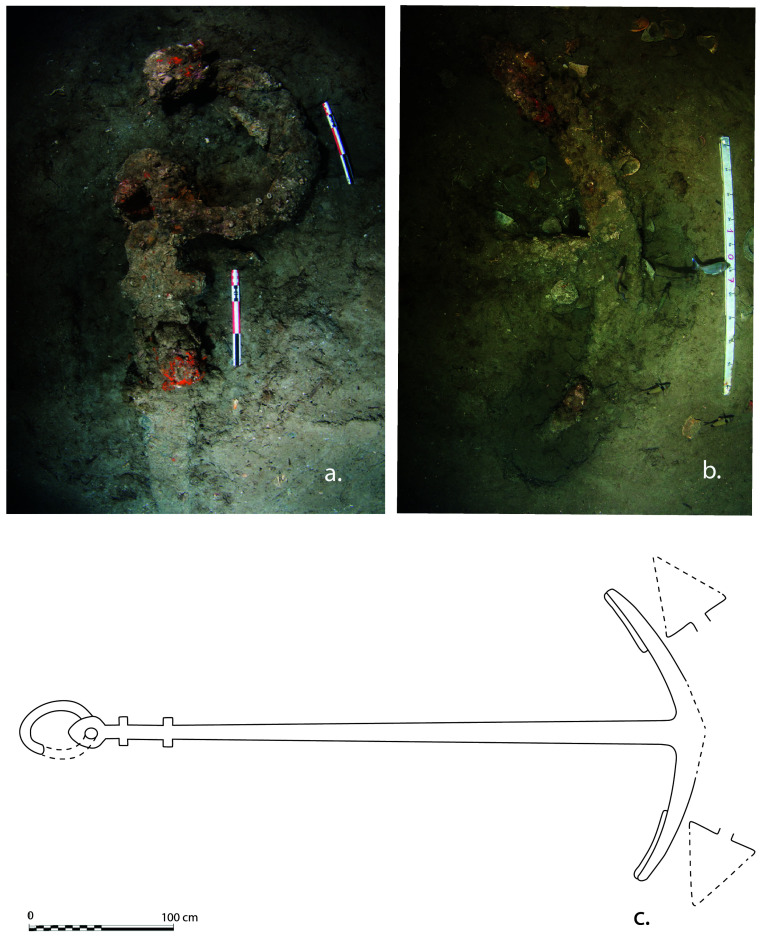
Anchor F-MIII-AS. The arms ( **a**.), the ring and the two pairs of nuts ( **b**.). Photos Stéphanne Jamme. Drawing Fabrizio Ciacchella.

Only two anchors have been found on the wreck, one 15 m west of the bow (F-MIII-AW, initially called ‘West anchor’), the other was 20 m south-east of it (F-MIII-AS, initially called ‘South Anchor’). F-MIII-AW has been recorded during the 2015 campaign ( Ciacchella, 2020, 211–213). F-MIII-AS has been excavated and recorded during the 2019 campaign. A third anchor (F-MIV-AI), ^19^ an isolated find with the arms buried in the muddy sediments of the sea bottom and the shank standing vertically, is about 800 m south of the *Mortella II* site and 120 m south of the *Mortella III*, and was formerly thought to have belonged to the latter ship. Research carried out during the 2021 campaign has shown that it has characteristics unknown in Renaissance anchors (like its straight head with no lateral expansions and its nuts perpendicular to the arms), that can only be found since the late 17 ^th^ century.

***5.2.1 Morphology*.** The two *Mortella III* anchors had a wooden stock that didn't survive, and were similar for many other characteristics: triangular flukes, curved arms following two different circumferences, nuts parallel to arms (i.e. stock keys on the same plane of the arms), head expanding laterally on both sides and ring. Shank and arms seem to have a squared cross-section, but it is difficult to state it with certainty over the concretions, and a thicker layer doesn't allow the presence of bills at the arms’ tip to be surely identified. The two anchors also have a few important morphological differences. The head of both the anchors are expanded laterally and oval-shaped, but the one of F-MIII-AW has a flat prolonged top. Over the concretions, its crown is rounded, while the one of F-MIII-AS seems slightly angled. The latter anchor has a very rare feature, two pairs of nuts instead of one. The morphological characteristics of the *Mortella III* anchors are resumed in Table 7. This type of anchor is often called 'admiralty'; though common, this is quite a confusing definition, because it could suggest some incorrect relation with the British Admiralty Longshank and British Admiralty Shortshank Anchors. Both more recent, their characteristics were very different from the so-called ‘admiralty’ anchors: the former designed in the late 17 ^th^ or the early 18 ^th^ century with straight arms, the latter in 1841 with curved arms on a single circumference, spade-shaped flukes, and round/oval cross-sections ( Curryer, 1999, 51, 73, 83).

***5.2.2 Measures, proportions and weight estimate*.** The measures of the *Mortella III* anchors, as well as their proportions and weight estimate can be found in Table 8. The principal measures of the F-MIII-AW are: length 452 cm, beam 205 cm, ring mean ^20^ diameter 56 cm, arms’ length 120 cm (measured from the crown), flukes’ length x breadth 58 x 49 cm. The angles between shank and arms, calculated by trigonometry at the crown, are 58°. The measures of the F-MIII-AS are: length 431 cm, beam 190 cm, ring mean diameter 48 cm, arm’s length from the crown (left-right) 110–117 cm, flukes length x breadth 54 x 54 cm. The angles between shank and arms are also different: the left 59°, the right 55°. Asymmetric angles are often found on old anchors due to frequent stress of traction during the use, but this doesn't affect arm’s length. The case of F-MIII-AS is different, and though deformation can't be excluded as a cause of angle asymmetry, the different length of the arms can only be the result of inaccurate forging.

Besides the morphological resemblance, the two anchors have some proportions in common:

- Beam is around 3/7 of shank length.
- Shank length is c. 3 ¾ times arm length, measured from tip to crown (considering the average of the asymmetric arms of F-MIII-AS).
- Flukes’ length is slightly less than half the arm length, measured from tip to crown.

However, they differ in many important other ways. Compared to F-MIII-AW, F-MIII-AS has:

- a proportionally smaller ring (mean diameter 1/9 of shank length, instead of 1/8),
- a more tapering shank (upper end width around 1/2 of lower end width, instead of 2/3),
- a less slender shank (lower end around 1/20 of shank length, instead of 1/24)
- triangular flukes that can be inscribed in a square (i.e. the height equal to the base, instead of longer).

Concretions thickness, estimated to 1.5 cm, has been subtracted from the anchors measurements, then the volumes of flukes, arms, shank and ring have been calculated, summed, and finally multiplied by the density of iron (7.87 kg/dm ^3^) to estimate the anchors weight. According to these calculations, the weights of F-MIII-AS and F-MIII-AW are respectively c. 800 kg and c. 840 kg. The difference between the two corresponds almost exactly to one Genoese *cantaro* (47.65 kg) and to one Spanish *quintal* (46 kg). Examples of anchor fittings with this difference can be found in a Spanish treatise written by Chaves c. 1537, and in the inventory of the Genoese *nave ‘Santa Maria della Carità’* of 1579 (see below in the text).

***5.2.3 Parallels*.** The *Mortella III* anchors are among the largest in the Mediterranean area, following three isolated anchors 5.20 m long – one recovered from Sa Mola, in the Spanish island of Formentera ( Hermann, 2013, 299–301), another from Agropoli, Italy, and a third one lying on the sea bottom near Camogli, Italy – and another one 5.00 m long in the *Piedra Que Revienta* wreck, 3 nm off Cádiz, Spain, ^21^ and finally anchor 1 of the Gnalic, wreck, (Croatia) 4.86 m long. From a morphological point of view, some characteristics are common to all 16 ^th^ century anchors: the head expanded on both sides, the nuts parallel to arms, and the triangular flukes. For its double pair of nuts, F-MIII-AS has two parallels ^22^: the above-mentioned anchor of Camogli, and anchor 1 (the smallest) of the *Sveti Pavao* wreck ( Beltrame *et al.*, 2014), Croatia, a Venetian ship sunk in the late 16 ^th^ century. The triangular flukes that can be inscribed in a square (i.e. with the base equal to the height), are similar to those of anchors 1 and 2 of the *Sveti Pavao* wreck, with anchor 1 of the *Gnalic* wreck (identified as the *Gagliana grossa*, a Venetian ship sunk in 1583), and with the anchor from Sa Mola, Formentera, Spain. The head of F-MIII-AW, expanded laterally and oval-shaped with a prolonged flat top, holds a close resemblance to anchor 1 of the Gnalic wreck and to the Sa Mola anchor, while its triangular flukes are more elongated, with a ‘base-to-height’ ratio similar to the one of *Sveti Pavao* anchor 3 ( Ciacchella, 2015, 57–65).

***5.2.4 Anchor fittings of early 16 ^th^ century Genoese ships according to historical sources*.** No Genoese source explains the anchor fittings of ships at the time of the *Mortella III* wreckage. To find some data, we have to get back to a maritime law of 1498, the *Nova forma pro navibus.* It concerned the *navi* over 10,000 *cantari* of net deadweight, and established their standard equipment: when sailing on Mediterranean routes or to Cádiz, they had to carry eight large anchors plus a small one for kedging. ^23^ Travelling farther, this amount had to be augmented at the discretion of the authority. No indication was given about the weight of anchors, probably because it was known from previous regulations. This fitting is not far from that of a four-masted *nave* of unspecified deadweight and tonnage, property of Angelo Lomellini, which in 1495 had seven anchors of 14 and 15 *cantari* (667–715 kg), plus a kedger ( D'Albertis, 1893, 229, 232–233).

The above-mentioned is the last Genoese law dealing with anchor specifications. The other available data come from inventories and contracts of ship construction or loan from the mid-16 ^th^ century. In 1557 the *Santa Maria*, a *nave* of 8000 *cantari* of net deadweight (381 t) and 2000 *salme* of net tonnage (533 m ^3^), also known as *Bertorota* after her owner, had four anchors of unspecified weight plus one kedger. Her keel and her hull were 19 m and 27.5 m long, much smaller than the *Mortella III* ship ( Borghesi & Calegari, 1970, 15–17, 93–116). In 1579 the *nave ‘Santa Maria della Carità’* of 10,400 *cantari* of net deadweight (496 t), had four anchors: three of 20–21 *cantari* (952–1000 kg), and one of 16 *cantari* (762 kg) ( Gatti, 1999, 327–337). The latter was too heavy to be the kedger of that ship, being 76% of the weight of the largest anchor on board, while in the only Genoese example known, listed in the statute of Gazaria of 1441, the kedger was 43% ( Pardessus, 1837, 465–489). In 1588, the *‘Santissima Trinità di Scala’*, a *nave* of 16,000–18,000 *cantari* of net deadweight (762–858 t), had six anchors of unspecified weight when she joined the *Gran Armada* ( Ridella, 2011: 50).

Some more elements of the number of anchors on board ships of the early 16 ^th^ century can be found in the Spanish treatise *Espejo de navegantes*, written by Alfonso Chaves, c. 1537. It gave the example of a ship of 200 *toneles* of net tonnage (304 m ^3^) with her five anchors: the heaviest (sheet anchor) weighed 11 *quintales* (506 kg), the others were 10 *q* (460 kg), 9 *q* (414 kg), and two of 7 *q* (322 kg). Kedgers were not mentioned, and no explanation was given about the way of determining the weight of the anchors ( Chaves, c. 1537, f° 60V-61R).

Lacking coeval Genoese sources, it is not possible to know whether the anchors’ fittings of the ships of the early 16 ^th^ century followed the rules of the late 15 ^th^ century or the habits of the mid- or late 16 ^th^ century. In the former case, the *Mortella III* ships would have had eight anchors, in the latter four to six and in both cases there would have been the addition of one kedger. On the wreck site only two anchors have been discovered; two to six large anchors (plus one kedger, if it was not on board the boat used by the crew to escape) are still missing. Because of the depth of the site (37 m), it seems unlikely that they had been salvaged by Genoese divers of the time, the famous *margoni*, even if their maximum operational depth is not exactly known. The reason for the difference between the expected number of anchors and those actually found, can be either that the ship was navigating with an incomplete set, or that many of them are still hidden under the sediments. Further instrumental magnetic research could reveal if others are present and allow a better understanding of the anchors’ fittings of Genoese ships of the early 16 ^th^ century.

**Table 7.** Morphological characteristics of the *Mortella III* anchors.

THE *MORTELLA III* ANCHORS	F-MIII-AS	F-MIII-AW
head form	expanded laterally, oval-shaped	expanded laterally, oval-shaped with prolonged flat top
nuts (stock keys)	2 pairs, parallel to arms	1 pair, parallel to arms
shank cross-section (inferiorly)	squared?	squared?
crown	slightly angled?	Rounded
arms	curved on 2 different circumferences	curved on 2 different circumferences
arms cross-section (internally)	squared?	squared?
flukes	triangle (base = height)	triangle (base < height)
bills	not identifiable	not identifiable

**Table 8.** Measures, proportions and weight estimate of the *Mortella III* anchors.

THE *MORTELLA III* ANCHORS	F-MIII-AS	F-MIII-AW
measures over concretions	(cm)	(cm)
shank length x beam	431 x 190	452 x 205
ring internal-external diameter (mean diameter)	41-55 (mean 48)	48-64 (mean 56)
ring thickness	7	8
shank width x thickness @ base of the head	12 x 10	13 x 13
@ small of the shank	13 x 12	13 x 13
@ large end of the shank	21 x 17	19 x 14
arm length (tip to crown) (Left | Right)	110 | 117	120 | 120
arm width x thickness @ big of the arm (Left = Right)	18 x 17	20 x 14
@ small of the arm (Left = Right)	16 x 14	13 x 13
@ arm tip (Left = Right)	6 x 6	6 x 6
fluke length x breadth (Left | Right)	52 x 54 | 54 x 54	58 x 49 | 58 x 49
angle shank-crown-arm tip (Left | Right), calculated by trigonometry	59° | 55°	58° | 58°
**proportions**	(ratios)	(ratios)
'mean diameter of ring to shank' ratio	0.112 (=1/9)	0.124 (=1/8)
shank 'taper' ('min width to max width' ratio)	< 0.57 (≈1/2)	< 0.68 (≈2/3)
shank 'slenderness' ('max width to length' ratio)	<< 0.049	<< 0.042
'beam to shank length' ratio	0.44 (≈3/7)	0.45 (≈3/7)
'shank to arm' (length @ crown) ratio (Left | Right)	3.92 | 3.68	3.77 | 3.77
'fluke to arm' (length @ crown) ratio (Left | Right)	0.47 | 0.46	0.48 | 0.48
fluke 'slenderness' ('base to height' ratio) (Left | Right)	1.04 | 1.00 triangle: base = height	0.84 | 0.84 triangle: base < height
**weight estimated after volume** (after subtracting estimated concretion thickness 1.5 cm)	c. 800 kg	c. 840 kg

### 5.3 Ceramic finds from the *Mortella III* and *Mortella II* wrecks ^24^

***5.3.1 Methodological aspects*.** The ceramic finds recovered during the excavation campaigns carried out on the *Mortella III* and II wrecks are certainly limited in terms of quantity, but still have interesting elements, primarily determined by the date of the sinking of the two ships in August 1527 ( Cazenave de la Roche, 2021, 204–207). This chronology appears absolutely compatible with the precise chronological elements deriving from the dendrochronology of *Mortella III*’s timbers.

The small number of ceramic fragments found during the excavation works and the fact that they belong to artefacts of different ‘ceramic classes’ ( Milanese, 2009) and shapes, represents a decisive element for interpreting the ceramic context as on-board tableware and not as pottery vessels on board for their marketing.

The analysis of the ceramic material presents elements of particular complexity, in addition to those due to the typical post-depositional factors caused by the chemical-physical erosion of the sea and by the invasive and aggressive biological action of marine organisms colonizing the ceramic surfaces.

**Figure 35.**
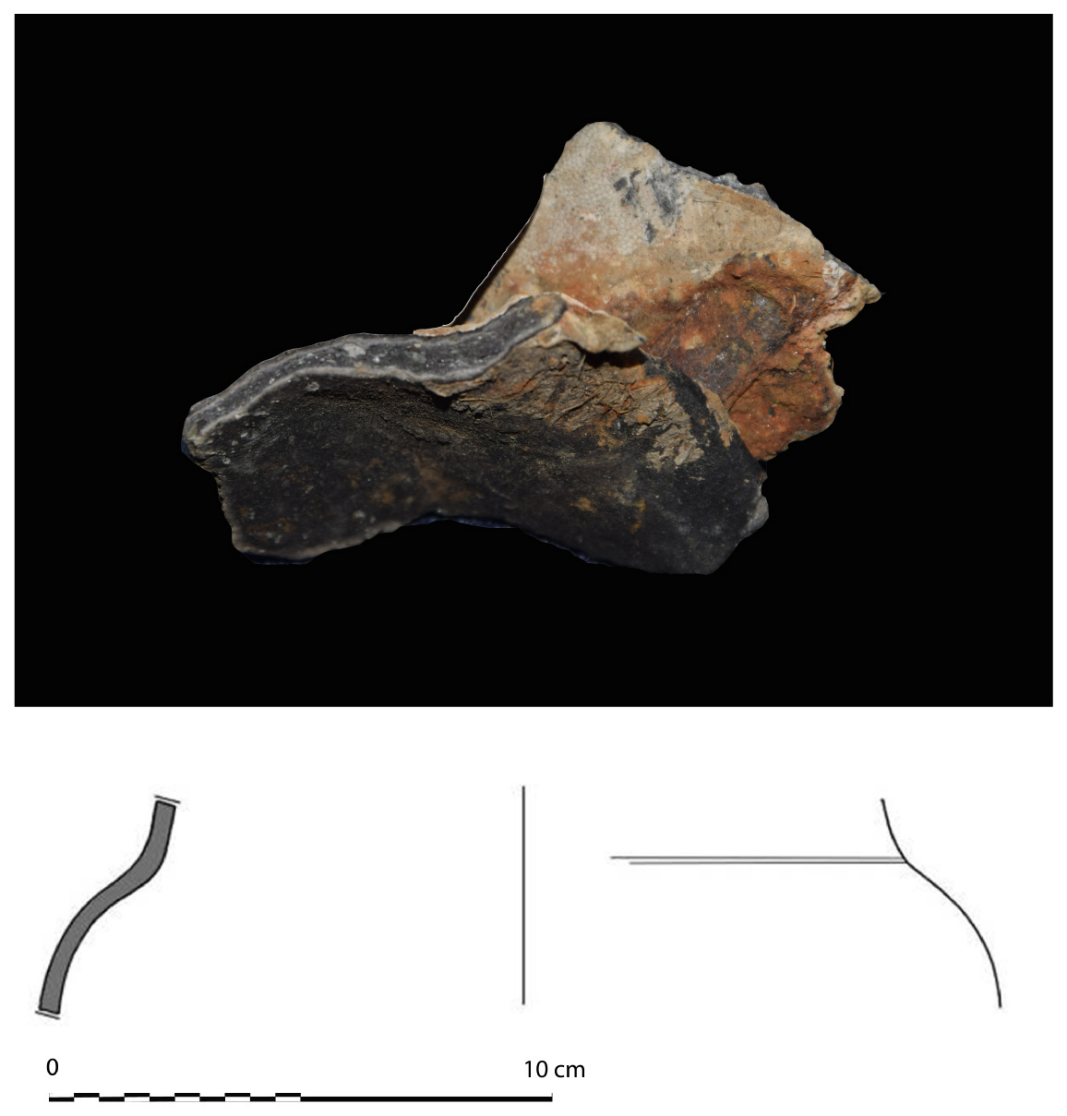
Sherd n° MIII-12-049. Photo Marco Milanese. Drawing Franck Allegrini.

These are unusual pre-depositional factors, determined by a violent fire perhaps intentionally set by the crew when abandoning the ships to prevent them from falling into enemy hands ( Cazenave de la Roche, 2021, 206). The very high temperatures reached by the fire of the ships determined a new partial firing of the ceramic artefacts on board, which are determinable and distinguishable in the very nature of the vitrified coatings (lead and stannifera), albeit with some difficulty and some uncertainties in identification ( Figure 35).

Coatings and ceramic bodies are in fact largely calcified, due to the temperature, with macroscopic characteristics completely different from the original ones. The study conducted on the materials therefore followed an indirect-deductive method, with the construction of a circumstantial framework, to allow the recognition of the ceramic classes to which the finds belong.

The morphology of the pottery and the traces of the decorations, despite the loss of the original characteristics of the coatings and ceramic bodies, nevertheless allowed us to build hypotheses about the identification of significant ceramic classes for the chronology of the archaeological context.

***5.3.2. Chronological markers*.** At least two ceramic classes are documented to date, which make it possible to compare in a very useful and interesting way with the chronology of the *Mortella III* wreck and the sinking of the ship (1527). The same preliminary considerations are then also extended to the few finds from the *Mortella II* wreck.

**Majolica of Montelupo Fiorentino**

Particularly important is a small fragment (MIII 2014-003) with a slightly everted rim, with a rounded margin and underlined by a groove, referable to a plate (dish). The fragment shows traces of a thin coating, in poor condition, on a clear ceramic body, still partially recognizable as such, despite a grey chromatic transformation, due to fire damage.

**Figure 36.**
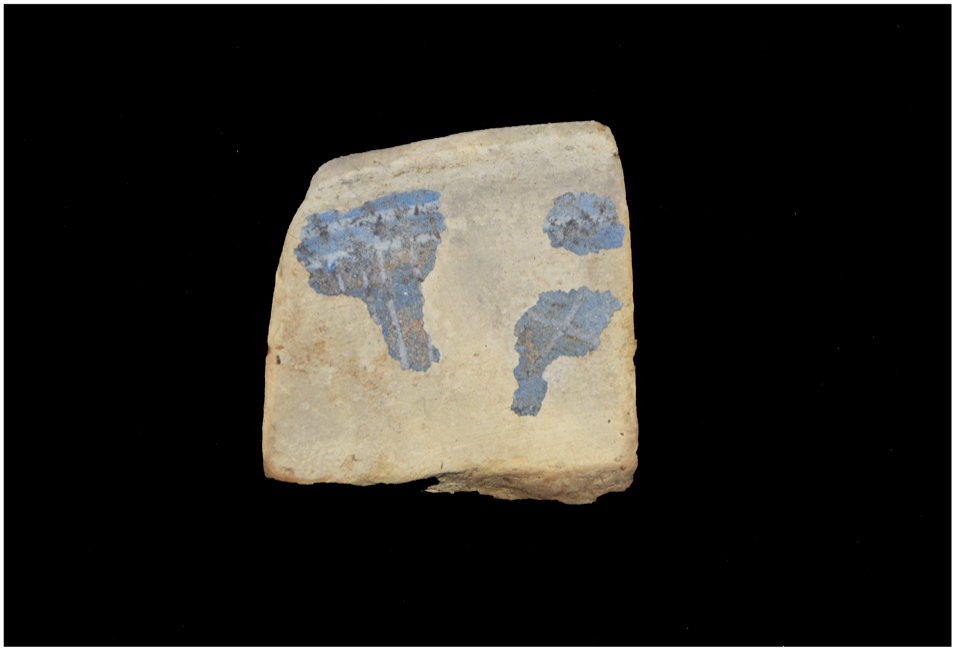
Sherd n° MIII-14-03. Photo Marco Milanese.

While on the outside the coating has no decoration, on the inside there are much damage traces showing a thin blue tin-glazed ( Figure 36): This is probably the only case in the *Mortella III* wreck, in which it is possible to recognize with certainty a fragment ascribable to a *majolica* of Montelupo Fiorentino. This recognition is made possible by the convergence of morphological characteristics, the presence of a tin-glaze which, although degraded, is recognizable as such and displays faint but clear residues of the decoration.

On the inside of the sherd, above a blue background, it is possible to recognize a decoration in white, produced with graffiti signs that have been engraved on the blue background: these characteristics link the sherd to the ‘Blue Graffito’ decoration and in particular to the ‘Blue Graffito Band’ of Montelupo Fiorentino, which is widespread both in its area of production ( Berti, 1998, 131) and in a vast Mediterranean and Atlantic area of the Mediterranean and Atlantic ( Fornaciari, 2016, 33 ff.). In fact, the decoration below the rim has a ‘blue graffito band’ (‘a quartieri’) and fits well with the classification provided by Berti from the excavation of the *Pozzo dei Lavatoi* of Montelupo (Group 34.2) ( Berti, 1998, 133 and 122), with a proposed chronology of 1510–1520.

The small sherd has a simple rim without a brim and with a weak groove near the margin of the everted rim: it finds an interesting comparison with the shape (dish) Aa 2.3.2. l of the 2016 Fornaciari classification ( Fornaciari, 2016, 100), datable to the first decades of the 16 ^th^ century. But it is above all from the blue graffito band decoration that we can deduce important chronological references. This decoration is in fact fully compatible with the chronology of the wreck (1527): it is possible to recall the discovery in Montelupo, during the excavation of the *Pozzo dei Lavatoi*, of a tin-glazed tableware pottery plate with a blue *sgraffito* band bearing the date painted when produced ‘1514’ ( Berti, 1998, Group 34.2, fig. 120 and p. 137), also from the same excavation context, of a plate with a blue *sgraffito* band and papal insignia of Leo X (Giovanni di Lorenzo de Medici) (1513–1521), referable at the triumphal entry of Leo X into Florence and therefore can be dated to 1516 ( Fornaciari, 2016, 100).

The identification of a Montelupo plate of the "blue graffito band" type in the *Mortella II* wreck, whose shipwreck occurred in 1527, also represents an important element for the study of the chronology of this decorative genre, which is instead absent among the pottery from the wreck of the Genoese ship of the *Lomellina*, which was wrecked in 1516 ( Guérout *et al.*, 1989).

**Imitations of Montelupo Fiorentino *majolica***

**Figure 37.**
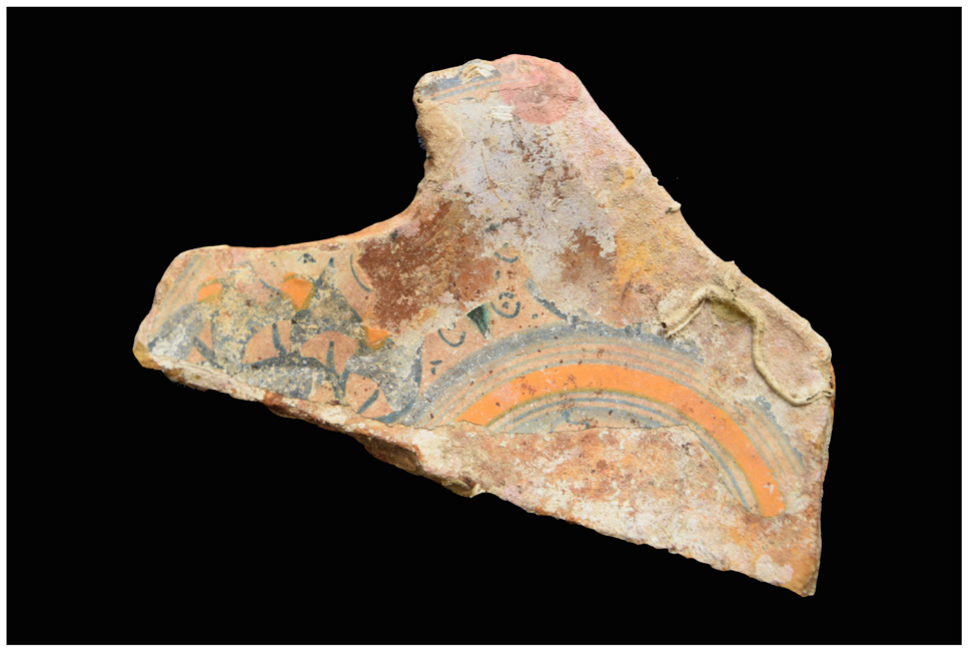
Sherd n° MII-01-02. Photo Marco Milanese.

**Figure 38.**
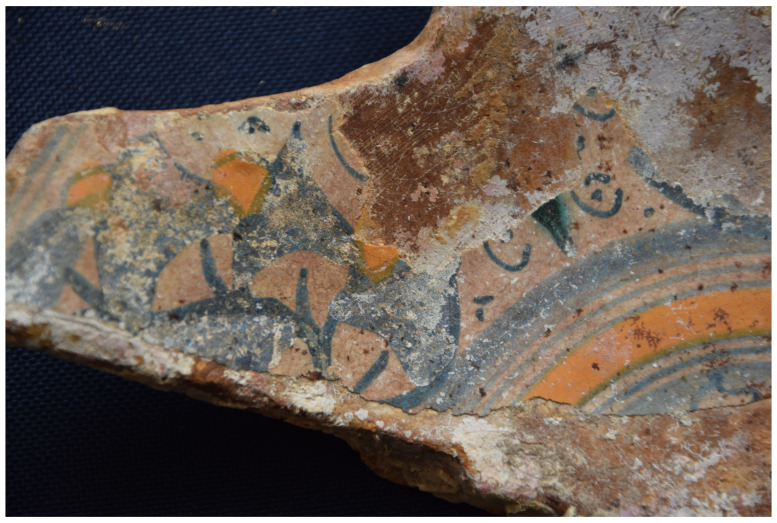
Sherd n° MII-01-02. Detail. Photo Marco Milanese.

**Figure 39.**
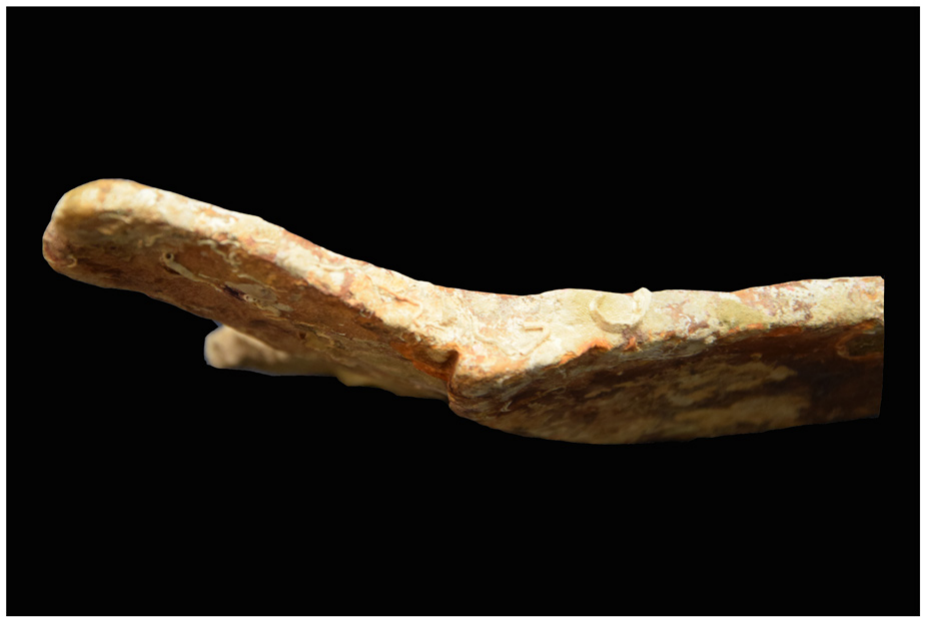
Sherd n° MII-01-02. Edge profile. Photo Marco Milanese.

It is also necessary to mention a large fragment of plate ( Figure 37), coming from the *Mortella II* wreck, considered a ‘twin’ of the *Mortella III* wreck and therefore shipwrecked at the same time as this one here in August 1527. Despite large gaps in the very thin tin glaze film and a notable deterioration due to marine organisms and almost five centuries of underwater lying, it is possible to recognize a large horizontal band, between the bottom of the plate and the rim, with a decoration belonging to the repertoire known in the production of Montelupo, as ‘Persian *palmette*’ ( Figure 38) ( Berti, 1998, Genre 21). This can be chronologically placed between 1480 and 1520 ( Fornaciari, 2016, 55). The profile has an indistinct edge, only slightly splayed, and a bottom without a distinct foot ( Figure 39).

However,this sherd has some characteristics that make it recognisable as an imitation of Montelupo *majolica*, to be attributed to a different production centre: a very intense blue, typical of the ‘Persian *palmette*’ of Montelupo, in the dish from the *Mortella II* wreck is obtained instead with a very diluted light blue, which is not comparable even in the much more recent (1560–1620) motifs of the ‘Genre 53 - Extenuation of Renaissance motifs’ ( Berti, 1998; Fornaciari, 2016, 55).

There are still two factors to mention: the decoration, with a painting of lesser quality, which confirms the difference of this sherd from the models of Montelupo and the same ceramic body of pink colour, which seems not to have anything in common with those of the XVI century production of Montelupo. The *Mortella II* sherd is particularly interesting because it seems to refer to a centre that already in the third decade of the 16 ^th^ century imitated the *majolicas* of Montelupo, and therefore allows us to suggest an early chronology of the more general phenomenon, still largely to be studied, in which it is possible to join the sherd himself.

The enormous quantity of tin-glazed tableware pottery produced in Montelupo, especially between the 15 ^th^ and 17 ^th^ centuries, the great commercial diffusion of these products firstly through the ports of Pisa and then Livorno from the 16 ^th^ century, and above all the traditional mobility of the ceramic workers, these are three of the main factors that have favoured imitation productions of Montelupo *majolica* in many Tuscan production centres and also outside Tuscany.

Only some of these imitation production centres are recognized today (thanks to archaeological finds or written documents), but this phenomenon was certainly wider. To date we have documentation for Rome (summary in Fornaciari, 2016, 31, notes 61 and 62), Arezzo ( Milanese, 1994, 93), Montepulciano ( Milanese, 1994, 100), Alto Lazio, Livorno, Siena and Northern Europe.

***Sgraffito* pottery**

Some sherds that show traces of a slip layer and a sgraffito decoration obtained with a pointed tool require specific discussion, because despite their extremely damaged storage conditions and largely lost legibility, they enable a precise characterisation. The fragments are referred to as the shape of a basin that has a thin engobe layer inside the artefact, with occasional dripping on its outside. The decoration is executed using the sgraffito technique, the lines of which are obtained by an extremely brief and rapid movement, only on the inside of the vase ( *Mortella III*-12 0035 F; 0037). The technological characteristics of the lead-glazed layer and those of the ceramic body are destroyed by the violent action of fire, which in some cases has produced a partial fusion and new firing of the ceramic bodies, also with a distortion of the shape with respect to the original profiles of the objects (eg. *Mortella III* – 12 0037).

**Figure 40.**
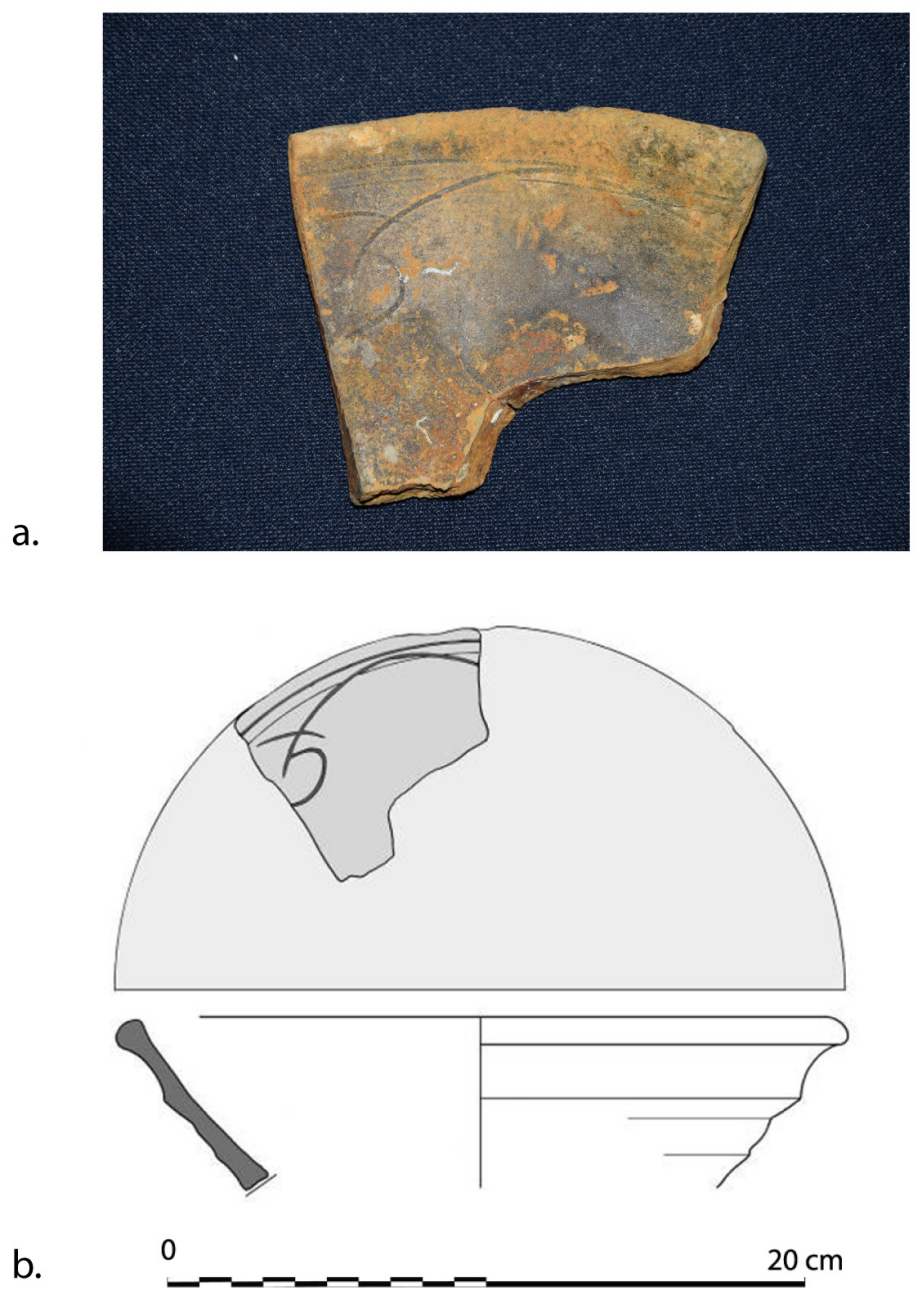
Rim of *graffito* basin (MIII-12-0037). **a**. Photo Marco Milanese. **b**. Drawing Franck Allegrini-Simonetti.

The recognizable shape of the basin has a wide inclined wall, the profile of which is interrupted, in the upper part, by a weak hull, not very pronounced, while the rim shows an enlarged margin ( Figure 40).

This basin shape has a wide sloping wall, the profile of which is interrupted in the upper part by a weak hull, not very pronounced, while the rim shows an enlarged margin ( Figure 6).

The decoration is obtained with a pointed tool and is organized with an upper band defined by sgraffito lines, inside which there are some extremely schematized decorative motifs, such arches arranged horizontally and stylized leaves, in an almost abstract way. The original characteristics of the ceramic bodies are no longer legible, but the particle size is still observed and it is generally very fine and compact.

For these characters just mentioned (shape of the basin with a thickened edge and weak hull, engobe and lead-glaze layers absent on the outside, completely cursive and synthetic decorative syntax, fine-grained ceramic body) it is believed that it is possible to attribute these findings to truncated conical shape basins with subtle *sgraffito* decorations, produced in Pisa and framed between 1500 and 1530, with continued circulation in the following decades ( Alberti & Giorgio, 2013, 94–96 and 99–100).

***5.3.3 Discussion*.** The analysis carried out on the ceramic sherds leads us to believe that these are attributable to the vessels on board the ship's General Staff and therefore used by the Captain, the Purser, the Officers, the Doctor or on-board managers. These were *majolica* vessels from Montelupo: a plate from *Mortella III* and another one (an imitation of Montelupo) from *Mortella II*, in addition to one or more truncated conical *sgraffito* basins of Pisan production and a few other fragments whose study is made difficult by the degradation caused by the fire that consumed the ships.

However, these finds are precisely placed in the first decades of the 16 ^th^ century, as discussed in detail and are therefore well compatible with the dating of the sinking of the ships in 1527. The ceramic indicators also agree with the dates provided by the dendrochronology of the timber of the *Mortella III* wreck.

It is also the small quantity of the ceramic fragments that suggests the interpretation that the pottery was in use by a few people, the Commander and the crew members of higher rank and responsibility, who could take advantage of a more varied food supply, reserved for them, with better quality foods that justified the use of *majolica* plates of a certain value. On the other hand, it is possible to believe that the sailors had mainly wooden dishes, including bowls and spoons for a diet based on simple soups with savoury biscuits, widely used in long voyages, with small portions of meat or fish, salted or dried. A fragment of only one lead-glazed pot poses the issue of the kitchen and its location in the ship (forward or at midship). The ceramic context also suggests that a merchant ship of this size should also have large copper pots on board, for the preparation of liquid or semi-liquid food for a certainly large crew.

## Conclusion

The *Mortella* wrecks are precious witnesses of a Mediterranean shipbuilding tradition at the dawn of the modern period. At that time, the ship was an essential tool both as a vector of trade and as a war machine, but also as an instrument of discovery and conquest of the world. Today, our vision of European shipbuilding lacks the Mediterranean technical culture which is the origin of many innovations and technological transfers to the Atlantic. In this sense, the *Mortella* wrecks contribute to generating knowledge that is indispensable for our understanding of the European naval organisation and its role in the development and expansion of European nations.

In view of the above, the excavation and study of the *Mortella III* wreck usefully completes the meagre *corpus* we have so far and, together with the Villefranche-sur-Mer and Calvi I wrecks, provides us with documentation likely to lay the foundations of its knowledge. This trio of wrecks opens more specifically a window on the architecture of a typical Genoese merchant ship in the 16 ^th^ century, the *nave*, a type of ship of great importance in the maritime trade of the time. Other wrecks of Mediterranean tradition, such as the *Santiago de Galicia*, currently being excavated in Spain, together with others, especially in Croatia, open up the prospect of completing this *corpus.*

From the point of view of construction processes, the *Mortella III* wreck has highlighted particular characteristics common to a Mediterranean technical culture, especially in terms of the methods of assembling and fastening pieces, the morphology of the keel, the mast-step or the pump system ( Cazenave de la Roche, 2020c, 154–155). However the definition of the ‘Mediterranean profile’ of the construction cannot be addressed without taking into consideration the architecture of the *nave* in terms of shapes and proportions. From this point of view, the three wrecks have a similar transverse shape, characterised by a circular design of the main section, at least at the level of the lower works, which contrasts with the fusiform design of the Venetian *navi* as described by Venetian authors between the 15 ^th^ and 16 ^th^ centuries. This aspect of the construction is essential, as it is likely to highlight regional differences, in this case between Venice and Genoa, which would not be apparent from an analysis of carpentry techniques.

The same applies to the proportions given by the builder to his ship. In the case of the *Mortella III* wreck, the height of the remains of the hull, interrupted at 2.10 m above the keel, makes it impossible to determine with certainty the shape of this profile, more or less above the lower half of the depth of hold. As a result, two hypotheses have been formulated: according to these, the maximum breadth would be established at 11.60 m in the first and 10.40 m in the second. In the first case (H1) this alternative results in a ratio 1 : 2.24 : 3.17, close to the ‘ *As-Dos-Tres’* rule, similar to the Calvi I wreck (1 : 2.18 : 3.19). In the second case (H2), the ratio is 1 : 2.50 : 3.54, almost identical to that of the Villefranche-sur-Mer wreck (1 : 2.56 : 3.52).

The same applies to the height of decks, for which the earliest hypothesis formulated in the monograph on the wreck could be revised. The *nave* appears to be a ship currently provided with three decks: the Venetian and Ragusan texts mention two *coverta* and one *tolda*. The second *coverta* was close to the maximum breadth where the waterline was also located at maximum load. In the case of the *Mortella III*, this leads us to conclude that the height of second deck was probably situated between 5.20 m (14 *goa*) and 5.80 m (15 3/5 *goa*), depending on hypothesis H1 or H2 (4.27 m in our initial estimate). It also led us to a revision the tonnage estimates. For the larger ships, the presence of an orlop was structurally necessary. We concluded that the *Mortella III* ship did not have an orlop, unlike the Villefranche-sur-Mer ship, where the remains of the beams located 2.50 m above the keel are clearly the remains of an orlop. 

In view of the above, the characteristics of the geometry of the *Mortella III* ship are currently the subject of a new study which includes the comparative analysis of texts, in particular various construction contracts for Genoese *navi* of the 16 ^th^ century from the Archives of the State of Genoa (ASG), the aim of which is to approximate the rules of architectural proportion which govern the construction of this type of ship. This evolution of our vision of the *nave* shows, if need be, that we are dealing with a highly dynamic field of research. As time goes by, thanks to the research effort and the new information that archaeology and literature can provide, corrections will be made, some hypotheses will be confirmed, others will be invalidated, and our knowledge of the *nave* and Mediterranean shipbuilding will be refined.

Finally, the richness of naval architecture study of the *Mortella III* wreck must not minimize the importance of the contribution of its artefacts for which the study opens an interesting insight into the material culture of the period. It is worth adding that it also helps us to gain a better understanding of the ship architecture.

The artillery and the anchors are the main metallic artefacts observed and studied on the wreck. As far as the artillery is concerned, only wrought iron pieces and stone shot have been found. According to the Genoese maritime law *Nova forma pro navibus* of 1498 ( D'Albertis, 1893, 232), a couple of bronzes cannon should have been on board ships larger than 10,000 *cantari* (477 t of net deadweight). Their absence from the site can be due either to the fact that they would have been recovered at the time or later, or that the ship was navigating with less armament than stated by law. On another hand, the anchors of the *Mortella* wrecks give a rare opportunity to study the fittings of Genoese ships of the early 16 ^th^ century. Historical sources inform us about the amount and the weight of anchors throughout the whole 15 ^th^ century and how they changed in the second half of the 16 ^th^ century, but actually there are none from the time of the sinking of the *Mortella* ships. Only two anchors have been found up to date on the *Mortella III* wreck, but others can still be buried in the muddy sediments of the sea bottom. Further instrumental research could reveal them and show us whether the anchor fittings of this Genoese ship followed the maritime laws of the late 15 ^th^ century, or the habits of the second half of the 16 ^th^ century, or a transition between the two, even if she was navigating with an incomplete set. From a morphological point of view, the *Mortella III* anchors are even more important, as they are the first specimens studied on a Genoese wreck of the Renaissance, and no nautical treatise is known to illustrate how Genoese anchors were at the time. The study of those of the *Mortella III* wreck show some parallels with many others on the 16 ^th^ century from the Adriatic Sea, as if the Italian production of the time had some common traits, but we need more findings to confirm this and to understand if some regional differences existed.

Finally, the importance of the ceramic material found on the *Mortella II* and *III* wrecks is mainly due to the connection between the underwater archaeological context and the archival documentation, which allowed the sinking of the two ships to be precisely dated to 1527.

This enables the few ceramic finds to be considered as highly informative thanks to their nature and quantity, relating them to the General Staff's crockery in use by the Commander, the Commissioner, the Officers, the Doctor or those in charge on board, rather than a commercial cargo. A Montelupo *majolica* plate, embellished by a blue graffito, fits well the known timeline of this ceramic class, as do the graffito ceramics of Pisan make. It is relevant to note the absence of Ligurian blue-glazed *majolica* ( *berettino*), which at the time (1527) had likely not yet reached a Mediterranean spread as consistent as the one testified by the Corsican Brocciu wreck which is more recent (1555). A finding of a decoration traceable to Montelupo is to be considered an imitation made in a different manufacturing centre, representing extremely early evidence of the phenomenon of imitation, already documented in multiple locations throughout Italy and Europe, specific to such produce. The underwater context of the *Mortell*a wrecks is a true reference point for the study of the timeline of ceramic circulation in the early 16 ^th^ century Western Mediterranean.

## Data availability

### Underlying data

DIGITAL.CSIC: Mortella III wreck (1527, Corsica, France). Excavation report of the year 2019. 6th field campaign (22sd September to 22sd October 2019), Arrêté n°2019-308, OA3873 http://hdl.handle.net/10261/245875 ( Cazenave de la Roche *et al.,* 2020d)

This project contains the following underlying data:

•Technical Excavation Report on the Mortella III wreck (OA3873) 1527, Saint-Florent (Haute-Corse) Data of the year 2019 (Field Campaign from 22 sept. to 22 oct. 2019) Arrêté of French Government 13/09/2019 n°2019-308) (in French)

Figure 3, 4, 13, 14, 26, 29, 30, 31, 33 : © All rights reserved Photos ©Christoph Gerigk.

### Extended data

DIGITAL.CSIC: Mortella III wreck: Data and technical information 2010 – 2020 http://hdl.handle.net/10261/247291 ( Cazenave de la Roche *et al.,* 2020)

This project contains the following extended data:

• Mortella3_wreck_CONSTRUCTION-DATA

 DIGITAL.CSIC: Artifacts inventory of the Mortella III wreck: General inventory of the artifacts from the excavations of the Mortella III wreck between 2010 and 2020. http://hdl.handle.net/10261/249259 ( Cazenave de la Roche *et al.,* 2020)

Data are available under the terms of the Creative Commons Universal Public Domain Dedication (CC0 1.0).

Access to this dataset requires registration with a free IEEE account.

## Footnotes

^1^ The national archaeological map program is managed by the French Ministry of Culture. It lists all the archaeological heritage by means of a computer tool called ‘Patriarche’ which combines a database (DBMS) with a Geographic Information System (GIS).

^2^ The name of these sites refers to the Genoese tower of Mortella (16th century) which closes the bay of Saint-Florent on its western side and is the closest point of coast to the wrecks. Mortella I is an archaeological site of a traditional mooring place at the foot of this same tower.

^3^ The *nave* (plur. *navi*) belongs to a specific type of seagoing merchant ships of Genoese origin widely used in the Italian-influenced trade in the late Middle Ages and early modern period ( Figure 2).

^4^ The *ForSEAdiscovery* consortium is formed by an international group of institutions and researchers aiming to combine historical research, underwater archaeology, GIS and wood provenance methods.

^5^ The expression ‘fingerprint’ is borrowed from O. Crumlin-Pedersen ( Crumlin-Pedersen, 1991).

^6^ Among them is the *Santiago de Galicia* (1597, Spain) currently being excavated among other shipwrecks, some of them in Croatia.

^7^ The additional metre we gave is an estimate of the total length of the keel ‘bearing on land’ considering the flat part of the stem-post to its point of tangency.

^8^ *‘Contre-quille’* in the text.

^9^ It was primarily a study of the wood cells by F. Langenegger (OPAN, Neuchâtel, Switzerland) that showed that the frames had not been deformed. Secondly, the reassembly of the pieces, respecting the coincidence of the nail holes and the marks on the wood, made it possible to find their original position.

^10^ The main maritime units of measurement in Genoa were the *goa* (0.7432 m) and the *palmo* (0.2474 m). Italian maritime units of measurements, weights and volumes currently used in the 16 ^th^ century are presented. in Table 2.

^11^ ‘Misure di navilii’, 1567, Anonym, Biblioteca Nazionale Marciana di Venezia. B.N.M., mss. it. cl. VII cod. CXXV (7460). Italy. Transcription published by M. Nicolardi ( Nicolardi, 2014).

^12^ It should be noted that the Genoese and Ragusan notarial contracts ( Gatti, 1975) take the value of the depth of hold to the ‘ *prima coverta*’, while authors like Nicolò Sagri define its height up to the ‘ *seconda coverta*’. We have chosen to keep the latter definition, as it allows a better assessment of the ship capacity.

^13^ *‘As-Dos-Tres*’ is a rule originating from the Mediterranean Middle Ages. Widely used in the shipbuilding of the 16th century, it establishes a principle of proportionality between the maximum breadth ( *as*), the length of the keel ( *dos*) and the total length of the ship ( *tres*) in a ratio equivalent to 1 : 2 : 3.

^14^ In order to characterise the port-pieces in a homogeneous way, only the rings other than the muzzles reinforcements were taken into account.

^15^ ‘Inventaire de la *Louyse’*, Archives Municipales du Havre EE 79, Nov. 1516, f° 3v.

^16^ These shot are to be compared with the A 161 shot found on the wreck of the *Lomellina*, which has a diameter of 221 mm and a weight of 16.5 kg, giving a density of 2.96.

^17^ Crouy Chanel stated ‘The 28 port-pieces of the ‘great *nave*’ armed in 1489 at Villefranche are called ‘ *grosse*’ (i.e. big) (Crousy Chanel, 2020, 434).

^18^ An oral tradition of local divers reports that two bronze cannons were looted on the *Mortella III* site in the eighties.

^19^ F-MIII-AS and F-MIV-AI has been the object of two different reports for the French *Département des Recherches Archéologiques Subaquatiques et Sous-Marines* (DRASSM).

^20^ To minimize the effects of the concretions on the measurements of the ring, the outer and the inner diameter have been added together, then divided by two. The outer dimension has been chosen as the smallest external diameter, and the inner as the largest internal diameter, both of them taken over the concretions.

^21^ The author had the opportunity to study the anchor recovered from Agropoli and the ones in the sea near Camogli and in the *Piedra Que Revienta* wreck, still unpublished.

^22^ Another parallel can be found on the Internet, a drawing (not an archaeological one) of an anchor found in the gulf of Trieste in the 20 ^th^ century, made by Aldo Cherini, an amateur researching on maritime history and naval ethnography. It has two pairs of nuts, an oval head with a prolonged flat top, and was 4.47 m long and 2.95 m wide ( https://www.cherini.eu/etnografia/ancore/slides/an_0190.html visited on June 21 ^st^, 2021). Although the nuts are similar to those of F-MIII-AS and the head similar to the one of F-MIII-AW, its ‘beam-to-shank-length’ ratio is 0.66, very different from those of the *Mortella III* anchors, 0.42 ( Table 8).

^23^ The kedger was a small anchor used for kedging ( *tonezar* in Renaissance Italian), i. e. the action of moving a sailing vessel within a bay or upstream from the mouth of a river. When needed, the kedger was loaded on the ship’s oared boat, carried to the desired place, and then cast. A cable connected it to the ship that was moved toward it using the capstan.

^24^ This study has been conducted as a continuation of a first typological approach carried out in 2012 by Franck Allegrini Simonetti entitled ‘Première approche du mobilier céramique de l’épave de la Mortella III’ found in the unpublished report of the same year (Cazenave de la Roche *et al*., ‘Rapport de fouille programmée pluriannuelle du site de la Mortella III (St-Florent, Haute-Corse). Arrêté du 04/09/2012 n°2012-69’, p. 49-57.)
