# Supplementary material for: Review of the research programme on the Mortella III wreck (2010-2020, Corsica, France): A contribution to the knowledge of the Mediterranean naval architecture and material culture of the Renaissance
Source: Open Res Eur. 2022 May 18;2:6. Originally published 2022 Jan 11. [Version 2] doi: 10.12688/openreseurope.13942.2 (PMC10446101; doi:10.12688/openreseurope.13942.2)
Supplement: Peer review history

# Peer review: Reviewer response for version 2

**DOI:** 10.21956/openreseurope.15825.r29308

**Published:** 2022-06-07

**Authors:** Marcel Pujol i Hamelink

**Affiliations:** 1 Higher School of Conservation and Restoration of Bens Culturals de Catalunya, Barcelona, Spain

Thank you for having taken into consideration all the notes I made in the revision. The authors of this text have been working on it thoroughly for quite a long time and with great care.

If applicable, is the statistical analysis and its interpretation appropriate?

Yes

Is the study design appropriate and is the work technically sound?

Yes

Is the work clearly and accurately presented and does it engage with the current literature?

Yes

Are the conclusions drawn adequately supported by the results?

Yes

Are sufficient details of methods and analysis provided to allow replication by others?

Partly

Are all the source data and materials underlying the results available?

Yes

Reviewer Expertise:

NA

I confirm that I have read this submission and believe that I have an appropriate level of expertise to confirm that it is of an acceptable scientific standard.

# Peer review: Reviewer response for version 1

**DOI:** 10.21956/openreseurope.15026.r28626

**Published:** 2022-03-18

**Authors:** Niklas Eriksson

**Affiliations:** 1 Stockholm University, Stockholm, Sweden

I have read the ’Review of the research programme on the *Mortella III *wreck (2010-2020, Corsica; France): A contribution to the knowledge of the Mediterranean naval architecture and material culture of the Renaissance’ with great interest.

 The paper summarizes a large and ambitious maritime archaeological project conducted over a period of fifteen years on the Mortella III wreck. Substantial parts of the wreck have been excavated and recorded.

 The study is described to have been conducted over three lines of research: naval architecture, material culture aboard and literary research. However, these ‘three lines’ could be regarded as to a large extent being intertwined or entangled, which is something I find very positive. The paper is thus a good example of historical maritime archaeology that combines and make use of both written sources, images and material remains, not only using archaeology to illustrate what the historians already knew.

 The combination of the source materials is used mainly in two different ways. To begin with there is a discussion on the identification of the wreck(s). This effort provides the archaeological material with a very specific societal context – the Genoese trade of the 16 ^th^ century – but also with some very specific events – when the ships were set on fire, due to the risk of being captured by French galleys. As the wreck remains has been identified as *navi*, they may also serve as representative and diagnostic for vessels of this type in a more general sense, something that is crucial also in relation to the ambition to identify what is referred to as ‘technical fingerprints’ (for instance p. 3, footnote 5).

 Moreover, the study makes use of a multiple of sources during the reconstruction of the vessel. For instance the main dimensions and the cross section is reconstructed from the wreck and with information from various Shipbuilding manuscripts are very interesting. The ships architecture is further described both by an iconographical source (Figure 2.) but also the location of the armament that reveal an impression of the extent of fore- and after-castles.

 Altogether the paper is well-researched and well-illustrated. It is easy to understand and make use of the content. Something which is common in manuscripts with several authors is that the disposition and style are not really entirely coherent. There is some repetition in the different parts of the manuscript. For instance section 2.2 is to an extent repeating what is said in the introduction (p. 3 in my copy). Perhaps section 2.2 could be moved to the introduction?

 Some other notes are that on page 8 it says that ‘The framing at midhip: 19 […] was identified as master-frame’, which is very important information, not least when comparing the wreck to shipbuilding manuscripts. It would thus be relevant to declare how this master frame was identified.

 Another thing that might require some consideration is on page 10. Here the butt-scarf of the keel is discussed and it is said that ‘As the keel is in contact with the water, it is understandable that a butt-scarf would generate the same principle of sealing through the swelling of the wood as it is used for the planking in the carvel technique’. However, this is only partly true as wood swells a whole lot more across the fibres than along the fibres.

If applicable, is the statistical analysis and its interpretation appropriate?

Yes

Is the study design appropriate and is the work technically sound?

Yes

Is the work clearly and accurately presented and does it engage with the current literature?

Yes

Are the conclusions drawn adequately supported by the results?

Yes

Are sufficient details of methods and analysis provided to allow replication by others?

Not applicable

Are all the source data and materials underlying the results available?

Yes

Reviewer Expertise:

Maritime archaeology, nautical archaeology, historical archaeology

I confirm that I have read this submission and believe that I have an appropriate level of expertise to confirm that it is of an acceptable scientific standard, however I have significant reservations, as outlined above.

# Response

**Published:** 2022-03-20

**Authors:** Arnaud Cazenave de la Roche

**Affiliations:** Spanish National Research Council, Spain

In this review, three main points appear to need clarification:

1. *Reviewer: ‘It would thus be relevant to declare how this master frame was identified.’ *
**Answer:** We suggest to specify this point: ‘The master-frame was identified as being provided by two first-futtocks attached on either side of the end of the same floor-timber, and as the reversal point of the sequence of attachment of the first-futtocks to the floor-timbers: aft toward the stern and fore toward the bow.’ However, the following points should be stressed: It is true that the identification and study of the shape of this master-frame of the wreck is crucial for the understanding of the hull design. However, as stated in the introduction, our paper attempts to synthesise the main features of the ship's architecture without repeating what has already been published. Consequently, as the manner in which this master-frame was identified, the manner in which it was brought ashore for study and the manner in which it was reconstructed is set out in the Mortella III monograph, it is not detailed in the text (Cazenave de la Roche, 2020: p.57-67 and p.117-131). Instead, as the introduction also states, the article intends to focus in detail on the new information from the latest excavation in 2019. *Cazenave de la Roche, A., 2020, The Mortella III wreck: a spotlight on Mediterranean Shipbuilding of the 16th century*. British Archaeology Report, BAR International Series 2976, BAR Publishing.
2. *Reviewer: ‘… section 2.2 is to an extent repeating what is said in the introduction (p. 3 in my copy). Perhaps section 2.2 could be moved to the introduction?'*
**Answer:** Part 2.2 of the article presents the methodological organisation of the research project. In this sense, subsection 2.2.1 develops and details the design of the three lines of research briefly mentioned in the introduction. As such, it should not be seen as a repetition but rather as a development.
3. *Reviewer: ‘Another thing that might require some consideration is on page 10. Here the butt-scarf of the keel is discussed and it is said that ‘As the keel is in contact with the water, it is understandable that a butt-scarf would generate the same principle of sealing through the swelling of the wood as it is used for the planking in the carvel technique’. However, this is only partly true as wood swells a whole lot more across the fibres than along the fibres.’ *
**Answer:** Yes, an important mechanical characteristic of the wood material is to be strongly anisotropic.  When we state that ‘a butt-scarf would generate the same principle of sealing through the swelling of the wood as it is used for the planking in the carvel technique’,  it doesn’t mean that the percentage of swelling is the same across than along the fibres. We do agree that it is a lot more across the fibre, but this doesn’t exclude that the principle of swelling to obtain waterproof is the same. And although the swelling is weaker along the fibre than tangentially, or even radially, it is to be considered that the cross-section surfaces of the keel in contact are much more considerable than the edges of the planks. For this reason, it is understandable that a butt-scarf used to join to keel timbers is more tightly sealed than a vertical flat-scarf. An additional text will be introduced to explain more in detail these considerations. To finish on this point, we would like to add that it is symptomatic to find this type of scarf on the keels of Mediterranean ships such as *Mortella III* but also *Cala Culip VI* (dated from the end of the 13th century), or even on *Cais-do Sodré* (c.1500), which although located in Atlantic waters, presents all the Mediterranean constructive characteristics. Beyond the lessons of Mediterranean archaeology, this system appears to have been widely adopted in Ibero-Atlantic construction from the end of the 16th century, since the authors quoted in our article recommend this type of keel scarf because of its better sealing than the others. As a result, it is to be mentioned that the Spanish Ordinances of the early 17th century will even impose keel butt-scarf in the State shipbuilding.

# Peer review: Reviewer response for version 1

**DOI:** 10.21956/openreseurope.15026.r28628

**Published:** 2022-03-07

**Authors:** José Antonio Bettencourt

**Affiliations:** 1 CHAM–Centre for the Humanities and History Department, FCSH, NOVA University Lisbon, Lisbon, Portugal

The development of naval archaeology in recent decades was based on the integrated study of material remains and historical data, especially for recent times. The study of the 16th-century ship Mortella III is a good example of this approach. Known since 2005, this site was the subject of an extensive interdisciplinary research program that resulted in several publications, including two books, based on the Phd Thesis of the main author of this paper.

 The paper corresponds to a review of the research, based on the results obtained in the 2019 campaign, which focused on the stern area of the hull, and included the collaboration of researchers from several European research units with different backgrounds. The existence of several previous publications influenced its structure. It follows a traditional model: the first two chapters address the investigation: chapter 1 present the archaeological site and chapter 2 the excavation and documentation methodologies; the other chapters present the results - the ship (chapter 3), including the identification of wood and the dendrochronological studies (chapter 4), the artillery (chapter 5.1), the anchors (chapter 5.2) and the ceramics (chapter 5.3); the last one (Conclusion) make an overview of the investigation. Reading allows you to follow the scientific process, being accompanied by graphic and photographic documentation of great quality. It is therefore in detail that some changes could be made to facilitate reading; some information of interest to researchers working in contexts from the same period can also be added:

1. The general description of the archaeological context (chapter 1.1) could have additional information that would allow an immediate reading of the site archaeological map. It is recommended that the general description include information on the location of the bow/ stern, as well as a short note on other elements important for the interpretation of Figure 1. For example, the hull section located at the south extremity of the site, part of the bow planking, is not discussed in the article, but corresponds to a very interesting feature, carefully described in the previously published monographs (area AF15/2; Cazenave de la Roche, 2020c, 88-90). The publication scale of the archaeological map doesn't allow reading the numbering of the ship timbers, so it is important to add information that facilitates the location of the main pieces, such as M27, the master frame, for example.
  In this chapter, it would also be interesting to add information on possible patterns of ballast organization. This is a topic that has not been investigated in detail worldwide, although experiences have revealed that the placement of ballast was subject to special care, as verified during the excavation of the Angra B vessel, for example (Bettencourt, 2017).
2. The excavation and *in situ* documentation methodologies are detailed in chapter 2. The study methodology of the various materials (artillery, anchors and ceramics) is presented in the respective chapters. This option has advantages and disadvantages. It is advantageous because a researcher interested in only one of the categories can go directly to the topic; it is disadvantageous because relevant information about the global research strategy may be lost. We leave it to the author's consideration, but the paper would benefit from a concentrated description of the methodology, including strategies for sampling the timbers or other elements of the ship, for example.
3. The paper assumes that the hull is the main research topic, presented in chapter 3. Two complimentary research perspectives are adopted – definition of the Italian-influenced Mediterranean shipbuilding model, through the study of their “fingerprints”; analysis of “architectural marks”, through the analysis of hull proportions and geometric shape definition.
  In the first perspective, purposely not much explored in the article, the authors refer to the advances made in the definition of the Ibero-Atlantic family, as a counterpoint to the delay in this approach in the Mediterranean. They mention several articles where the Mediterranean characteristics are reviewed, but do not include the reference to the pioneering work of Eric Rieth (1998).
  The hull analysis is exhaustive. The comparative study is carried out throughout the text, but it is not systematic, a justifiable option considering the paper aims. However, if this is the option, one does not understand the relevance of comparing the room and space of Mortella III with the Basque ship of Red Bay. It would probably be of greater interest to limit the comparative study to other Mediterranean cases.
  In the second approach, archaeological data are complemented with historical data, seeking to go beyond the limits of the material record. The results are always debatable, but the hypotheses presented are well-founded.
4. The artifacts chapters add interest to the paper. Their analysis follows a similar structure used in the hull study. The exhaustive description of the three selected categories is followed by an approach to written sources on the same topic, an approach that enriches the investigation.
  The authors assume the choice for the systematic study of artillery, anchors and ceramics, but other materials, such as glass and rigging components, are mentioned on the text. Without the same detail, it would be interesting to present some additional information about the other artifacts found on the site, which may be beneficial for the scientific community involved in other specific studies.
5. The article needs some minor adjustments:

- Homogenization of the reference to the chronological period, sometimes referred to as modern, others as early modern.
- On page 13, where it reads Aveiro 1, should read Ria de Aveiro A.
- On page 11, the reference to Figure 4a refers to Figure 7a.

To conclude, the study project of Mortella III shipwreck constitutes an important milestone in early modern nautical archaeology, especially in the Mediterranean. It is strongly recommended that this paper pass peer review.

If applicable, is the statistical analysis and its interpretation appropriate?

Yes

Is the study design appropriate and is the work technically sound?

Yes

Is the work clearly and accurately presented and does it engage with the current literature?

Yes

Are the conclusions drawn adequately supported by the results?

Yes

Are sufficient details of methods and analysis provided to allow replication by others?

Not applicable

Are all the source data and materials underlying the results available?

Yes

Reviewer Expertise:

Maritime archaeology

I confirm that I have read this submission and believe that I have an appropriate level of expertise to confirm that it is of an acceptable scientific standard.

## References

[1] : The Mortella III Wreck: a Spotlight on Mediterranean Shipbuilding of the 16th Century.2020; 10.30861/9781407354132 10.30861/9781407354132

[2] : A 16th century shipwreck from Angra Bay (Terceira Island, Azores, Portugal): some results from an ongoing research project. In Proceedings of the Fourteenth International Symposium on Boat and Ship Archaeology (ISBSA 14), Gdansk, 2015. *Gdansk: National Maritime Museum* .2017;115-122

[3] : Construction navale à franc-bord en Méditerranée et Atlantique (XIVe–XVIIe siècle) et “signatures architecturales”: Une première approche archéologique, In Rieth/É (ed.), Méditerranée Antique. Pêche, Navigation, Commerce. *Éditions du CTHS* .1998;177-188

# Peer review: Reviewer response for version 1

**DOI:** 10.21956/openreseurope.15026.r28327

**Published:** 2022-02-07

**Authors:** Marcel Pujol i Hamelink

**Affiliations:** 1 Higher School of Conservation and Restoration of Bens Culturals de Catalunya, Barcelona, Spain

(Translated by Montserrat Navarro Vilar)

 This article is of scientific interest in the field of archaeology and naval history. It is about the final conclusions reached by the various specialists involved in the excavation and study of The Mortella III wreck (Corsica). The excavation campaigns in the archaeological site lasted 15 years. This article is a summary of the different research stages. However, some of the results have already been presented in several publications. I would like to highlight the monograph and the thesis written by Arnaud Cazenave de la Roche (main researcher and director of this archaeological excavation).

 The text is well organized, with an internal coherence that offers a clear presentation of the results, making it easy for the reader to follow the text and understand it clearly.

 The introduction briefly explains the research activity carried out and the results presented in the development chapters of the article.

 This article has been divided into three different sections:

1. The historical and geographical context (Chapter 1. Overview of the Mortella III wreck).
2. The archaeological methodology (Chapter 2. Stakes of the excavation and working methodology).
3. The study of the material culture. In this case, the wreck was studied considering three different factors: *the ship as a maritime transport machine*, focusing on the hull (Chapter 3. The line on naval architecture and Chapter 4. Wood: Material and Dendrochronology), *its function*, in this case as an armed merchant (Chapter 5.1. The artillery) and *the crew* that made it possible for the ship to reach Corsica from Sicily. It has been possible to reach this conclusion after studying the pottery used as tableware. (5.3. Ceramic finds).

In my opinion, the section in which the anchors are studied (5.2. The anchors) should be included in Chapter 3 as the anchors are part of the ship (being the ship a maritime machine).

 The different chapters have various lengths according to the importance of the object to be studied. The chapter which talks about naval architecture stands out in the first place for its extension, while the others that deal with the cargo or even the crew have a less considerable weight, probably because the ship was unloaded before it was sunk.

 It can be said that the chapters have been written by different authors as I have noticed a certain decompensation in the use of the comparative data and the bibliographical references. From a historian’s point of view, the chapter dedicated to ceramics has the most methodological rigor as it also focuses on the time and the space.

 Looking at the article in more detail, there are some comments to be made, which may or may not be taken into consideration. However, I think it would help to improve some aspects of the work, which on the other hand is of great quality.

 The impression I get after reading the following introductory sentence " *The Mortella wrecks are witnesses to a turbulent Mediterranean history in the dawn of the modern era, characterized by the opposition of European nations in the Mediterranean*" is that there were no previous conflicts in the Mediterranean. I have to say that throughout the Late Middle Ages conflicts were continuous among the Mediterranean nations (Genoa, Pisa, Venice, Barcelona and the Crown of Aragon). Moreover, in the 13th century, some Atlantic states, such as Castile and Leon and France, played an important role in the Mediterranean geopolitics.

 An important aspect of the work is the desire to offer the reader the technical fingerprints of the Mediterranean shipbuilding. First, the architectural features of the wreck have been compared with others of Mediterranean origin from the same period (Calvi I, Villefranche I). The survival of these fingerprints in the 17 ^th^, 18 ^th^ and 19 ^th^ century wrecks have also been checked (Sardinaux or the manuscript on the construction of galleys of 1691). In my opinion, the fingerprints found in the Mortella III should have also been compared with previous shipwrecks.

 We should bear in mind that all the fingerprints of the Mediterranean naval tradition were already found and studied in several medieval shipwrecks (Culip VI, Boccalama A, Les Sorres X, Contarina I, etc.). It was determined that they had some common architectural features: a mast-step with side-keelsons, a hook scarf between the frame-floor and the futtock, an iron nail from the frame-floor to the futtock and another one from the futtock to the frame-floor, frames from the aft and the stern without a scarf between the frame-floor and the futtock, a butt-scarf between the pieces of the keel and the stem and the stern, a double keel, etc. (Pujol 1992, Rieth 1998 and Pujol 1998). All these fingerprints, found in the Mortella III wreck, were already present in wrecks from the 13th, 14th and 15th centuries.

 Determining the technical fingerprints of the Mediterranean tradition is much more important than comparing them with those of the Ibero-Atlantic tradition. Above all, it would be much interesting to highlight the features which are shared by the three great merchant ships of the 16th century (Calvi I, Villefranche I and Mortella III) and do not appear in other Mediterranean wrecks. This would clearly identify the existence of a subgroup of the Mediterranean naval tradition which should not be named *Italo-Mediterranean* since it is a redundancy as Italy has only a coast in the Mediterranean Sea. Since the contemporary wrecks that share the same fingerprints are all of Genoese origin, it would be better to call this subgroup *Italo-Ligurian*.

 The sentence “ *continuous interaction since the Middle Ages between the two nautical spaces, Mediterranean and Atlantic*” should be corrected as this did not occur until the early 14th century when the Atlantic naval technology (square sail, stern-rudder) first arrived in the Mediterranean. It was not until the early 15th century when the carvel shipbuilding or frame-first construction of the Mediterranean spread across the Atlantic and the Baltic. It would be more correct to say, "continuous interaction since *the Late Middle Ages*."

 From the very beginning of Chapter 3, the bow and the stern of the ship keep appearing in the text, but it is not mentioned how they were able to identify them. This explanation comes later (due to the appearance of the stern during one of the last campaigns). Perhaps it would be better to clarify this at the beginning, on a footnote.

 Care must be taken with the terminology used. In the 16th century, the name given to the deck in Italian, as it appears in Genoese and Venetian documents is ‘coberta’ or ‘coperta’. The same name is also used in other Mediterranean Latin languages, such as Catalan or Occitan-Provençal. The term ‘ponte’ and ‘falso ponte’ in Italian is an Atlantic term and it comes from the French and Spanish language (18th-19th century).

 The study of naval architecture also aims to detect the presence of architectural markers related to the shape and proportions of the ship. This is a promising line that will probably end up differentiating the Venetian shipbuilding from the Genoese in the large merchant ships of the 15th and 16th centuries.

 There are also some minor details that could be corrected:

  

- The wreck of Villefranche I – It sometimes appears as *Lomellina* and as the wreck of Villefranche-sur-Mer. The criteria should be unified to avoid confusion and to avoid repetition of information.
- Cais do Sodré wreck - The chronology and location of the find should be entered.
- Santiago de Galicia – The location is not mentioned.
- When talking about *shipwrecks in Croatia*, they should mention which ones they refer to.
- Portugueses (-s) shipwreck Nossa Senhora dos Martires - Shouldn’t it be Portuguese – in singular?
- Unify criteria -  Maststep / Mast step / Mast-step .
- Some captions have no author, or the author appears with the abbreviated name or in other full names (Jesús Guevara / J. Guevara). The author's name is sometimes shown in bold and sometimes is not.
- Unify criteria - Drawing credit: / Drawing / Diagram or directly the name of the author.
- Fig. 3: Instead of Mortella *IIII*, Mortella *III* should be written.

References:

 Pujol i Hamelink, Marcel; Estudi descriptiu i anàlisi del buc, In: Xavier Nieto (ed.), *Les Sorres X, un vaixell medieval al Canal Olímpic de Rem*, Barcelona: Generalitat de Catalunya, 1992, 29-49.

 Pujol i Hamelink, Marcel; Elements longitudinals, In: Xavier Nieto & Xim Raurich (eds.), *Excavacions arqueològiques subaquàtiques a Cala Culip 2. Culip VI*, Girona: CASC, 1998, 118-137.

  Rieth, Eric; Les éléments transversaux de la charpente, In: Xavier Nieto & Xim Raurich (eds.), *Excavacions arqueològiques subaquàtiques a Cala Culip 2. Culip VI*, Girona: CASC, 1998, 137-201.

 Rieth, Eric; Construction navale à franc-bord en Méditerranée et Atlantique (XIVe–XVIIe siècle) et “signatures architecturales”: Une première approche archéologique, *Méditerranée Antique.* *Pêche, Navigation, Commerce*; Paris: CTHS, 1998; 177–188.

If applicable, is the statistical analysis and its interpretation appropriate?

Yes

Is the study design appropriate and is the work technically sound?

Yes

Is the work clearly and accurately presented and does it engage with the current literature?

Yes

Are the conclusions drawn adequately supported by the results?

Yes

Are sufficient details of methods and analysis provided to allow replication by others?

Partly

Are all the source data and materials underlying the results available?

Yes

Reviewer Expertise:

Underwater archaeology. Naval archaeology. Medieval history. Mediterranean history

I confirm that I have read this submission and believe that I have an appropriate level of expertise to confirm that it is of an acceptable scientific standard, however I have significant reservations, as outlined above.

## References

[1] : Estudi descriptiu i anàlisi del buc, In: X. Nieto (ed.), Les Sorres X, un vaixell medieval al Canal Olímpic de Rem. *Barcelona: Generalitat de Catalunya* .1992;29-49

[2] : Elements longitudinals, In: X. Nieto, X. Raurich (eds.), Excavacions arqueològiques subaquàtiques a Cala Culip 2. Culip VI. *Girona: CASC* .1998;118-137

[3] : Les éléments transversaux de la charpente, In: X. Nieto, X. Raurich (eds.), Excavacions arqueològiques subaquàtiques a Cala Culip 2. Culip VI. *Girona: CASC* .1998;137-201

[4] : Construction navale à franc-bord en Méditerranée et Atlantique (XIVe–XVIIe siècle) et “signatures architecturales”: Une première approche archéologique, Méditerranée Antique. Pêche, Navigation, Commerce. *Paris: CTHS* .1998;177-188

# Response

**Published:** 2022-02-18

**Authors:** Arnaud Cazenave de la Roche

**Affiliations:** Spanish National Research Council, Spain

**Comment 1:** ‘The Mortella wrecks are witnesses to a turbulent Mediterranean history in [at] the dawn of the modern era, characterized by the opposition of European nations in the Mediterranean’. This phrase intends to express a historical fact which merely relates the Mortella shipwrecks to their cause, namely the military opposition of France and Spain during the turbulent period of the Seventh Italian War. The Mediterranean has been the scene of many conflicts throughout its history and, by this phrase, we do not mean that there was no conflict in this nautical area before this episode in other circumstances and chronology. To avoid any confusion or misinterpretation, we will re-write the sentence as follows: ‘The Mortella wrecks are witnesses to a turbulent history at the dawn of the modern era, when the great European nations were opposing in the Mediterranean.’

**Comment 2:** The second critical aspect of this review concerns the issue related to technical ‘fingerprints’. The reviewer states that ‘ *An important aspect of the work is the desire to offer the reader the technical fingerprints of the Mediterranean shipbuilding*’. In fact, although the final goal of our research programme is to characterise the Mediterranean shipbuilding in the Renaissance, the analysis of the technical ‘fingerprints’ of Mortella III architecture is not the subject of this article whose aim is clearly stated in the introduction in twofold: ‘The first [objective] is to present a synthesis of the architecture of the Mortella ship...The second ... is to review the contribution of the wreck to the material culture of the 16th century.’ Although our description of the architecture of the Mortella III wreck does not fail to pinpoint technical specificities or ‘fingerprints’ and, in some cases, refers to other ships of the same chronology, it is not the purpose of this article to compare the shipbuilding techniques of the Mortella III wreck with other wrecks in order to highlight the ‘fingerprints’ of Mediterranean construction as suggested by the reviewer. This comparative analysis cannot be treated in the context of a review article, as this one and goes far beyond its scope. It should be added that a) This subject is addressed in the monograph on The Mortella III wreck (Cazenave, 2020, 154-155). b) A development of this topic is part of the objectives of the ‘ModernShip’ project, and is the subject of a publication project that is currently underway. Regardless of the above, we wish to make it clear that our methodological approach of this matter is that the characterisation of the Mediterranean shipbuilding should take care to consider two points: 1) The study of the technical ‘fingerprints’, should be completed by the architectural characteristics of this tradition, i.e. the shapes and proportions, are essential and cannot be neglected. 2) Gathering technical characteristics common to wrecks of Mediterranean building tradition is important but, in our opinion, not enough. This step - which must be carried out with great caution in view of the poor quality of the documentation, the disparity of chronologies and the different sizes of the ships considered - should be followed by a comparison with other shipbuilding traditions. We believe that it is the only way to deeply understand its specificities. This methodological requirement is illustrated by the fact that most of the Mediterranean technical characteristics that we know today could be identified thanks to the fact that they are different from those evidenced in the Atlantic nautical area. For these reasons, we do not share the the reviewer's statement that ‘Determining the technical fingerprints of the Mediterranean tradition is much more important than comparing them with those of the Ibero-Atlantic tradition’ is, in our view, both are important, complementary and necessary.

**Comment 3: ** Issue concerning the way to name the Italian sub-group is highlighted by archaeology. Italian shipwrecks of the Renaissance belong to a global technical area which is the Mediterranean where some general technical features and architectural traits have been pinpointed by archaeology. But inside this Mediterranean main set, their Italian origin is likely to give them some particular characteristics leading to consider a subset. It is for this reason that we called this shipbuilding ‘Italo-Mediterranean’. However, we agree that, although the two words have two different meanings for us, their lay significance being redundant, another designation could be sought. Nevertheless, the suggestion of the reviewer to create an Italo-Ligurian subset raises an objection which is the same he pinpointed himself: Liguria being part of Italy, the association of the two terms doesn’t solve the redundancy of the terms. To conclude, to solve this issue, we just will end our sentence by characterising the Genoese shipbuilding: ‘Thus, the portrait of the *nave* that the excavation of the wreck has sketched out contributes to documenting Genoese naval construction.’

**Comment 4:** Concerning the sentence ‘continuous interaction since the Middle Ages between the two nautical spaces, Mediterranean and Atlantic’ should be corrected as this did not occur until the early 14th century when the Atlantic naval technology (square sail, stern-rudder) first arrived in the Mediterranean. From our point of view, in the current state of our knowledge, it seems quite uncertain to date the first nautical interactions between the Atlantic and Mediterranean nautical areas back to the 14th century. Indeed, there are many examples of interactions between these two spaces before the 14th century. In any case, to avoid any unproductive debate on this issue, which is not the subject of this article, we will replace the sentence ‘Beyond the knowledge of Mediterranean shipbuilding, it is a matter of understanding these two ‘nautical spaces’ in continuous interaction since the Middle Ages and of trying to set them out in all the complexity of their exchanges and technological transfers.’ by: ‘Beyond the knowledge of Mediterranean shipbuilding, it is a matter of understanding these two nautical areas, trying to set them out in all the complexity of their interactions and technological transfers.’

**Comment 5:** Deck Italian terminology. Section. 3.3.2 clearly states the terms used by Italian texts to characterise the decks of the *navi* in the 16th century relying on the texts of Theorodo de Nicolò, Nicolò Sagri, ‘Misure di navilii’, Genoese contracts published by Luciana Gatti. The terms ‘falso ponte’, ‘faux-pont’, ‘orlop/overlop’ and ´baos vacios’ are given at the end of this part to express the contemporary terms used to set out the concept of the first beams structure that is likely to exist on the large ships of this time, although never mentioned -so never named- in the texts. To avoid any confusion or misinterpretation, we suggest to modify the phrase ‘… **was** called *falso ponte* in Italian, *faux-pont* or *entrepont* in French, *orlop* or *overlop* in English and *baos vacíos* (i.e. ‘empty beams’) in Spanish’ by ‘ **will be later** called…’
